# Intestinal flora: A new target for traditional Chinese medicine to improve lipid metabolism disorders

**DOI:** 10.3389/fphar.2023.1134430

**Published:** 2023-03-01

**Authors:** Min Liu, Wei Shi, Yefang Huang, Yeke Wu, Keming Wu

**Affiliations:** ^1^ Department of Gynecology, Hospital of Chengdu University of Traditional Chinese Medicine, Chengdu, Sichuan, China; ^2^ Department of Stomatology, Hospital of Chengdu University of Traditional Chinese Medicine, Chengdu, Sichuan, China

**Keywords:** intestinal flora, lipid metabolism disorders, obesity, hyperlipidemia, non-alcoholic fatty liver disease, atherosclerosis, traditional Chinese medicine

## Abstract

Lipid metabolism disorders (LMD) can cause a series of metabolic diseases, including hyperlipidemia, obesity, non-alcoholic fatty liver disease (NAFLD) and atherosclerosis (AS). Its development is caused by more pathogenic factors, among which intestinal flora dysbiosis is considered to be an important pathogenic mechanism of LMD. In recent years, the research on intestinal flora has made great progress, opening up new perspectives on the occurrence and therapeutic effects of diseases. With its complex composition and wide range of targets, traditional Chinese medicine (TCM) is widely used to prevent and treat LMD. This review takes intestinal flora as a target, elaborates on the scientific connotation of TCM in the treatment of LMD, updates the therapeutic thinking of LMD, and provides a reference for clinical diagnosis and treatment.

## 1 Introduction

With the development of society and the improvement of living conditions, the incidence of lipid metabolism disorders (LMDs) is increasing year by year and showing a trend of rejuvenation, which seriously threatens human health. Abnormal lipid metabolism is a pathological process of elevated blood lipid levels and ectopic lipid deposition caused by genetic or acquired factors, and is an important risk factor for many metabolic diseases. Lipid metabolism is central to this process, and when lipid biosynthesis and degradation are abnormal, or when lipoprotein synthesis, metabolism, and transport are impaired, this can lead to disorders of lipid metabolism, contributing to hyperlipoproteinemia (Karr, 2017), obesity (Aron-Wisnewsky et al., 2021), non-alcoholic liver disease (NAFLD) (Katsiki et al., 2016), and atherosclerosis (Zhang et al., 2022b). Furthermore, dyslipidemia also has an effect on other target organs such as the brain and kidneys (Ross, 1999). Therefore, it is crucial to investigate the biological mechanisms underlying the development of LMD and to seek effective therapeutic targets for effective regulation of lipid metabolism to prevent and treat LMD.

The intestinal flora is an essential component of the intestinal micro-ecosystem and controls numerous metabolic processes with the host immune system, such as energy balance, glucose and lipid metabolism (Sonnenburg and Backhed, 2016). In recent years, numerous studies have further elucidated the connection between intestinal flora and its metabolites and LMD (Woting and Blaut, 2016; Jonsson and Backhed, 2017; Duttaroy, 2021), finding that dysregulation of intestinal flora and its metabolites contribute to the development of LMD (Ridaura et al., 2013; Matey-Hernandez et al., 2018; Jia et al., 2021). LMD can also be improved by altering the intestinal flora by fecal transplantation or probiotic administration (Falcinelli et al., 2018; Allegretti et al., 2020; Witjes et al., 2020). In addition, LMD can affect intestinal flora homeostasis and have a negative impact on the organism’s health. Numerous studies have shown that the intestinal flora structure of animals with high-fat diet (HFD) -induced LMD is altered, as evidenced by a decrease in intestinal flora diversity, a reduction in *Bacteroidetes*, and an increase in *Firmicutes*, *Proteobacteria*, and *Verrucomicrobia* (Tomas et al., 2016), or a significant decrease in the abundance of beneficial bacteria like *Bifidobacterium* and *Lactobacillus* (Wang B. et al., 2020). Meanwhile, some key intestinal metabolites such as lipopolysaccharides (LPS) (Cani et al., 2007b), bile acids (BAs) (Gillard et al., 2022), short-chain fatty acids (SCFAs) (Wang G. et al., 2021), branched-chain amino acids (BCAAs) (Nie et al., 2018) and trimethylamine-N-oxide (TMAO) (Janeiro et al., 2018) were discovered to play a role in the host’s lipid metabolism function. Therefore, intestinal flora dysbiosis may take a significant part in the progression of LMD, and reshaping the structure of intestinal flora can contribute to correcting LMD.

Studies have found that Lipid-lowering drugs can treat LMD by altering the intestinal flora. Simvastatin can alter the abundance and diversity of intestinal flora from phylum to genus level, modulate the downstream metabolic pathways of intestinal flora and ultimately exert a hypolipidemic effect (Zhang S. et al., 2020). In another research investigating the influence of intestinal flora on the atorvastatin’s hypolipidemic effect, it was found that atorvastatin significantly reduced serum levels of total cholesterol (TC) and low-density lipoprotein (LDL) in mice with intact intestinal flora but did not have the same effects in mice with depleted intestinal flora. Besides, we observed changes in the abundance of several sphingolipids after treatment with atorvastatin in mice with intact intestinal flora, which was not present in mice with depleted intestinal flora, suggesting that the lipid-lowering efficacy of atorvastatin also depends on the composition of intestinal flora before treatment (Zimmermann et al., 2020). However, in addition to the financial burden, long-term use of statins may trigger side effects such as myopathy (muscle pain or muscle weakness), hyperglycemia and liver enzyme abnormalities (Bellosta and Corsini, 2018), limiting the treatment options for patients. Therefore, it is crucial to provide patients with therapies that are effective and have few side effects.

TCM has been practiced for several thousand years in China, and now its use has expanded globally. TCM has a significant role in improving human subhealth, controlling metabolism and preventing major diseases. For a long time, TCM has been playing a significant role in improving metabolic disorders due to its multi-component and multi-target properties (Zhang et al., 2014; Zhang H. Y. et al., 2021). Research has revealed that TCM (natural medicine extracts and Chinese herbal formulas) can significantly improve the components and metabolite function of intestinal flora, as well as help to maintain of intestinal flora homeostasis, thereby regulating lipid metabolism. For example, baicalin improved HFD-induced abnormalities in glucose and lipid metabolism *via* enhancing the number of bacteria that produce SCFA (Ju et al., 2019). *Poria cocos* (*Schw.*) *Wolf* [Polyporaceae; *Poria*] water insoluble polysaccharide (WIP) improved markedly glucose and lipid metabolism in ob/ob mice, and the underlying mechanism may be associated with increased numbers of butyrate-producing bacteria *Lachnospiracea* and *Clostridium*, elevated butyrate levels in the intestine, improved intestinal barrier function, and activated the intestinal peroxisome proliferator-activated receptor γ (PPAR-γ) pathway (Sun et al., 2019). Si Miao Formula, a classic TCM formula, played an anti-NAFLD role by altering the composition of intestinal flora, especially by upping the proportion of *Akkermansia muciniphila* and down-regulating the production of pro-inflammatory proteins (Han et al., 2021). Resveratrol (RSV) inhibited TMAO production by remodeling intestinal flora, depressed the enterohepatic farnesoid X receptor/fibroblast growth factor 15 (FXR/FGF15) pathway, enhanced BA hydrolase activity, and promoted the synthesis of hepatic BAs, resulting in its anti-AS effects (Chen et al., 2016). On the other hand, intestinal flora may convert effective ingredients of TCM in a variety of ways to generate secondary metabolites, thus exerting the therapeutic effects of TCM in regulating lipid metabolism-related pathways and gene expression. It was found that treatment with Dingxin Recipes (DXR) IV resulted in increased levels of metabolites related to fatty acid metabolism, such as acetate and butyrate, downregulation of the Liver X Receptor α/sterol regulatory element-binding protein 1 (LXR-α/SREBP1) axis, decreased blood lipid levels, as well as suppression of excessive cholesterol deposition in the aorta in HFD-fed mice (Zhang et al., 2021c). Notably, lack of *Blautia* greatly reduced the cholesterol-lowering efficacy of berberine (BBR), revealing that intestinal flora plays a crucial part in BBR’s hypolipidemic impact (Wu et al., 2022).

In this review, we aimed to elucidate the role of intestinal flora and its metabolites in the occurrence and prevention of LMDs, and to systematically evaluate the multiple mechanisms involved in the regulation of intestinal flora in the improvement of LMDs in TCM, with the aim of providing new approaches for the prevention and treatment of LMDs in TCM and evidence for the development of novel anti-LMD drugs.

## 2 Relationship between intestinal flora and LMD

**FIGURE 1:**
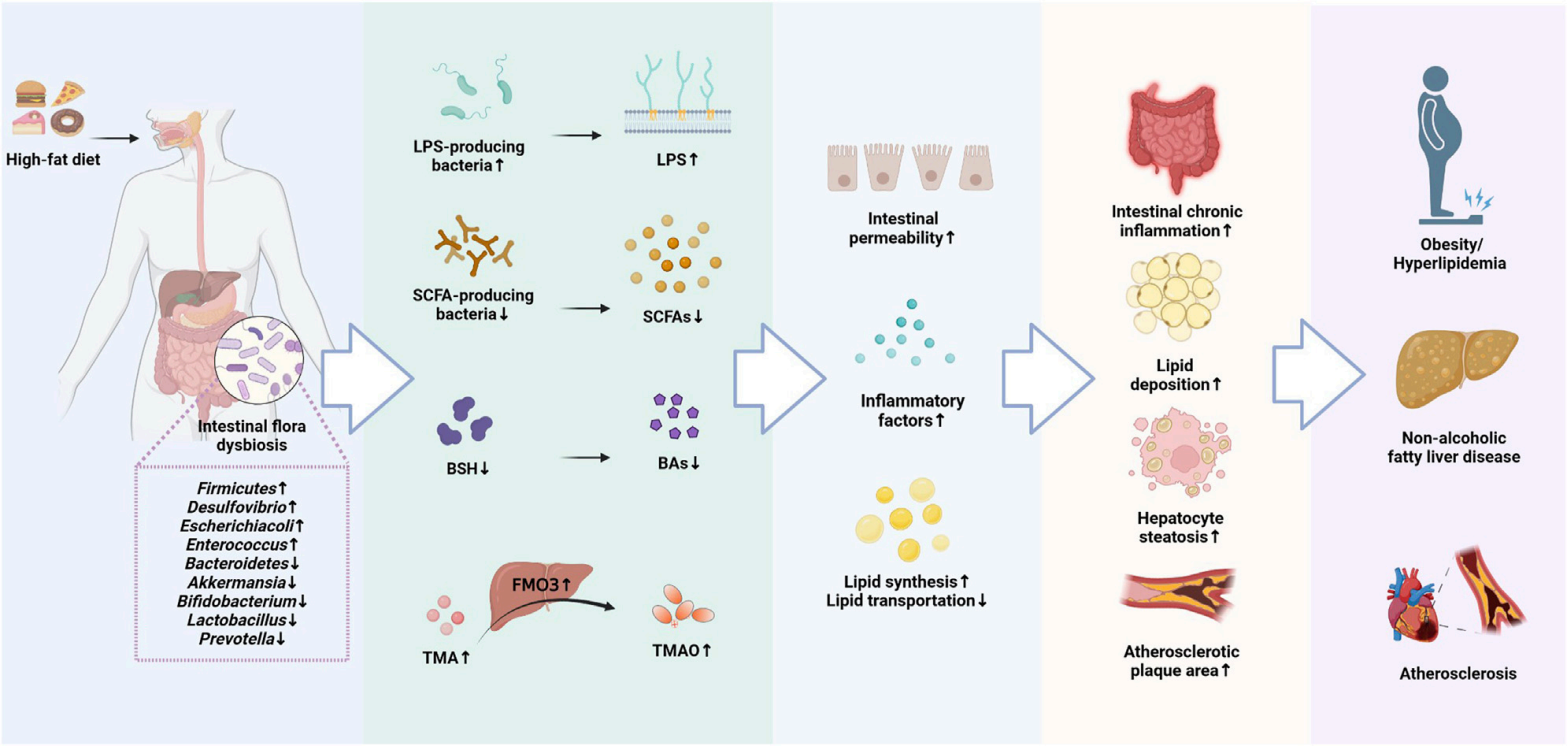
The molecular mechanisms of intestinal flora involved in the development of Lipid metabolism disorders (LMD).

A growing majority of research have revealed an interaction between lipid metabolism and intestinal flora (Matey-Hernandez et al., 2018; Aron-Wisnewsky et al., 2021). Dysbiosis of the intestinal flora can disrupt lipid metabolism, and dyslipidemia can in turn cause imbalances in intestinal flora, but the exact mechanisms between the two remain to be investigated in depth. In recent years, researchers have also identified intestinal flora metabolites that may also be involved in regulating lipid metabolism, including LPS, SCAFAs, BAs, TMAO (Liang et al., 2020; Zhou X. et al., 2021; Jia et al., 2021). These metabolites derived from the intestinal flora can act not only on the host local tissues, but also affect the host endocrine and organ functions the in various ways (Figure 1).

### 2.1 The role of intestinal flora structure in the development of LMD

Intestinal flora is an important influence on energy absorption and metabolism, and a decrease in intestinal flora diversity is associated with an increased relative risk of LMD (Rastelli et al., 2018). Studies have shown that the α diversity of intestinal flora in high-fat rats is significantly reduced, as demonstrated through a drop in the intestinal flora abundance and a decrease in the structural stability of intestinal flora (Zhao J. et al., 2022). This phenomenon is consistent with the findings on intestinal flora of patients with LMD (Mu et al., 2020), but the specific composition of intestinal flora in healthy and obese people is still debated (Kasai et al., 2015). The human intestinal flora is mainly composed of *Firmicutes* and *Bacteroidetes*, which account for more than 98% of intestinal flora abundance and are involved in lipid and BA metabolism to maintain energy balance in the host. Among them, *Firmicutes* can induce hepatic steatosis *via* regulating fatty acid inflow and lipogenesis (Chen Y. H. et al., 2019), while *Bacteroidetes* can suppress obesity by promoting the catabolism of BCAAs in brown adipose tissue (BAT) (Yoshida et al., 2021). In addition, *Bacteroidetes* also produce bile salt hydrolase (BSH), which hydrolyzes conjugated BAs and regulates BAs-mediated lipid metabolism (Tian et al., 2019). Therefore, *Firmicutes* are hypothesized to be positively correlated with LMD, and *Bacteroidetes* are negatively correlated with LMD. A lower *Firmicutes/Bacteroidetes* (*F/B*) ratio represents a healthier gut microbial environment, and the risk of obesity and LMD may be increased by an elevated *F/B* ratio (Magne et al., 2020). *Firmicutes* were increased in the intestinal tract of obese individuals compared to healthy individuals, and *F/B* ratio decreased with an increase in *Bacteroidetes* and a decrease in *Firmicutes* following weight loss treatment (Damms-Machado et al., 2015). *Akkermansia* is a mucus-degrading bacteria that reverses HFD-induced increase in adiposity, metabolic endotoxemia, and adipose tissue inflammation (Everard et al., 2013). *Bifidobacterium* also promotes changes in lipid metabolism and glucose homeostasis (Salazar et al., 2019). Long-term HFD-induced changes in the structure of intestinal flora in animal models, mainly in the form of a significant reduction in the content of beneficial bacteria like *Akkermansia*, *Bifidobacterium*, *Lactobacillus*, *Blautia*, and *Faecalibaculum*, which have the ability to alleviate LMD (Shin et al., 2014; Wang B. et al., 2020). In another study, a substantial growth in the abundance of *Bacteroides* and *Parabacteroides* was found in hyperlipidemic rats (Zhao J. et al., 2022). *Bacteroides* and *Parabacteroides* contain a large number of conditionally pathogenic bacteria that can increase the risk of infection. They can also produce acetic acid, which promotes appetite and increases food intake by activating the parasympathetic nervous system and promoting the release of insulin and ghrelin, leading to elevated TG levels, liver and muscle fat accumulation and the development of obesity-related metabolic diseases (Perry et al., 2016). LMD influenced by changes in the intestinal flora have been found extensively in animals and are also largely reflected in humans (Maruvada et al., 2017). Therefore, the management of LMD focused on the modulation of intestinal flora structure is crucial.

### 2.2 The role of LPS in the development of LMD

A normal intestinal mucosal barrier is fundamental for maintaining intestinal function. One study indicates that the pathophysiological root of LMD is a compromised intestinal barrier (Schulz et al., 2014). Damage to the tight junction proteins between intestinal epithelial cells, such as ZO-1, Occludin and Claudin1, which are essential for the intestinal mucosal barrier, leads to a rise in intercellular permeability and the admission of bacteria, endotoxins, as well as macromolecules into other tissues, organs or body circulation *via* the cellular bypass pathway, resulting in metabolic endotoxemia, inflammation and metabolic disorders (Zheng et al., 2022). LPS is the central aspect of Gram-negative (G-) bacteria’s outermost layer of cell wall, and is an important inflammatory stimulus that can cause severe damage to the intestinal barrier and contribute to a chronic inflammatory response in the body, leading to the development and progression of metabolic diseases (Zhang-Sun et al., 2015). It has been found that a HFD disrupts the structure of intestinal flora and increases the abundance of LPS-producing bacteria, such as *Desulfovibrio*, which further enhances intestinal permeability and contributes to large amounts of LPS to enter the circulation from the intestine, triggering metabolic endotoxemia (Cani et al., 2007a), which is linked to the development of LMDs including dyslipidemia, obesity, NAFLD and cardiovascular disease (Manco et al., 2010). LPS can exacerbate lipid metabolism by binding to Toll-like receptor 4 (TLR4), a receptor expressed on macrophages, hepatocytes and adipocytes, and inducing the secretion of pro-inflammatory factors like interleukin 6 (IL-6) and monocyte chemotactic protein 1 (MCP-1). Secretion, exacerbating lipid metabolic disorders (Robbins et al., 2014). Furthermore, LPS increases intestinal permeability through chronic inflammation mediated by MAPK or NF-κB pathways (Zhou et al., 2018), triggering circulating metabolic endotoxemia and leading to lipid dysfunction (Sultana et al., 2016). Myeloid differentiation factor 88 (MyD88), a central adapter molecule for most TLRs, regulates fat storage and inflammatory responses (Everard et al., 2014), influences the activity of transcription factors related to lipid metabolism and BA profiles, and is involved in regulating the body’s inflammatory response and lipid metabolism (Duparc et al., 2017). Additionally, LPS forms a triple complex with CD14 and LPS-binding protein (LBP), which activates the MyD88/NF-B pathway and increases the production of numerous inflammatory proteins. Moreover, cluster of differentiation 36 (CD36) is an important lipid absorption regulator in the intestine, and its expression is mediated by derivatives of *Desulfovibrio* and *Clostridium*. Loss of CD36 may lead to excessive lipid accumulation and activation of inflammatory factors, triggering the risk of LMD (Plociennikowska et al., 2015; Petersen et al., 2019). Thus, chronic low-level inflammation in the organism may be a key link in the pathogenesis of LMD, and disorders of intestinal flora may also contribute to chronic inflammation.

### 2.3 The role of SCFAs in the development of LMD

SCFAs are metabolites derived from the fermentation of undigested carbohydrates or proteins by intestinal flora and include butyrate and acetate, propionate. Among them, butyrate is primarily performed by *Firmicutes*, while acetate and propionate are mostly generated by bacteria belonging to *Bacteroidetes* (Walker et al., 2005), and they can play a crucial part in controlling host appetite, lipid metabolism, inflammatory response, and maintenance of energy balance through various pathways (Sanchez et al., 2020). SCFAs, represented by butyric acid and propionic acid, not only stimulate the release of leptin from adipose tissue, suppressing hunger and reducing host feeding activity (Naraoka et al., 2018), but also inhibit hepatic FAS activity and reduce serum lipid (TG and TC) levels (Larkin et al., 2009). As key regulators of host lipid metabolism, about 95% of SCFAs are absorbed in the intestine or utilized by intestinal flora, acting as important regulators of host lipid metabolism. These molecules not only providing the host energy, but also acting as signal transduction molecules to initiate G protein-coupled receptors 43 and 41 (GPR43 and GPR41) in intestinal epithelial cells. Activated GPR43 and GPR41 promote the secretion of peptide tyrosine-tyrosine (PYY) and glucagon-like peptide-1 (GLP-1) from enteroendocrine L cells, which reduce appetite, increase energy release and inhibit adipocyte synthesis through the “brain-gut axis” (Eslick et al., 2022). SCFAs also promote beige lipogenesis, leading to enhanced fat oxidation and energy expenditure, a process associated with the activation of GPR43 or GPR41 (Lu et al., 2016). And butyric acid also improves serum lipid metabolism by increasing fatty acid oxidation in brown adipose tissue (BAT) through thermogenesis, significantly reducing serum TG levels (Li Z. et al., 2018). In addition, SCFAs may also be involved in lipid metabolism by regulating related pathways and gene expression. For example, butyric acid downregulates the expression of proliferator-activated receptor γ (PPARγ), upregulates the expression of uncoupling protein 2 (UCP2), promotes mitochondrial proton efflux, which in turn activates the AMPK pathway, decreases lipid synthesis, and increases lipid oxidation. Acetic acid decreases SREBP-1 expression and ATP citrate lyase (ATP-CL) mRNA levels, decreases acetyl coenzyme A supply, and contributes to a decrease in cholesterol and fatty acid synthesis; it also enhances acyl-CoA oxidase (AOX) gene expression, promotes fatty acid β-oxidation, and increases energy expenditure (Fushimi et al., 2006). While another study speculated that acetate regulation of lipid metabolism may be associated with an increase in the AMPK/PGC-1α/PPARα axis (Araujo et al., 2020). Additionally, SCFAs can regulate the intestinal barrier and inflammatory levels and are indirectly implicated in lipid metabolism regulation. According to studies, SCFAs can elevate intestinal trans-epithelial resistance and reduce LPS production, thereby upregulating tight junction protein expression and mucin secretion, reducing intestinal mucosal permeability and alleviating intestinal barrier disorders (Zhao et al., 2018). Meanwhile, SCFAs inhibit the expression of TNF-α/NF-κB inflammation-related genes, reduce the secretion of pro-inflammatory molecules like IL-6 and IL-12, as well as increase the secretion of anti-inflammatory molecules like IL-10 and IL-4, alleviating chronic inflammatory response and avoiding the formation of hyperlipidemia (Chang et al., 2014; Yuan et al., 2019).

### 2.4 The role of BAs in the development of LMD

The synthesis of BAs is the main pathway of cholesterol degradation and metabolism in humans, which is regulated by intestinal flora. The majority of primary BAs are converted from cholesterol by the enzymatic action of three cholesterol hydroxylases, CYP7A1, CYP8B1 and CYP27A1 in the liver (classical pathway), and a small proportion is processed by CYP27A1 and CYP7B1 in extrahepatic sites (alternative pathway). Primary BAs, which including cholic acid (CA) and chenodeoxycholic acid (CDCA), can bind to glycine or taurine to form conjugated BAs like glycocholic acid (GCA), glycochenodeoxycholic acid (GCDCA), taurocholic acid (TCA) and taurochenodeoxycholic acid (TCDCA). The bile salt export pump (BSEP) or ATP-binding cassette subfamily G member 5 (ABCG5)/ABCG8 subsequently actively transfers these conjugated BAs into the bile, where they contribute to lipid emulsification when the food enters the intestine (Jia et al., 2018). The apical sodium-dependent bile acid transporter (ASBT) reabsorbs around 95% of BAs from the intestine; The apical sodium-dependent bile acid transporter (ASBT) reabsorbs around 95% of BAs from the intestine; these are subsequently released into the portal circulation by the organic solute transporter α/β (OST α/β), which finally transports them back to the liver (Vourakis et al., 2021). Intestinal flora can reduce cholesterol levels by producing BSH and CYP7A1, which decouple conjugated BAs into unconjugated BAs including deoxycholic acid (DCA) and lithocholic acid (LCA), preventing their reabsorption through ASBT and promoting BAs excretion through the feces (Wahlström et al., 2016). It is known that BSH-producing bacteria include *Bacteroides*, *Bifidobacterium*, *Clostridium*, *Eubacterium* and *Lactobacillus*, while *Clostridium* and *Eubacterium* also possess 7α-dehydroxylation activity (Vourakis et al., 2021). BAs can also act as signaling molecules, such as conjugated BA T-βMCA, unconjugated BAs CDCA and LCA, which regulate lipid metabolism by interacting to the BA receptors like FXR and Takeda G protein-coupled receptor 5 (TGR5) to regulate BA self-synthesis, transport, as well as lipid digestion and absorption (Schoeler and Caesar, 2019). In summary, BAs play an important part in lipid metabolism homeostasis. Increasing intestinal BA production can block the reverse cycle from BA to cholesterol; it can also accelerate the transformation of cholesterol to BA, lowering serum TC levels. It is worth noting that the synthesis and excretion of BAs are related to the regulation of intestinal flora.

### 2.5 The role of TMAO in the development of LMD

Trimethylamine oxide (TMAO) is an intestinal flora-dependent metabolite formed by the oxidation of trimethylamine (TMA) in the liver by flavin-containing monooxygenase (FMO), especially FMO3 (Chen Y. et al., 2019). Studies have shown that serum TMAO levels are determined by genetics, diet and intestinal flora, and may be a biomarker of AS (Randrianarisoa et al., 2016; Canyelles et al., 2018). Another observational study found that TMAO levels were positively and significantly related with body mass index (BMI), fatty liver index and visceral obesity index, and could be used as a predictor of NAFLD and metabolic syndrome (Barrea et al., 2018). Additionally, 8 consecutive weeks of supplementation with a high TMAO diet may lead to elevated the levels of plasma TC, TG and LDL-C and induce hyperlipidemia (Koeth et al., 2013). Knockdown of FMO3 may downregulate circulating TMAO levels and acts as a preventive measure against hyperlipidemia (Miao et al., 2015). As a result, intestinal flora metabolite TMAO is essential for lipid metabolism. The underlying mechanism of TMAO-induced disruption of lipid metabolism is unknown; however, it may be associated with host cholesterol and BA metabolism. It has been investigated that TMAO reduces the expression of CYP7A1 and CYP27A1, two key enzymes necessary for the synthesis of BAs, and various BA transport proteins, decreases BA biosynthesis, inhibits the reverse cholesterol transport (RCT) pathway, and affects BA metabolism and cholesterol homeostasis in the hepatic-intestinal tract (Koeth et al., 2019). In turn, BAs can also lead to an increase in serum TMAO by mediating the upregulation of FMO3 expression by FXR (Bennett et al., 2013). Moreover, TMAO is able to induce CD36 and scavenger receptor A1 (SR-A1), which are involved in stimulating macrophages to bind ox-LDL, promoting macrophage foaminess and causing intracellular cholesterol accumulation (Wang et al., 2011). This process may also be mediated through MAPK and NF-κB signaling pathways that promote vascular inflammation, one of the earliest cellular signals in the atherosclerotic process (Ma et al., 2017; Geng et al., 2018).

### 2.6 The role of intestinal flora and its metabolites in the clinical monitoring and treatment of LMD

Obesity, NAFLD and AS are frequently associated with LMD. Therefore, for the efficient implementation of atherosclerotic cardiovascular disease (ASCVD) preventive and treatment methods, early diagnosis of dyslipidemia and monitoring changes in its levels are key foundations.

Routine lipid testing to identify the “hidden” high-risk groups will not only facilitate better treatment decisions, but more importantly, it will provide early intervention for these high-risk individuals or patients with LMD, so that they can have more cardiovascular benefit. The basic items of clinical lipid monitoring are TC, TG, LDL-C and HDL-C. It is noteworthy that LDL-C is an important causative factor of ASCVD and has an important significance in lipid monitoring. More and more clinical laboratories are using ApoAⅠ, ApoB, and Lp(a) as routine lipid testing items. In addition, the clinical testing items of oxLDL, FFA and non-HDL-C are also receiving increasing attention. In recent years, assays for intestinal flora and its metabolites have also been increasingly used to assist in monitoring lipid levels. Usually healthy people have low *F/B* ratio, while patients with LMD tend to have high *F/B* ratio (Human Microbiome Project, 2012). The main core of LMD such as hyperlipidemia is chronic low-level inflammation in the body, which is also caused by intestinal flora, often manifested as elevated serum LPS levels (Dai and Fu, 2019). SCFA is inversely correlated with lipid levels and can reduce serum TG and TC levels by inhibiting FAS activity (Larkin et al., 2009). BA is produced by cholesterol conversion, and its increased synthesis and impaired transport frequently lead to the development of LMD (Haeusler et al., 2016). Serum TMAO levels predict the risk of cardiovascular disease and are closely related to the regulation of cholesterol (Chen et al., 2019). Therefore, the detection of intestinal flora and its metabolites can also be used as a reference item for clinical lipid monitoring, but it still needs to be combined with the items of lipid testing for comprehensive assessment, so it is not yet popular in the clinic.

Currently, the guidelines recommend three main classes of lipid-lowering drugs (Du, 2022): 1) Statins, which reduce cholesterol synthesis in hepatocytes mainly through competitive inhibition of endogenous cholesterol synthesis rate-limiting enzymes, leading to a significant decrease in blood cholesterol levels, and are the cornerstone of primary prevention of ASCVD. 2) Ezetimibe, which reduces cholesterol by inhibiting the absorption of cholesterol in the intestinal tract. 3) Proprotein convertase subtilisin/kexin type 9 (PCSK9) inhibitors, which lower cholesterol levels by inhibiting the binding of PCSK9 to LDL receptors and preventing LDL receptor degradation, are the most potent cholesterol-lowering drugs available and have a good safety profile, offering another option for high-risk patients who are statin intolerant (Koren et al., 2019). The intestinal flora and its metabolites serve as a key link in lipid metabolism, and the therapeutic effect of lipid-lowering drugs may be related to the regulation of intestinal flora. Statins were found to modulate the abundance of SCFA, SBA, TMAO and LPS-producing intestinal flora, which in turn affected AMPK-SREBP, FXR, PXR, FFAR2 and TLR4-Myd88 signaling pathways, acting to regulate cholesterol metabolism and LDL-C levels (Sun et al., 2022). In addition, different statins, such as atorvastatin, simvastatin and resevastatin, have different effects on different intestinal flora. Desulfurization was negatively correlated with TG and HDL-C levels, and desulfurization was significantly reduced after ezetimibe intervention (Jin et al., 2022). *Desulfovibrio* was considerably decreased following ezetimibe treatment and had a negative correlation with TG and HDL-C levels (Jin et al., 2022). PCSK9 inhibitors can promote the clearance of LDL-C and LPS from the blood, regulate intestinal microecology, alleviate intestinal inflammation and endotoxemia, and reduce the risk of cardiovascular disease (Morelli et al., 2019). Furthermore, probiotic supplementation alone was able to significantly reduce plaque area in the full-length aorta and aortic sinus, lower plasma TMAO and cecum TMA levels, improve lipid disorders, reduce serum oxLDL and inflammatory factor levels in mice, increase cecum acetate and butyric acid levels, and reduce inflammatory responses in the aorta and liver of mice by inhibiting the levels of key proteins in the TLR4/MyD88/NF-kB/NLRP3 pathway (Wang, 2022). In contrast, mice on diets supplemented with choline or TMAO showed increased cholesterol levels in peritoneal macrophages, elevated TMAO plasma levels, and enhanced aortic atherosclerotic plaques (Wang et al., 2011). Thus, intestinal flora and its metabolites also play an important role in the treatment of LMD, but further studies are needed to validate this in order to maximize the clinical benefits of drugs.

## 3 Mechanisms of regulating intestinal flora in TCM for LMD

**FIGURE 2:**
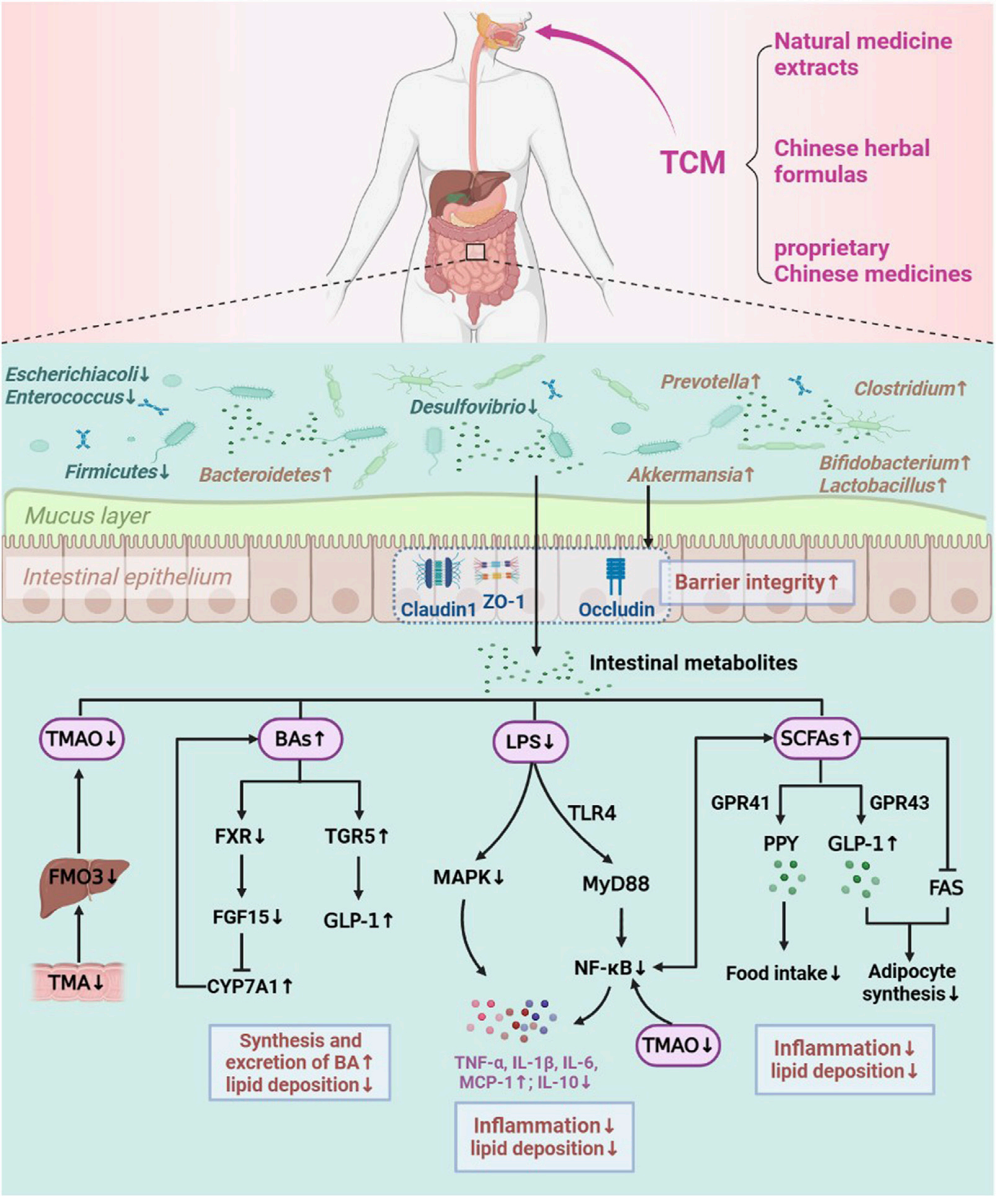
Mechanisms of TCM in improving LMD by regulating the intestinal flora.

TCM is widely utilized to treat metabolic diseases in China. The effective components of TCM can directly interact with the intestinal flora once they enter the intestinal system by oral administration and produce therapeutic effects. Studies have shown that TCM (natural medicine extracts, Chinese herbal formulas and proprietary Chinese medicines) can effectively modify the host intestinal flora structure and its metabolite levels, repair the intestinal mucosal barrier, reduce inflammatory infiltration, as well as regulate lipid metabolism-related pathways and genes expression to correct LMD (Dai and Fu, 2019). Therefore, we systematically summarized the possible mechanisms involved in TCM to modulate intestinal flora and its metabolites in order to prevent and treat LMD (obesity, hyperlipidemia, NAFLD, and AS) (Figure 2).

### 3.1 TCM remodels the structure of intestinal flora to improve LMD

TCM treatment encourages the development of beneficial intestinal bacteria by increasing the diversity of intestinal flora, while inhibiting the growth of harmful bacteria (Ding K. et al., 2019; Ling et al., 2022). It was found that intervention with Zexie Tang (ZXT) (Xu et al., 2017), Shenlingbaizhu Powder (SLBZ) (Hong, 2021), *Ganoderma lucidum* polysaccharide (GLP) (Sang et al., 2021), *G. lucidum* polysaccharide and chitosan (PC) (Tong et al., 2020), Pueraria lobata starch (PLS) (Yang Y. et al., 2022), Luteolin (Liu, 2022), Naringin (Wang F. et al., 2020), Inulin (Bao, 2021; Cui et al., 2022), Epigallocatechin gallate (EGCG) (Zuo, 2020), RSV (Chen et al., 2016) greatly enriched the abundance of *Bifidobacterium* in the intestinal tract; Modified Xiongdan yinchen granules (MXYG) (Wu et al., 2021), Tian Huang Formula (THF) (Pang, 2021), Tea seed saponins (Lin et al., 2020), Kaempferol (Wang T. et al., 2020), Green Brick Tea (Zhou T. T. et al., 2021) were able to upregulate the relative abundance of *Lactobacillus*; the intervention of Yunpi Huazhuo granules (YPHZ) (Kou et al., 2022), Qinggan Qushi Huoxie prescription (QGQSHX) (Zhang X. Y. et al., 2022), Jiangzhi Ligan Decoction (JZLG) (Tang et al., 2016), Modfied Yinchen Wuling San (MYCWL) (Xu et al., 2019), Purple yam (*Dioscorea alata L.*) resistant starch (PYRS) (Li T. et al., 2019) and PLS (Yang Y. et al., 2022) also increased the abundance of intestinal *Bifidobacterium* and *Lactobacillus*, resulting in an increase in the proportion of beneficial bacteria and a corresponding increase in its metabolites, such as acetate and lactate, thus lowering the PH in the intestine and greatly inhibiting the development of harmful bacteria such as *Parabacteroides*, *Desulfovibrio*, *Escherichiacoli*, *Enterococcus* and *Helicobacter*. Interestingly, we also found that *Alistipes* had a negatively relationship with serum lipid profile, while PC (Tong et al., 2020) and Porphyran (Wang X. et al., 2022) were able to reduce lipid levels by upregulating the abundance of *Alistipes*. *Blautia* was negatively correlated with visceral fat area, and the number of *Blautia* was noticeably increased by *Ilex pubescens* triterpenoid saponins (IPTS) (Ba et al., 2021) treatment. *Akkermansia* was negatively correlated with inflammation levels, while *Alisma orientale* extract (Li L. S. et al., 2019), Jaboticaba peel and seed powder (JPSP) (Soares et al., 2021) were able to elevate the relative abundance of *Akkermansia* and reduce inflammation levels and lipid accumulation. *Faecalibaculum*, a SCFAs-producing bacteria, while Burdock inulin (Wang, 2020) and Yinchenhao Decoction (Li Z. H. et al., 2019) can upregulate the relative abundance of *Faecalibaculum* to promote the production of SCFAs and alleviate inflammation to improve lipid metabolism. In contrast, the opposite result was observed after Caffeic acid (Mu et al., 2021) treatment, which showed a decrease in the abundance of *Faecalibaculum*. This suggests that it may be the role of multiple intestinal flora in regulating lipid metabolism and inflammation levels, and that *Faecalibaculum* may not be a core flora.

In addition, the effectiveness of TCM in reversing the elevated *F/B* ratio in animals with a HFD has been experimentally confirmed. It was found that some natural medicine extracts, proprietary Chinese medicines and Chinese herbal formulas reduce *F/B* ratio by decreasing Firmicutes abundance and increasing Bacteroidetes abundance, such as Guizhi Tang (GZT) (Yuan et al., 2021), Naoxintong Capsule (NXT) (Lu et al., 2022), Huanglian Jiedu Decoction (HLJD) (Jiang et al., 2021), Quyu Huatan Tongmai Prescription (QYHTTM) (Miao et al., 2022), Danggui Shaoyao San (DGSY) (Yu et al., 2021), *Laminaria japonica* polysaccharide (LJP) (Zhang et al., 2021d), Porphyran-derived oligosaccharides (PYOs) (Wang X. et al., 2021), Ethyl Acetate Extract of *Eleutherococcus senticosus* (*Rupr. & Maxim.*) *Maxim.* [Araliaceae, *Acanthopanacis senticosi radix et rhizoma seu caulis*] (Jia et al., 2022) and *Lonicera caerulea L. berry* polyphenols (LCBP) (Wu et al., 2018). In contrast, the abundance of *Firmicutes* and *Bacteroidetes* were not mentioned after applying interventions such as Jieyu Qutan Huazhuo Prescription (JYQTHZ) (Li N. et al., 2021; Li N. et al., 2022), Shenerjiangzhi formulation (SEJZ) (Zhang et al., 2022c), Jian Pi Tiao Gan Yin (JPTGY) (Dong et al., 2022), Shanmei (SM) Capsule (Du et al., 2022), Tongxinluo (TXL) Capsule (Qi et al., 2022), Hugan Qingzhi Tablet (HGQZ) (Tang et al., 2018; Tang, 2019), Compound Danshen Dripping Pills (Zhang Y. Y., 2020), Jiangan Jiangzhi Pill (JGJZ) (Zhao Z. et al., 2022), Jiangzhi Granules (JZG) (Wang R. R. et al., 2021), Procyanidin B2 (PB2) (Xing et al., 2019), Resistant starch (RS) (Shou, 2021), Tea polyphenol (TP) (Wang et al., 2018) and *Gynostemma pentaphyllum (Thunb.) Makino* [Cucurbitaceae; *Gynostemmatis herba*] (GP) (Huang et al., 2018), and only a decrease in *F/B* ratio was reported. It has been suggested that one of the pathways in which TCM regulates LMDs is through a relative decrease in the *F/B* ratio of the intestine. However, it remains to be further investigated exactly by which bacteria are regulated, or which of the many genera affected by TCM have the greatest effect on *F/B* ratio by alteration.

### 3.2 TCM restores intestinal barrier and alleviates LPS-induced inflammation to improve LMD

Studies have found that TCMs are effective in improving the inflammatory response related to dyslipidemia, possibly by maintaining intestinal barrier and reducing the release of inflammatory factors. Some natural medicine extracts, proprietary Chinese medicines and Chinese herbal formulas, such as Xiexin Decoction (XXD) (Chen, 2021), Biejia Jian Wan (BJJW) (Qiu et al., 2017), RSV (Yao, 2017) increased occludin mRNA expression; Dengzhan Shengmai Capsules (DZSM) (Guo et al., 2022), *Senna tora* (*L.*) *Roxb.* [Fabaceae; *Cassiae semen*] (ST) (Luo et al., 2021), Astragalus mongholicus polysaccharides (mAPS) (Zhong M. et al., 2022), *Lycium barbarum* polysaccharide (LBPs) (Gao et al., 2021), Inulin (Perez-Monter et al., 2022), EGCG (Zuo, 2020), *Usnea diffracta Vain.* [Usneaceae; *U. diffracta*] (UD) (Zhang L. D. et al., 2021) and *Momordica charantia L.* [Cucurbitaceae; *Fructus momordicae*] (MC) (Bai, 2019) also significantly enhanced occludin and ZO-1 expression, thus significantly enhancing intestinal barrier integrity. Huayu Qutan Formula (HYQT) (Zheng et al., 2022), Gegen Qinlian Decoction (GGQL) (Liu, 2019), Diammonium glycyrrhizinate (DG) (Li Y. et al., 2018) and GLP (Sang et al., 2021) significantly upregulated Claudin-1, Occludin and ZO-1 expression, which repaired intestinal mucosal barrier and prevented the development of chronic inflammation and LMD. Moreover, WIP (Sun et al., 2019) and JZG (Wang R. R. et al., 2021) elevated the expression of mucosal integrity protein Muc5 in addition to ZO-1 and occluding has also been reported to increase the expression of Muc2 and Muc4, maintain intestinal mucosal integrity, and improve intestinal inflammation (Li W. et al., 2019). *Akkermansia,* a mucin-degrading bacteria present in the mucosal layer, it can produce certain enzymes that affect the expression of mucins and tight junction proteins to regulate the intestinal barrier function. RSV (Chen, 2019; Chen et al., 2020), BBR (Zhu et al., 2018) and *Rosa roxburghii Tratt* polysaccharide (RTFP) (Zhang p. et al., 2022) were able to enrich the abundance of *Akkermansia*, and increase the colonic mucus layer thickness and intestinal tight junction proteins expression, thus reducing intestinal hypometabolic endotoxemia. Therefore, all of these TCMs can enhance intestinal barrier integrity and reduce intestinal permeability, thereby improving intestinal inflammation and abnormal lipid metabolism.

The abundance of *Desulfovibrio*, a producer of LPS, was positively correlated with inflammation and LMD. Danlou Tablet (DLT) (Sun et al., 2020), DZSM (Guo et al., 2022), GQD (Liu, 2019), SLBZ (Zhang et al., 2018), DG (Li Y. et al., 2018), Chitooligosaccharide (COSM) (Feng et al., 2022), *Rhizoma Coptidis* (RC) alkaloids (He et al., 2016), Baicalin (Liu, 2016), Quercetin (Porras et al., 2017) and BBR (Xu, 2013) might ameliorate LMD by decreasing the abundance of *Desulfovibrio* to reduce LPS into the blood and inflammatory factors secretion. Notably, DLT (Sun et al., 2020), Gastrodin (Liu F. Y. et al., 2021) could downregulate the levels of inflammatory markers IL-1β, ICAM-1 and TNF-α, favoring the reversal of periaortic inflammation and reduction of plaque area in AS mice. By reducing LPS levels, EGCG (Li, 2020; Zuo, 2020), Citrus Peel Powder extract (CPP) (Wang H. et al., 2022), HGQZ (Tang et al., 2018), Si Ni San (SNS) (Zhu et al., 2019), *Rosa Laevigata Michx. Fruits* Polysaccharides (RLPs) (Zhang X. J., 2020), Honokiol (Ding Y. et al., 2019), Fucoidan (Huang J. L., 2021) and ST (Luo et al., 2021) were found to inhibit the excessive production of inflammatory cytokines (mainly TNF-α, IL-6, IL-1β and MCP-1). In contrast, Alisma orientalis Beverage (AOB) (Zhu, 2021), Chaihu Shugan san (CHSG) (Xie et al., 2021), RSV (Chen, 2019), ST (Luo et al., 2021), Zhibitai Capsule (ZBT) (Pan et al., 2020), Xiaoyao San (XYS) (Zhou, 2020), JGJZ (Zhao Z. et al., 2022), Qinghua Fang (QHF) (Wang Y. et al., 2021), RTFP (Zhang p. et al., 2022), LBPs (Gao et al., 2021), Noni fruit polysaccharide (NFP) (Yang et al., 2020), Inulin (Bao, 2021; Wang L. J. et al., 2021), Quercetin (Wu et al., 2019), BBR (Wu et al., 2020), GP (Shen et al., 2020), CPP (Hu et al., 2021), Paeonol (Pae) (Jiang, 2019) and Blackberry leaf and fruit extracts (BLF) (Park et al., 2019) had effective antagonistic effects on the secretion of pro-inflammatory factors (e.g., IL-6, IL-1β and TNF-α) although the effects on LPS levels were not mentioned. In addition to this, some natural medicine extracts also have a promoting effect on anti-inflammatory cytokines IL-10, such as RSV, LBPs, NFP, COSM, BBR and Inulin.

Numerous experimental studies have demonstrated that TCMs achieve improvement of LMD by down-regulating inflammation-related signaling pathways (Li, 2015). Huazhi-Rougan formula (HZRG) (Li C. et al., 2022), DZSM (Guo et al., 2022), mAPS (Zhong M. et al., 2022), Luteolin (Liu, 2022) and Myricetin (Sun et al., 2021) markedly reduced metabolic endotoxemia and chronic hypo-inflammation associated with LPS, downregulated TLR4 mRNA and protein expression, inhibited IKKβ phosphorylation, and prevented p65 NF-κB translocation to the nucleus by modulating the TLR4/NF-κB pathway. CA (Mu et al., 2021) decreased LPS/TLR4, an LPS-mediated inflammatory pathway, and reduced TNF-α, IL-6 levels, thereby inhibiting dysregulation of lipid metabolism. LBPs (Gao et al., 2021) and COSM (Feng et al., 2022) inhibited indicators related to hepatic LPS/TLR4/NF-κB signaling pathway and attenuated the level of inflammation. HQT (Tang, 2019) and Compound Danshen Dripping Pills (Zhang Y. Y., 2020) selectively targeted TLR4 and inhibited LPS-induced inflammatory mediator production by attenuating the LPS/TLR4/MyD88 pathway. SLBZ (Zhang et al., 2018) and polyphenol-rich loquat fruit extract (LFP) (Li W. et al., 2019) also exhibited downregulation of the TLR4/MyD88 pathway and its downstream molecules, thereby inhibiting the overproduction of serum lipids. While, Qiang Gan formula extract (QGE) (Li et al., 2020), GLP (Sang et al., 2021) and EGCG (Zuo, 2020) regulated lipid metabolism-related processes by reducing the expression of LPS, LBP, and CD14, inhibiting TLR4/MyD88/NF-κB signaling pathway, reducing inflammatory factors secretion, and improving endotoxemia. Si Miao Formula (SMF) (Han et al., 2021), TXL (Qi et al., 2022) and Pae (Liu, 2021) inhibited the expression of NLRP3 inflammatory vesicles and their downstream factors including IL-18, IL-1β and caspase-1, alleviated the inflammatory response and reduced lipid levels. Furthermore, MC also inhibited LPS intestinal leakage, elevated intestinal tight junction protein expression, and protected intestinal mucosal integrity, which was associated with inhibition of the intestinal NF-κB/JNK/MAPKs pathway (Bai, 2019). Notably, *Luffa cylindrica* (*L.*) *Roem* [Cucurbitaceae; *Luffa aegyptiaca Miller*] (LC) (Zhang et al., 2019) and Berberrubine (BRB) (Yang S. et al., 2022) resulted in genes associated with hepatic lipid metabolism expression reduction, including fatty acid synthase (FAS), fatty acid synthesis protein (FABP), fatty acid transport protein (FATP), SREBP-1c and CD36, thus acting to reduce lipid levels.

### 3.3 TCM modulates the level of SCFAs to improve LMD

Although the strong connection between intestinal flora, SCFAs and LMDs are still unclear, an increasing number of studies have shown that LMDs are directly correlated with both the number of SCFA-producing bacteria and the level of SCFAs. To play its part in treating LMDs, TCM can control the prevalence of bacteria that produce SCFAs and encourage the generation of SCFAs. LC (Zhang et al., 2019), DLT (Sun et al., 2020), NXT (Lu et al., 2022), IPTS (Ba et al., 2022), LJP (Zhang et al., 2021d), LBPs (Gao et al., 2021), *Zanthoxylum bungeanum Maxim.* [Rutaceae; *Zanthoxyli pericarpium*] (ZB) (You, 2016), Ginkgo biloba extract (GbE) (Wang Y. et al., 2022), Inulin (Bao, 2021; Wang L. J. et al., 2021), NFP (Yang et al., 2020), TP (Wang et al., 2018), Bilberry anthocyanins (Nakano et al., 2020) and COSM (Feng et al., 2022) significantly improved serum lipid levels (TC, TG) as well as reducing lipid accumulation, which may be associated with modifying intestinal flora and increasing SCFAs levels. In-depth research revealed that Compound Danshen Dripping Pills (Zhang Y. Y., 2020) and JZG (Wang R. R. et al., 2021) intervention elevated the abundance of *Lachnospiraceae*; Qingxin Jieyu Granule (QXJY) (Wang, 2019) and water insoluble polysaccharide WIP (Sun et al., 2019) intervention increased *Clostridium* abundance; Macroalgae Laminaria japonica (MLJ) (Zhang Q. et al., 2020), Quercetin (Porras et al., 2017), Myricetin (Sun et al., 2021), MC (Bai, 2019) and GLP (Sang et al., 2021) administration improved the abundance of *Allobaculum*; Erchen decoction (ECD) (Liu H. et al., 2021) and EGCG (Zuo, 2020) administration enhanced the abundance of *Roseburia*; HGQZ (Tang, 2019) and DG (Li Y. et al., 2018) treatment increased the abundance of *Ruminococcaceae*; SLBZ (Hong, 2021) and DZSM (Guo et al., 2022) administration enriched the abundance of *Bifidobacterium, Lactobacillus*; Guanxinning Tablet (GXNT) (Yang Q. et al., 2022), Black tea polyphenols (BTP) (Henning et al., 2018) and RLPs (Zhang X. J., 2020) treatment all increased the relative abundance of *Prevotella*; *G. pentaphyllum* saponins (GPS) (Zhong F. W. et al., 2022) administration enriched the abundance of *Bacteroides*. The above mentioned *Ruminococcaceae, Lachnospiraceae, Clostridium, Allobaculum, Bifidobacterium, Lactobacillus, Prevotella, Roseburia, Bacteroides* are all SCFAs-producing bacteria. In addition, TCM can promote the synthesis of key synthetic enzymes of SCFAs to elevate the level of SCFAs, or mediate SCFAs to regulate energy metabolism and inflammation-related pathways to improve LMD. GXNT treatment was also found to promote the production of butyrate kinase, propionate kinase, the key synthetic enzymes of SCFAs, thereby increasing the levels of butyric acid and propionic acid (Yang Q. et al., 2022). BTP treatment increased the level of SCFAs and activated the AMPK signaling pathway to increase energy expenditure, thus exerting a fat-lowering and weight-reducing effect (Henning et al., 2018). WIP treatment enhanced intestinal butyric acid levels, improved intestinal mucosal integrity, and regulated PPAR-γ pathway in the intestine (Sun et al., 2019). Dingxin Recipe IV (DXR) intervention promoted acetate, butyrate, propionate production, inhibited LXR-α/SREBP1 pathway and improved lipid metabolism (Zhang et al., 2021c). The mechanism through which GLP intervention ameliorated LMD may be indirectly engaged in regulating lipid metabolism by promoting the production of acetate and butyrate, activating the GPR43 receptor and modulating the intestinal barrier and inflammatory response (Sang et al., 2021).

### 3.4 TCM regulates BAs metabolism to improve LMD

Studies have shown that GXNT (Yang Q. et al., 2022) and Quercetin (Nie et al., 2019) can reduce serum or intestinal BAs levels. HYQT (Sui et al., 2021b), HZRG (Li C. et al., 2022), Proanthocyanidin (Fu et al., 2013), Theabrownins (Huang et al., 2019), *Radix scutellariae* water extract (Zhao et al., 2021), ZB (You, 2016), GbE (Wang Y. et al., 2022), QGE (Li et al., 2020) and Pae (He, 2021) promoted the conversion of cholesterol to BA and significantly increased the excretion of BA in feces. In-depth studies have revealed that the hypolipidemic effects of TCM are closely related to the modulation of BA synthesis and transport pathways. Among them, HYQT can increase BA synthesis-related genes expression, upregulate hepatic CYP7A1 expression, promote BA biosynthesis, and reduce serum lipid levels (Sui et al., 2021b). XXD may be achieved hypolipidemic effects by upregulating CYP8B1 expression (Lei, 2020), and Baicalein exhibited the same effects (Li P. et al., 2022). HZRG aimed to reduce serum TC and TG levels mainly by decreasing the expression of BA transporter-related genes ASBT and OSTβ mRNA and reducing the reabsorption of harmful BA such as LCA, DCA and HCA (Li C. et al., 2022). QGE promoted BA transport in the liver by increasing BSEP expression and hepatic TGR5 receptor expression to attenuate the inflammatory response and regulate lipid metabolism in the liver (Li et al., 2020). RC alkaloids reduced lipid and TBA levels involving multiple BA receptor pathways, and were associated with reduced ASBT expression and elevated CYP7A1 and TGR5 expression (He et al., 2016). NXT decreased BSH enzyme activity, modulated the BA profile in the intestine, and reduced lipid deposition, but the exact mechanism has not been elucidated (Lu et al., 2022). It was found that treatment with THF (Yang L. et al., 2022), WESB (Zhao et al., 2021), HQF (Sui et al., 2021b), TB (Huang et al., 2019), Pae (He, 2021), Naringin (Wang F. et al., 2020) and *Penthorum chinense Pursh.* (PCP) extract (Li X. et al., 2022) decreased the abundance of intestinal flora that produces BSH and BSH enzyme activity, leading to a weakened hydrolysis of BAs, increased the level of FXR antagonist T-βMCA, decreased FXR agonist CDCA and LCA levels, inhibited FXR/FGF15 axis in the intestine, activated CYP7A1 and CYP7B1 expression in the liver, and promoted hepatic BA synthesis, thereby reducing serum and hepatic TC levels. In addition, RSV enhanced BSH enzyme activity *via* boosting the number of BSH-producing bacteria, including *Bifidobacterium* and *Lactobacillus*, to promote BA catabolism and fecal excretion in the intestine, downregulated OSTα, OSTβ expression to reduce BA reabsorption transport, and reduced CDCA content to suppress enterohepatic FXR/FGF15 axis in order to promote hepatic BA synthesis (Chen et al., 2016).

### 3.5 TCM adjusts TMAO production to improve LMD

It was found that serum lipid and TMAO levels were significantly decreased in HFD-induced AS model animals after treatment with Ginkgolide B (GB) (Lv et al., 2021), *Eucommia ulmoides* extract (Sun, 2020), HYQT (Sui et al., 2021a) and AOB (Zhu, 2021). The possible mechanism is to reduce TMA production, decrease hepatic FMO3 expression and inhibit the oxidation of TMA to TMAO by adjusting intestinal flora, which is a process consistent with the study of Xiangsha Liujunzi Decoction (XSLJZ) for the treatment of hyperlipidemia model rats (Wang J. et al., 2022). BBA reduces TMAO biosynthesis by decreasing the abundance of TMAO-related enzymes, such as carnitine oxygenase (CntA), choline-trimethylamine lyase (CutC), FMO and betaine reductase, effectively reducing serum TMAO levels (Wu et al., 2020; Li X. et al., 2021; Ma et al., 2022). The content of TMAO in the blood was shown to be associated to the severity of AS. Treatment with Tongmai Zhuyu decoction (TMZY) (Ji et al., 2020) or Tanyutongzhi Formula (TYTZ) (Wang, 2016) led to the reduction of TMAO levels as well as the decrease of atherosclerotic plaque area in AS model animals, and its anti-AS effect may be connected with TMAO reduction and the reduction of endothelial damage and inflammation levels. Another study showed that GXNT treatment was effective in reducing the production of TMAO, a TMA metabolite, lowering ox-LDL levels and inhibiting macrophage foaminess, while reducing NF-κB expression in AS plaques and alleviating the inflammatory response, thus preventing and preventing the formation of AS, which was correlated with reduced *Escherichia* abundance (Yang Q. et al., 2022). Furthermore, Jianpi Huazhuo Tiaozhi Granule (JPHZTZ) (Huang Q., 2021) and RSV (Chen et al., 2016) also exerted significant efficacy in anti-AS by a mechanism that may be achieved by inhibiting the enterohepatic FXR/FGF15 pathway, upregulating hepatic CYP7A1 expression, promoting BA biosynthesis, reducing TMAO production, and maintaining cholesterol metabolic homeostasis.

## 4 Discussion

Lipid metabolism, which is an essential component of an organism’s fundamental metabolism, and LMD is linked inextricably to numerous illnesses and their consequences. With the rapid development of sequencing technology, significant advancements in our study of intestinal flora have been achieved, and it is also clear that lipid metabolism is closely related to intestinal flora. When LMD occur, the structure and composition of intestinal flora are abnormal, while dysbiosis of intestinal flora can further aggravate LMD. Therefore, the relationship between intestinal flora metabolites and LMD (hyperlipidemia, obesity, NAFLD and AS) can provide new perspectives for understanding LMD, and promote the development of research and treatment methods of metabolism disorders.

In this review, we systematically analyzed the previous related literature and found that intestinal flora and its metabolites may be major targets of TCM for treating LMD (Tables 1, Supplementary Table S1). The composition of Chinese herbal formulas and proprietary Chinese medicines is shown in the Supplementary Table S2. TCM can reshape the composition and structure of intestinal flora by enhancing the abundance of beneficial intestinal bacteria, for example, increasing the abundance of *Akkermansia* to repair intestinal barrier function (Park et al., 2019); upregulating *Prevotella*, a bacteria that produces SCFAs, to improve lipid metabolism of the host and alleviate inflammatory responses (Tong et al., 2020); promoting the growth of *Lactobacillus* and *Bifidobacterium*, and enhancing BSH enzyme activity to promote BA catabolism and fecal excretion in the intestine to accelerate cholesterol excretion (Yang L. et al., 2022). At the same time, TCM can also reduce the abundance of harmful bacteria by inhibiting the growth of pathogenic bacteria such as *Escherichiacoli* and *Enterococcus*, down-regulating the number of *Desulfovibrio*, bacteria that produces LPS, alleviating low chronic inflammatory response and metabolic endotoxemia in the intestine, and maintaining intestinal microecological health (Zhang et al., 2018; Kou et al., 2022). Notably, TCM also reduces the risk of abnormal lipid metabolism diseases by lowering the *F/B* ratio, reducing fat accumulation in the body and accelerating fat metabolism. For example, Luteolin modulated intestinal flora (decreased *F/B* ratio, elevated *Bifidobacterium*, *Lactobacillus*) against liver fatty lesions in rats (Liu, 2022), while BBR successful in boosting *Akkermansia* abundance, it also enhanced intestinal tight junction protein expression and colonic mucus layer thickness, reduced HFD-induced metabolic endotoxemia and decreased expression of pro-inflammatory factors and chemokines (Zhu et al., 2018). After SLBZ supplementation treatment, the abundance of *Desulfovibrio* was reduced in NAFLD rats, and LPS production as well as the secretion of inflammatory factors were inhibited, resulting in improvement of LMD (Hong, 2021). NXT reduced BSH enzyme activity in hyperlipidemic rats and modulated the BA profile in the intestine to reduce lipid levels in serum and liver (Lu et al., 2022). RLPs increased the relative abundance of *Prevotella*, promoted the production of SCFAs such as Propionic acid and butyric acid, and reduced lipid levels in NAFLD mice (Zhang X. J., 2020). In addition, TCM can also modulate intestinal flora metabolites to play a role in correcting LMD, including LPS, SCFAs, BAs and TMAO. Among them, LPS and SCFAs-mediated inflammatory pathways are key aspects of herbal agents for lipid lowering. BAs have a vital part in maintaining lipid metabolism homeostasis, involving lipid synthesis, transport, and excretion. TMAO is positively correlated with visceral obesity index and is not only involved in the biosynthesis of BAs, but also serves as a biomarker for AS (Barrea et al., 2018). Given its multi-component and multi-target of action characteristics, TCM often also affects MAPK or TLR4/NF-κB pathway-mediated chronic inflammatory responses and FXR/FGF15 pathway-mediated metabolism of BAs to regulate lipid metabolism. For instance, Quercetin reduced LPS translocation, promoted SCFA production, increased intestinal Occludin, Claudin-1 expression, repaired intestinal barrier function, and inhibited TLR4 pathway to improve inflammation levels by regulating NAFLD intestinal flora (Porras et al., 2017). Myricetin significantly reduced LPS-induced metabolic endotoxemia and systemic inflammation, corrected dyslipidemia and restored liver function in NAFLD rats by regulating the TLR4/NF-κB signaling pathway (Sun et al., 2021). After treatment with BTP, it can effectively increase the level of SCFAs in obese mice, and at the same time activate AMPK signaling pathway to increase energy consumption for weight loss (Henning et al., 2018). QGE promoted BA transport in the liver by increasing BSEP expression, in addition to upregulating hepatic TGR5 receptor expression, attenuating hepatic inflammatory response and regulating lipid metabolism in NAFLD mice (Li et al., 2020). RSV regulated lipid levels mainly by regulating BA metabolism, which not only promoted the catabolism of BA in the intestine and fecal excretion, but also downregulated OSTα, OSTβ expression and reduced BA reabsorption transport (Chen et al., 2016). HYQT may reduce TMA production, decrease hepatic FMO3 expression and inhibit TMAO synthesis by altering the structure of the intestinal flora; meanwhile, it can inhibit the intestinal and hepatic FXR/FGF15 axis, promote the synthesis and excretion of BAs and maintain the balance of cholesterol metabolism, and finally achieve the purpose of preventing and treating AS (Sui et al., 2021a; Sui et al., 2021b). It can be seen that TCM and its effective ingredients can improve the metabolic indexes of blood lipids directly or indirectly by acting on intestinal flora and its metabolites, thus playing a comprehensive role in preventing and treating LMD.

**TABLE 1:** Therapeutic effects of Chinese herbal formulas and proprietary Chinese medicines on LMD.

Intervention	Model	Outcome	Changes in intestinal flora	Potential mechanism	References
Alisma orientalis Beverage (AOB)	AS ApoE^−/−^ mice	1. Serum TC, TG, LDL-C↓	*Firmicutes, Lactobacillus↓*; *Bifidobacterium*, *Actinobacteria↑*	1. Serum TMAO, FMO3↓;	Zhu et al. (2019)
				2. Inflammatory: TNF-α, IL-1β, L-6, IL-10, IL-17↓	
Biejia Jian Wan (BJJW)	NAFLD	1. Serum ALT, AST, ALP↓	*Bacteroidetes↓*; *Lactobacillus↑*	1. Intestinal barrier: Occludin mRNA↑	Qiu et al. (2017)
	SD rats				
Cangju Qinggan Formula (CJQG)	NAFLD	1. Serum TC, TG, LDL-C, ALT, AST↓, HLD-C↑;	*Firmicutes↓*; *Verrucomicrobia*, *Bacteroidetes↑*	—	Ling et al. (2022)
	SD rats	2. Hepatocyte fat vacuoles, lipid droplet deposition↓			
Chaihu Shugan San (CHSG)	NAFLD patients	1. BMI↓;	*Enterobacter, Enterococcus↓*; *Bifidobacterium*, *Lactobacillus↑*	1. Inflammatory: TNF-α, L-6, IL-1β, TLR4, CAP, LSM↓	Xie et al. (2021)
		2. Serum TC, LDL-C, ALT, AST↓, HDL-C↑			
Danggui Shaoyao San (DGSY)	NAFLD	1. Serum TC, TG, ALT, AST↓, Liver TC, TG, FFA↓;	*F/B* ratio, *Firmicutes↓*; *Bacteroidetes↑*	-	Yu et al. (2021)
	SD rats	2. Hepatocyte fat vacuoles↓			
Dingxin Recipe IV (DXR)	AS ApoE^−/−^ mice	1. Serum TC, TG, LDL-C, HDL-C↑	*Firmicutes*, *Erysipelotrichaceae*, *Ileibacterium*, *Allobaculum↓*; *Bacteroides*, *Muribaculaceae*, *Ruminococcaceae↑*	1. SCFAs: Acetate, butyrate, propionate, valerate, choline↑;	Zhang et al. (2021)
				2. BCAAs: alanine, glutamate succinate↑;	
				3. Signaling pathway: LXR-α/SREBP1↓	
Erchen Decoction (ECD)	NAFLD	1. BW↓;	*Firmicutes, Proteobacteria↓*; *Bacteroidetes*, *Cyanobacteria*, *Verrucomicrobia, Akkermansia, Alloprevotella*, *B. fragills*, *Clostridium XIVa*, *Coprococcus*, *Prevotella*, *Ruminococcus*, *Roseburia↑*	1. Inflammatory: Serum LPS, hepatic TLR-4, IL-1β, TNF-α, NF-κB↓;	Liu et al. (2021)
	C57BL/6J mice	2. Serum TC, TG, FFA, ALT, AST↓;		2. Intestinal barrier: ZO-1, Occludin, Claudin-3↑;	
		2. Liver lipid deposition↓		3. Fecal SCFAs↑;	
				4. Signaling pathway: LPS/TLR-4↓	
Gegen Qinlian Decoction (GGQL)	Hyperlipidemia	1. BW, Lee index↓;	*Tyzzerella*, *Anaerotruncus↓*	-	Jiang et al. (2021)
	SD rats	2. Serum TC, TG↓			
Gegen Qinlian Decoction (GGQL)	NAFLD	1. Serum TG, TC, LDL-C↓;	*Firmicutes*, *Proteobacteria, Desulfovibrio*, *Butyricicoccus*, *Ruminococcaceae↓*; *Bacteroidetes*, *Bacteroides↑*	1. Inflammatory: LPS↓;	Liu et al. (2021)
	C57BL/6 mice	2. Hepatic steatosis↓		2. Intestinal barrier: ZO-1, Occludin, Claudin-1, CX3CR1↑	
Guizhi Tang (GZT)	AS ApoE^−/−^ mice	1. Atherosclerotic plaque area↓	*Firmicutes*, *Proteobacteria*, *F/B* ratio*↓*, *Bacteroidetes*, *Verrucomicrobia↑*	1. Inflammatory: TLR4, CD36↓, Ly6C^++^, Ly6C^−^↑	Yuan et al. (2021)
Huanglian Jiedu Decoction (HLJD)	AS ApoE^−/−^ mice	1. Hepatic steatosis, Atherosclerotic plaque area↓	*Firmicutes*, *Proteobacteria*, *F/B* ratio*↓*, *Bacteroidetes*, *Verrucomicrobia↑*	1. Serum and liver MAO↓	Jiang et al. (2021)
Huangqin Decoction (HQD)	NAFLD	1. BW↓;	*Actinobacteria*, *Lactobacillus*, *Bifidobacterium↓*; *unclassified-f-Lachnospiraceae*, *Blautia*, *Eubacterium*, *coprostanoligenes group*, *Ruminococcus↑*	1. Signaling pathway: Amino acid metabolism, carbohydrate metabolism	Yang et al. (2022)
	SD rats	2. Serum TC, TG, LDL-C, ALT, AST↓, HDL-C↑;			
		3. Hepatic steatosis↓			
Huayu Qutan Formula (HYQT)	AS ApoE^−/−^ mice	1. Hepatocyte fat vacuoles, lipid droplet deposition↓	*g_Eubacterium_xylanophilum_group*, *g _Lachnospiraceae_UCG-006*, *g_Rikenellaceae_RC9_gut_group*, *Roseburia↓*, *Citrobacter*, *Bacteroides*, *Shigella*, *Parabacteroides*, *Staphylococcus↑*	1. Serum TMA, TMAO↓, FMO3 mRNA and protein expression↓	Sui et al. (2021a)
Huayu Qutan Formula (HYQT)	AS ApoE^−/−^ mice	1. Serum TC, TG, LDL-C↓	*Lactobacillus↓*, *Bacteroides*, *Clostridium↑*	1. BAs: Fecal BAs↑, ileal BAs↓, ileal CA, DCA, TCA, TDCA↓;	Sui et al. (2021b)
				2. Signaling pathway: ileal FXR, FGF-15 mRNA↓, Liver CYP7A1 mRNA↑	
Huayu Qutan Formula (HYQT)	AS ApoE^−/−^ mice	-	*Bacteroidaceae*, *Staphylococcaceae*, *Bacillales*, *Enterococcaceae*, *Lactobacillales*, *Bacilli*, *Enterobacteriaceae*, *Enterobacteriales*, *Gammaproteobacteria↑*	1. Inflammatory: LPS↓;	Zheng et al. (2022)
				2. Intestinal barrier: ZO-1, Occludin, Claudin1 mRNA and protein expression↑	
Huazhi-Rougan Formula (HZRG)	NAFLD C57BL/6J mice	1. Liver TG, serum ALT and AST levels↓	*Lactobacillaceae*, *Bifidobacteriaceae*, *Clostruduaceae*, *Chostridiales VadinBB60*, *Corynebacteriaceae*, *Solanales*, *Propionibacteriaceae*, *Micrococcaceae*, and *Satphylococcaceae↑*	1. Inflammatory: serum TNF-α↓, liver p-P65, F4/80↓, liver TNF-α, IL-1β mRNA↓;	Li et al. (2022)
				2. BAs: Fecal LCA, HCA, βDCA↑; serum LCA, DCA, HCA↓;	
				3. BA-related receptor e expression: ASBT, OSTβ mRNA↓;	
				4. Intestinal barrier: ZO-1, Occludin, Claudin-2↑;	
				5. Signaling pathway: BA biosynthesis; BA transporters, TLR4/NF-kB p65 pathway	
Jian Pi Tiao Gan Yin (JPTGY)	Obesity C57BL/6 mice	1. Serum TC, TG, LDL-C↓, HDL-C↑	*Proteobacteria*, *F/B ratio↓*; *Lachnospiraceae NK4A136 group*, *Oscillibacter*, *Turicibacter*, *Clostridium sensu stricto 1*, *Clostridiaceae 1*, *Erysipelotrichia*, *Erysipelotrichales*, *Erysipelotrichaceae*, *Parvibacter*, *gut_metagenome*, *Intestinimonas↑*	1. Signaling pathway: linoleic acid metabolism paths, alpha-linolenic acid metabolism paths, glycerophospholipid metabolism paths, arachidonic acid metabolism paths, pyrimidine metabolism paths	Dong et al. (2022)
Jiangzhi Granules (JZG)	NAFLD C57BL/6 mice	1. BW↓;	*F/B ratio*, *Desulfovibrionaceae↓*; *S24_7*, *Lachnospiraceae*, *Bifidobacteriaceae↑*	1. Inflammatory: Hepatic CD14, TLR2, TLR4, NLRC4, MCP-1↓	Wang et al. (2021)
		2. Serum TC, TG, FFA, ALT↓;		2. Oxidative stress: SOD↑, MDA↓;	
		2. Hepatic steatosis, liver lipid deposition↓		3. Intestinal barrier: ZO-1, Occludin, Muc5↑;	
				4. SCFAs: Total SCFAs↑;	
				5. Signaling pathway: BA secretion, PPAR↑	
Jiangzhi Ligan Decoction (JZLG)	NAFLD	1. BW, liver weight, liver index↓	*Escherichiacoli↓*, *Lactobacillus↑*	—	Tang et al. (2016)
	SD rats				
Jianpi Huazhuo Tiaozhi Granule (JPHZTZ)	AS ApoE^−/−^ mice	1. Atherosclerotic plaques area↓;	*Turicibacter*, *Desulfovibrio*, *Alistipes↓*	1. Serum TMAO↓;	Huang et al. (2019)
		2. Serum TC, TG, LDL-C↓		2. Inflammatory: TNF-α, L-6↓;	
				3. Signaling pathway: FXR/FGF15 Axis↓, CYP7A1↑	
Liqi Huatan Quyu Decoction (LQHTQY)	Hyperlipidemia	1. Serum TC, TG, LDL-C↓;	*Firmicutes↓*; *Bacteroidia*, *Actinobacteria*, *Clostridia*, *Paraprevotella↑*	—	Ding et al. (2019)
	Golden hamster	2. Hepatic steatosis↓			
Modfied Yinchen Wuling San (MYCWL)	NAFLD patients	1. Serum ALT, AST↓	*Escherichiacoli*, *Staphylococcus↓*; *Bifidobacterium*, *Lactobacillus*, *Bacteroidetes↑*	—	Xu et al. (2019)
Modified Xiongdan yinchen Granules (MXYG)	NAFLD	1. Serum TC, TG, LDL-C, ALT, AST↓, HDL-C↑	*Verrucomicrobia*, *Phascolarctobacterium*, *Brautella↓*; *Clostridium*, *Lactobacillus*, *Bacteroides*, *Lachnospira↑*	1. Intestinal barrier: Intestinal permeability, FITC-D↓	Wu et al. (2021)
	SD rats				
Qiang Gan Formula extract (QGE)	NAFLD	1. Liver weight↓;	*Bacteroides*, *Clostridia↑*	1. Inflammatory: IL-1β, TNF-α mRNA↓;	Li et al. (2020)
	C57BL/6 mice	2. Serum TC, AST, ALT↓		2. BAs: Serum and liver BA↓, Serum TDCA, TLCA↓, fecal α+ω MCA, LCA↑;	
				3. Signaling pathway: TLR4/MyD88/NF-κB↓, TGR5, BSEP, MRP2 mRNA↑	
Qinggan Qushi Huoxie Prescription (QGQSHX)	NAFLD	—	*Escherichiacoli*, *Enterococcus↓*; *Bifidobacterium*, *Lactobacillus↑*	—	Zhang et al. (2022)
	SD rats				
Qinghua Fang (QHF)	NAFLD	1. BW↓;	*Clostridium glycyrrhizinilyticum↓; Flintibacter butyricus, Blautia↑*	1. Inflammatory: IL-6, IL-8, TNF-α, IL-17↓	Wang et al. (2021)
	Wistar rats	2. Serum TG, AST, ALT↓;			
		3. Liver lipid deposition↓			
Qingxin Jieyu Granule (QXJY)	AS ApoE^−/−^ mice	1. Atherosclerotic plaques area↓;	*Akkermansia, Lachnospiraceae, Blautia, Clostridium↑*	1. Inflammatory: IL-1β↓;	Wang (2019)
		2. Serum TC, TG, LDL-C↓, HDL-C↑		2. SCFAs: Butyric acid, ISO-VA, 4-MEVA in urine↑	
Jieyu Qutan Huazhuo Prescription (JYQTHZ)	Hyperlipidemia	1. Serum TC, TG, LDL-C↓, HDL-C↑;	*Erysipelotrichales↓*; *Bacteroidia*, *Ruminococcaceae*, *Bacteroides S24-7*, *Rumencoccus UCG-005↑*	—	Li et al. (2021)
	Wistsr rats	2. Hepatic steatosis↓, Ileal structural integrity↑			
Jieyu Qutan Huazhuo Prescription (JYQTHZ)	Hyperlipidemia	1. BW↓;	*F/B* ratio*↓*; *Bacteroidia↑*	—	Li et al. (2022)
	Wistsr rats	2. Serum TC, TG, LDL-C↓, HDL-C↑			
Quyu Huatan Tongmai Prescription (QYHTTM)	Hyperlipidemia	1. Serum TC, TG, LDL-C/HDL-C↓	*Firmicutes, F/B* ratio, *Coriobacteriaceae_UCG_002↓*; *Proteobacteria*, *Deferribacteres*, *Bacteroidetes*, *Bacteroidaceae*, *Porphyromonadaceae*, *Rikenellaceae*, *Clostridiales_vadinBB60_grou*, *Family_ⅩⅢ*, *Lachnospiraceae*, *Desulfovibrionaceae*, *Bacteroides*, *Barnesiella*, *Odoribacter*, *Rikenellaceae_RC9_gut_group*, *Family_ⅩⅢ_AD3011_group*, *Acetatifactor*, *Coprococcus_1*, *Lachnospiraceae_FCS020_group*, *Roseburia*, *Oscillibacter*, *Ruminococcaceae_UCG_005*, *Desulfovibrio*, *Anaerotruncus*, *Ruminiclostridium_9*, *Ruminococcaceae_NK4A214_group↑*	—	Miao et al. (2022)
	Golden hamster				
Shenerjiangzhi Formulation (SEJZ)	Hyperlipidemia	1. BW, serum TC, TG, LDL-C↓, serum HDL-C↑	*Firmicutes/Bacteroidetes (F/B) ratio*, *Ruminococcus, and Oscillospira↓*; *Akkermansia*, *Allobaculum*, *Prevotella*, *Lactobacillus*, *Roseburia*, *Phascolarctobacterium*, *Blautia*, *Coprococcus↑*	1. Signaling pathway: African trypanosomiasis pathway↑	Zhang et al. (2022)
	SD rats				
Shenling baizhu Powder (SLBZ)	NAFLD	1. BW↓;	*Verrucomicrobia*, *Blautia*, *Roseburia*, *Phascolarctobacterium*, *Desulfovibrio↓*; *Actinobacteria*, *Cyanobacteria*, *Bifidobacterium*, *Anaerostipes*, *Akkermansia↑*	1. Inflammatory: LPS, IL-1β, TNF-α, TLR-4↓;	Zhang et al. (2018)
	SD rats	2. Serum TC, AST, ALT↓, Hepatic TC, TG↓;		2. Signaling pathway: TLR4/MyD88/TRAF6↓, NLRP3↓, GPR43↑	
		2. Hepatic steatosis↓			
Shenlingbaizhu Powder (SLBZ)	NAFLD	1. Serum ALT, AST, TC, TG, LDL-C, HDL-C, TBIL↓	*Helicobacter*, *Lachnospiraceae UCG-008↓*; *Lachnospiraceae NK4A136 group*, *Dubosiella*, *Bifidobacterium*, *Lactobacillus↑*	1. SCFAs: propionic, butyric acid↑	Hong (2021)
	C57BL/6 mice				
Si Miao Formula (SMF)	NAFLD	1. BW, liver weight, eWAT weight↓;	*Firmicutes↓*; *Verrucomicrobia*, *Proteobacteria*, *Akkermansia*, *Bifidobacterium*, *Faecalibaculum↑*	1. Inflammatory: IL-1β, NLRP3↓	Han et al. (2021)
	C57BL/6 mice	2. Serum TC, LDL-C↓, Liver TG↓;			
		3. Hepatic steatosis↓			
Si Ni San (SNS)	NAFLD	1. BW, Liver index↓;	*Oscillospira*, *Ruminococcaceae*, *Clostridiales*, *Clostridia↑*	1. Inflammatory: LPS, TNF-α↓	Zhu et al. (2019)
	C57BL/6 mice	2. Serum ALT↓, Liver TC↓			
Tanyutongzhi Formula (TYTZ)	AS LDLr^−/−^ mice	1. Atherosclerotic plaques area↓;	—	1. Serum TMAO, sVCAM-1↓, NO↑	Wang et al. (2018)
		2. Serum TC, TG, LDL-C↓, HDL-C↑			
Tian Huang Formula (THF)	Hyperlipidemia C57BL/6J mice	1. Serum and liver TC, TG↓	*Proteobacteria*, *Actinobacteria*, *Escherichia*, *Enterobacteriaceae*, *Ruminococcaceae*, *Lachnospiraceae↓*, *Akkermansia* and *Bacteroides↑*	1. BAs: DCA, CDCA, LCA↓, T-βMCA↑;	Yang et al. (2022)
				2. BSH↓;	
				3. Signaling pathway: FXR/FGF15, FXR/SHP↓, CYP7A1↑	
Tian Huang Formula (THF)	NAFLD	1. Serum TC, TG, LDL-C↓, Liver AST, ALT↓	*Lactobacillus↑*	1. Oxidative stress: GSH, SOD↑, MDA↓; 2. Signaling pathway: Nrf2↓	Pang (2021)
	C57BL/6J mice				
Tongmai Zhuyu Decoction (TMZY)	AS Wistar rats	1. BW↓;	*Blautia↓*, *Allobaculum↑*	1. Inflammatory: IL-4↑, IFN-γ, hs-CRP, IFN-γ/IL-4 ratio↓;	Ji et al. (2020)
		2. Serum TC, TG, LDL-C↓, HDL-C↑;		2. TMAO↓;	
		3. Platelet aggregation↓;		3. Immunoregulatory factors: TGF-β, FOXP3↑, VCAM-1, HMGB-1↓	
		4. Atherosclerotic plaque area↓			
Xiangsha Liujunzi Decoction (XSLJZ)	Hyperlipidemia	1. Serum TC, TG, LDL-C↓, serum HDL-C↑;	—	1. Serum TMA, TMAO↓; 2. Signaling pathway: TMAO/PERK/FOXO1 signaling Pathway↓, FMO3, PERK, FOXO1, MTP, Apoc-Ⅲ, ApoB, VLDLr mRNA and protein expression↓	Wang et al. (2022)
	SD rats	2. Hepatocyte steatosis, fat vacuoles↓			
Xiaoyao San (XYS)	NAFLD	1. Serum ALT, TBIL↓, AKP, TBA↑	*Muribaculaceae↓*; *Faecalibaculum↑*	1. Inflammatory: TNF-α, IL-1β↓	Zhou et al. (2021)
	C57BL/6J mice				
Xiexin Decoction (XXD)	AS ApoE^−/−^ mice	1. BW↓;	*Desulfovibrio*, *Lachnospiraceae_NK4A136 group*, *Eubacterium_xylanophi lum group↑*	1. Inflammatory: TNF-α↓;	Chen et al. (2020)
		2. Atherosclerotic plaques area↓;		2. Intestinal barrier: Occludin mRNA↑;	
		3. Serum TC, LDL-C↓		3. BAs: UDCA, TCDCA↓;	
				4. BAs -related receptor e expression: CYP8B1, LXR mRNA↑	
Xiexin Decoction (XXD)	AS ApoE^−/−^ mice	1. Atherosclerotic plaques area↓;	*Erysipelotrichaceae*, *Coriobacteriaceae↓*; *Ruminococcaceae↑*	1. Inflammatory: TLR4 mRNA, IL-12↓	Lei (2020)
		2. Serum TC, LDL-C↓			
Yinchenhao Decoction	NAFLD SD rats	—	*Firmicutes*, *Actinobacteria*, *Proteobacteria*, *Aggregatibacter*, *Clostridium*, *Prevotella*, *Staphylococcus*, *Streptococcus↓*; *Bacteroidetes*, *Bifidobacterium*, *Christensenella*, *Desulforibrio*, *Dorea*, *Faecalibacterium*, *Oscillospira*, *Paraprevotella*, *Psychrobacter*, *Rothia*, *Ruminococcus*, *Sutterella↑*	1. Signaling pathway: Glycerophospholipid metabolism, purine metabolism, glutathione metabolism	Li et al. (2019)
Yunpi Huazhuo Granules (YPHZ)	NAFLD patients	1. Serum TC, TG, LDL-C↓, HDL-C↑	*Escherichiacoli*, *Enterococcus↓*; *Bifidobacterium*, *Lactobacillus↑*	—	Kou et al. (2022)
Zexie Tang (ZXT)	Hyperlipidemia	1. Serum TC, TG, LDL-C↓	*Firmicutes*, *Phascolarctobacterium*, *Morganella*, *Proteus*, *Providencia↓*; *Bacteroidetes*, *Bifidobacterium↑*	—	Xu et al. (2017)
	SD rats				
Compound Danshen Dripping Pills	NAFLD	1. Liver lipid deposition↓	*F/B ratio↓*; *norank-f-Lachnospiraceae↑*	1. Inflammatory: LPS, TNF-α, IL-6, IL-1β↓;	Zhang et al. (2020)
	KKAy mice			2. SCFAs: Hexanoic acid, isohexanoic acid, butyric acid↑;	
				3. Signaling pathway: TLR4/MyD88/NF-κB↓	
Danlou Tablet (DLT)	AS ApoE^−/−^ mice	1. Serum TC, TG, LDL-C↓;	*Bacteroidetes*, *F/B* ratio, *Desulfovibrio*, *Lachnoclostridium*, *Alistipes*, *Lactococcus↑*	1. Inflammatory: LPS↓, TLR4, TNF-α, ICAM-1, IL-1β mRNA↓;	Sun et al. (2020)
		2. Hepatic steatosis, Atherosclerotic plaque area↓		2. SCFAs: Butyric acid↑	
Dengzhan Shengmai Capsules (DZSM)	NAFLD Golden hamsters	1. Serum CHO, TG, LDL-C, ALT↓, Liver TG↓;	*Firmicutes*, *Desulfobacterota, Lachnospiraceae*, *Desulfovibrionaceae*, *Oscillospiraceae*, *Desulfovibrio*, *Oscillibacter↓*; *Bacteroidota*, *Actinobacteriota*, *Muribaculaceae*, *Lactobacillaceae*, *Bifidobacteriaceae*, *Lactobacillus*, *Bifidobacterium*, *Allobaculum↑*	1. SCFAs: Acetic acid, propionic acid, butyric acid↑;	Guo et al. (2022)
				2. Intestinal barrier: ZO-1, Occludin↑;	
		2. Hepatocyte fat vacuoles, lipid droplet deposition↓		3. Inflammatory: LPS, TLR4, Ikkβ↓;	
				4. Signaling pathway: TLR4/NF-κB↓	
Guanxinning Tablet (GXNT)	AS Tibetan minipigs	1. Serum TC, LDL-C↓, HDL-C↑;	*Proteobacteria*, *Enterobacteriaceae*, *Escherichia↓*; *Prevotellaceae*, *Prevotella↑*	1. Inflammatory: ox-LDL, CRP, TNF-α, IL-1β, NF-κB, MMP-9↓;	Yang et al. (2022)
				2. Oxidative stress: SOD↑, MDA↓;	
				3. SCFAs: Fecal propionic acid, butyric acid↑;	
		2. Lipid deposition, atherosclerotic plaques area*↓*		4. BAs: CDCA↓;	
				5. Fecal TMA, TMAO↓;	
				6. Signaling pathway: BA metabolism, acetate kinase production, lipopolysaccharide biosynthesis↓, SCFA transport, butyrate kinase production, propionate kinase production↑	
Hugan Qingzhi Tablet (HGQZ)	NAFLD	1. Serum TC, TG, LDL-C↓, HDL-C↑	*F/B ratio*, *Staphylococcus*, *Streptococcus*, *Holdemanella*, *Blautia↓*; *norank_f_Bacteroidales_S24_7_group*, *Ruminococcaceae*, *Bifidobacterium*, *Alistipes*, *Cronobacter*, *Anaeroplasma*, *Bilophila↑*	1. Inflammatory: LPS, TNF-α, IL-6, IL-1β↓	Tang et al. (2018)
	C57BL/6J mice				
Hugan Qingzhi Tablet (HGQZ)	NAFLD C57BL/6J mice	1. Serum TC, TG, LDL-C↓, HDL-C↑	*F/B ratio*, *Cronobacter*, *Streptococcus*, *Holdemanella*, *Blautia↓*; *norank_f_Bacteroidales_S24_7_group*, *Ruminococcaceae*, *Bifidobacterium*, *Alistipes*, *Anaeroplasma↑*	1. Inflammatory: LPS, TNF-α, IL-6, IL-1β↓;	Tang et al. (2018)
				2. Intestinal barrier: claudin-1, ZO-1↑;	
				3. SCFAs: Total SCFAs, Acetic, propionic, isobutyric, butyric, valeric, isovaleric, capric acid↑;	
				4. Signaling pathway: TLR4/MyD88/NF-κB↓	
Jiangan Jiangzhi Pill (JGJZ)	NAFLD SD rats	1. Serum TC, TG, ALT, AST↓; 2. Liver lipid deposition↓	*F/B ratio↓*	1. Inflammatory: IL-6, IL-1β, TNF-α↓	Zhao et al. (2022)
Naoxintong Capsule (NXT)	Hyperlipidemia SD rats	1. BW, serum TC, TG, LDL-C, liver TC, TG and liver index↓, serum HDL-C↑	*Firmicute*, *F/B ratio*, [*Ruminococcus*] *gauvreauii group*, *Collinsella*, *Romboutsia*, *Romboutsia ilealis↓*; *Bacteroidetes↑*	1. SCFAs: Fecal total SCFAs, acetic acid, Propionic acid, Butyric acid↑;	Lu et al. (2022)
				2. BSH↓;	
				3. BAs: Fecal unconjugated BAs, Total BAs↓;	
				4. Signaling pathway: pentose phosphate pathway↑	
Shanmei Capsule (SM)	Hyperlipidemia	1. Serum TC, TG, LDL-C↓	*F/B* ratio, *Lachnospiraceae NK4A136 group*, *Lachnospiraceae NK4B4 group↓*	-	Du et al. (2022)
	C57BL/6 mice				
Tongxinluo Capsules (TXL)	AS New Zealand white rabbits	1. Abdominal aorta plaques vulnerability index↓	*F/B* ratio, *Ruminococcus*, *albus↓*; *Bacteroides*, *Alistipes*, *Campylobacter*, *Rikenella*, *indistinctus*, *viscericola*, *nordii*, *subantarcticus↑*	1. Inflammatory: NLRP3, caspase-1, TNF-α, IL-1β, IL-18↓;	Qi et al. (2022)
				2. Other metabolite: trans-ferulic acid↑;	
				3. Signaling pathway: NLRP3 inflammatory pathway↓	
Zhibitai Capsule (ZBT)	NAFLD patients	1. Serum TC, TG, ALT, AST↓	*Escherichiacoli*, *Enterococcus*, *Staphylococcus↓*; *Bifidobacterium*, *Lactobacillus*, *Bacteroidetes↑*	1. Intestinal barrier: ET, DAO, PCT↓;	Pan et al. (2020)
				2. Inflammatory: TNF-α, IL-6↓	

The study of the interaction between TCM and intestinal microorganisms has an important prospect, and the in-depth study of the interaction between TCM and intestinal flora is conducive to the elucidation of the potential mechanisms of TCM in preventing and treating LMD, as well as to the enrichment of TCM theory. In recent years, a few reviews have been reported on the regulation of intestinal flora by TCM to improve metabolic diseases, but there are shortcomings such as few included studies and few diseases involved (Zhang H. Y. et al., 2021; Li Y. et al., 2021). Based on previous studies, this review enriches the number of included literature and adds the amount of LMD, including hyperlipidemia, obesity, NAFLD and AS. Also, we illustrate potential mechanisms of TCM for regulating intestinal flora to improve LMD by category, which helps to minimize the issue of biased reporting. In this review, we have conducted a full quality assessment of the included original literature, which includes the dosage, periodicity and quality control of the use of TCM prescriptions (Chinese herbal formulas and proprietary Chinese medicines) and natural medicine extracts in animal experiments and clinical trials (Tables 2–4). However, there are some limitations in the included studies. 1) The impact of TCM on LMD based on intestinal flora has gained attention in the scientific community, and the number of related clinical studies is growing. However, there are issues such as small sample sizes, poor quality of single studies, as well as few reports of large-scale clinical trials. 2) There were differences in the specific content of the control group between the included original literature, and although the experimental groups were Chinese herbal formulas or natural medicine extracts that could regulate intestinal flora, the specific groups were different, which was also a source of heterogeneity and may have affected the results of the review assessment. 3) The majority of the original literature that was considered did not mention any allocation concealment, blinding, or other concerns, which made the results less reliable. 4) Despite the extensive search, the currently included literature is mainly in English and Chinese, which may have regional bias and language bias. 5) The current research on the treatment of diseases related to LMD by TCM is mainly focused on obesity, hyperlipidemia and NAFLD, while there are fewer studies on diseases like AS, which may be related to the bioavailability of TCM and to the stage and severity of the disease. 6) The number of research on various intestinal metabolites is significantly skewed. Most studies of TCM against LMD have taken the chronic low levels of inflammation as their entry point, focusing mainly on the regulation of LPS levels and the levels of SCFAs in stool, while fewer studies have been conducted on BAs and TMAO, which are lipid metabolism-related risk factors. 7) Studies on intestinal flora are mostly correlational, while relatively few studies on causality are available. 8) Most of the research is focused on natural medicine extracts, but there is still considerable opportunity for research on proprietary Chinese medicines and Chinese herbal formulas. Therefore, Future research should focus on how the effective ingredients of TCM are bio-transformed by intestinal flora, and whether these biotransformation metabolites have synergistic or antagonistic effects on the treatment of LMD in TCM, which will help discover new favorable metabolites of intestinal flora. Furthermore, it is necessary to conduct more high-quality studies to verify the safety of TCM in modulating intestinal flora to improve LMD, so as to promote clinical application and provide new ways and references for the intervention targets of TCM in preventing and treating LMD.

**TABLE 2:** Summary of TCM prescriptions used in clinical trials.

Name	Dosage form	Research design	Grouping and number of people		Treatment method		Treatment	Quality control reported	References
			Treatment group	Control group	Treatment group	Control group	Duration		
Chaihu Shugan San (CHSG)	Granules	Randomized controlled trial	40	40	BYHWD (1 prescription/day, twice/day)	Placebo (1 prescription/day, twice/day)	12 weeks	Prepared according to China Pharmacopoeia	Xie et al. (2021)
Modfied Yinchen Wuling San (MYCWL)	Decoction	Randomized controlled trial	56	57	MYCWL (1 prescription/day, twice/day) + Polyene Phosphatidylcholine Capsules (3 times/day, 2 capsules for each time) + Live Combined *Bacillus Subtilis* and *Enterococcus* Faecium Enteric-coated Capsules (3 times/day, 2 capsules for each time)	Polyene Phosphatidylcholine Capsules (3 times/day, 2 capsules for each time) + Live Combined *Bacillus Subtilis* and *Enterococcus* Faecium Enteric-coated Capsules (3 times/day, 2 capsules for each time)	12 weeks	Prepared according to China Pharmacopoeia	Xu et al. (2019)
Yunpi Huazhuo Granules (YPHZ)	Granules	Randomized controlled trial	50	50	YQHZ (1 prescription/day) + Metformin (3 times/day, 250 mg for each time)	Metformin (3 times/day, 250 mg for each time)	2 months	Prepared according to China Pharmacopoeia	Kou et al. (2022)
Zhibitai Capsule (ZBT)	Capsule	Randomized controlled trial	38	38	ZBT (twice/day, 2 capsules for each time)	Polyene Phosphatidylcholine Capsules (3 times/day, 2 capsules for each time)	4 months	Purchased from Chengdu Diao Jiuhong Pharmaceutical Factory	Pan et al. (2020)

**TABLE 3:** Summary of TCM prescriptions used in animal experiments.

Name	Dosage form	Model	Treatment method and sample size			Treatment	Quality control reported	Chemical analysis	References
			Treatment group	Negative control group	Positive control group	Duration			
Alisma orientalis Beverage (AOB)	Decoction	AS mice induced by HFD (8 weeks)	AOB (3.25/6.5 g/kg/day) by gavage (*n* = 10/10)	The same volume of saline by gavage (*n* = 10)	Atorvastatin (1.3 mg/kg/day) by gavage (*n* = 10)	8 weeks	Prepared according to China Pharmacopoeia	HPLC	Zhu et al. (2019)
Biejia Jian Wan (BJJW)	Powder	NAFLD rats induced by HFD (7 weeks) + CCl4 olive oil solution (7th weeks)	BJJW (0.6/1.2/2.4 g/kg/day) by gavage (*n* = 10/10/10)	0.5% CMC-Na (0.01 mL/g/day) by gavage (*n* = 10)	Rosiglitazone (3 mg/kg) by gavage (*n* = 10)	4 weeks	Prepared according to China Pharmacopoeia	-	Qiu et al. (2017)
Cangju Qinggan Formula (CJQG)	Decoction	NAFLD rats induced by HFD (8 weeks)	CJQG (30.34/60.68/121.38 mg/kg/d) by gavage (*n* = 5/5/5)	The same volume of saline by gavage (*n* = 5)	Probiotic (107.32 mg/kg/d) by gavage (*n* = 5)	4 weeks	Prepared according to China Pharmacopoeia	-	Ling et al. (2022)
Danggui Shaoyao San (DGSY)	Granules	NAFLD rats induced by HFD (8 weeks)	DGSY (2.44/4.88/9.76 g/kg/d) by gavage (*n* = 10/10/10)	Distilled water (10 mL/kg/d) by gavage (*n* = 10)	Polyene Phosphatidylcholine Capsules (144 mg/kg/d) by gavage (*n* = 10)	8 weeks	Prepared according to China Pharmacopoeia	-	Yu et al. (2021)
Dingxin Recipe IV (DXR)	Decoction	AS mice induced by HFD (12 weeks)	DXR IV (0.45/0.9/1.8 g/kg/day) by gavage (*n* = 10/10/10)	The same volume of saline by gavage (*n* = 10)	-	12 weeks	Prepared according to China Pharmacopoeia	-	Zhang et al. (2021)
Gegen Qinlian Decoction (GGQL)	Decoction	Hyperlipidemia rats by HFD (5 weeks)	GGQL (1.65/4.95/14.85 mg/kg/day) by gavage (*n* = 6/6/6)	The same volume of saline by gavage (*n* = 6)	Simvastatin (10 mg/kg/day) by gavage (*n* = 6)	11 weeks	Prepared according to China Pharmacopoeia	HPLC	Jiang et al. (2021)
Gegen Qinlian Decoction (GGQL)	Decoction	NAFLD mice by HFD + DSS (12 weeks)	GGQL (8/16 mg/kg/day) by gavage (*n* = 14/14)	The same volume of saline by gavage (*n* = 14)	-	12 weeks	Prepared according to China Pharmacopoeia	HPLC	Liu et al. (2021)
Guizhi Tang (GZT)	Decoction	AS mice induced by HFD (4 weeks)	GZT (7.89 g/kg/day) by gavage (*n* = 10)	The same volume of double-distilled water by gavage (*n* = 10)	Atorvastatin (3.33 mg/kg/day) by gavage (*n* = 10)	4 weeks	Prepared according to China Pharmacopoeia	-	Yuan et al. (2021)
Huanglian Jiedu Decoction (HLJD)	Decoction	AS mice induced by HFD (4 weeks)	HLJD (5 g/kg/day) by gavage (*n* = 10)	The same volume of saline by gavage (*n* = 10)	Atorvastatin (3 mg/kg/day) by gavage (*n* = 10)	4/18 weeks	Prepared according to China Pharmacopoeia	-	Jiang et al. (2021)
Huangqin Decoction (HQD)	Decoction	NAFLD rats induced by HFD (9 weeks)	HQD (5/20 g/kg/day) by gavage (*n* = 6/6)	The same volume of saline by gavage (*n* = 6)	Polyene Phosphatidylcholine Capsules (8 mg/kg/d) by gavage (*n* = 6)	5 weeks	Prepared according to China Pharmacopoeia	UPLC-TQ-MS	Yang et al. (2022)
Huayu Qutan Formula (HYQT)	Decoction	AS mice induced by HFD (8 weeks)	HYQT (20 g/kg/day) by gavage (*n* = 8)	The same volume of saline by gavage (*n* = 8)	Simvastatin (2.275 mg/kg/day) by gavage (*n* = 8)	4 weeks	Prepared according to China Pharmacopoeia	UHPLC-MS/MS	Sui et al. (2021a)
Huayu Qutan Formula (HYQT)	Decoction	AS mice induced by HFD (8 weeks)	HYQT (20 g/kg/day) by gavage (*n* = 8)	The same volume of saline by gavage (*n* = 8)	Simvastatin (2.275 mg/kg/day) by gavage (*n* = 8)	4 weeks	Prepared according to China Pharmacopoeia	UHPLC-MS/MS	Sui et al. (2021b)
Huayu Qutan Formula (HYQT)	Decoction	AS mice induced by HFD (30 days)	HYQT (10/20/40 g/kg/day) by gavage (*n* = 10/10/10)	The same volume of saline by gavage (*n* = 10)	-	30 days	Prepared according to China Pharmacopoeia	-	Zheng et al. (2022)
Huazhi-Rougan Formula (HZRG)	Granules	NAFLD mice induced by HFD (4 weeks)	HZRG (3/6 g/kg/day) by gavage (*n* = 10/10)	The same volume of 0.5% CMC-Na by gavage (*n* = 10)	-	4 weeks	Prepared according to China Pharmacopoeia	UPLC-Q-TOF/MS	Li et al. (2022)
Jian Pi Tiao Gan Yin (JPTGY)	Decoction	Obesity mice induced by HFD (12 weeks)	JPTGY (12 g/kg/day) by gavage (*n* = 10)	The same volume of double-distilled water by gavage (*n* = 10)	-	12 weeks	Prepared according to China Pharmacopoeia	LC-MS	Dong et al. (2022)
Jiangzhi Granules (JZG)	Granules	NAFLD rats induced by HFD (16 weeks)	JZG (497/994 mg/kg/day) by gavage (*n* = 8/8)	The same volume of 0.5% CMC-Na by gavage (*n* = 8)	-	8 weeks	Prepared according to China Pharmacopoeia	UPLC-MS	Wang et al. (2021)
Jiangzhi Ligan Decoction (JZLG)	Decoction	NAFLD rats induced by HFD (12 weeks)	JZLG (4.6 g/kg/day) by gavage (*n* = 10)	The same volume of saline by gavage (*n* = 10)	-	4 weeks	Prepared according to China Pharmacopoeia	-	Tang et al. (2016)
Jianpi Huazhuo Tiaozhi Granule (JPHZTZ)	Decoction	AS mice induced by 1% choline (16 weeks)	JPHZTZ (9.49/18.98/37.96 g/kg/day) by gavage (*n* = 10/10/10)	The same volume of purified water by gavage (*n* = 10)	-	16 weeks	Prepared according to China Pharmacopoeia	-	Huang et al. (2019)
Liqi Huatan Quyu Decoction (LQHTQY)	Decoction	Hyperlipidemia hamsters induced by HFD (2 weeks)	LQHTQY (6.3/12.6/25.2 g/kg/day) by gavage (n = 8/8/8)	The same volume of saline by gavage (n = 8)	Fenofibrate (150 mg/kg/d) by gavage (*n* = 8)	6 weeks	Prepared according to China Pharmacopoeia	-	Ding et al. (2019)
Modified Xiongdan yinchen Granules (MXYG)	Granules	NAFLD rats induced by HFD (6 weeks)	MXYG (4.73/9.46/18.92 g/kg/day) by gavage (*n* = 10/10/10)	The same volume of saline by gavage (*n* = 10)	-	4 weeks	Prepared according to China Pharmacopoeia	-	Wu et al. (2021)
Qiang Gan Formula extract (QGE)	Decoction	NAFLD rats induced by HFD (4 weeks)	QGE (400 mg/kg/day) by gavage (*n* = 12)	The same volume of saline by gavage (*n* = 12)	vitamin E (120 mg/kg/day) by gavage (*n* = 12)	4 weeks	Prepared according to China Pharmacopoeia	HPLC	Li et al. (2020)
Qinggan Qushi Huoxie Prescription (QGQSHX)	Decoction	NAFLD rats induced by HFD (30 days)	QGQSHX (5.63/11.25/22.5 g/kg/day) by gavage (*n* = 15/15/15)	The same volume of distilled water by gavage (*n* = 15)	-	30 days	Prepared according to China Pharmacopoeia	-	Zhang et al. (2022)
Qinghua Fang (QHF)	Decoction	NAFLD rats induced by HFD (10 weeks)	QHF (0.2/0.4/0.8 g/kg/day) by gavage (*n* = 10/10/10)	The same volume of distilled water by gavage (*n* = 10)	-	10 weeks	Prepared according to China Pharmacopoeia	-	Wang et al. (2021)
Qingxin Jieyu Granule (QXJY)	Granules	AS mice induced by HFD (12 weeks)	QXJY (1.52/4.55 g/kg/day) by gavage (*n* = 10/10)	The same volume of saline by gavage (*n* = 10)	-	12 weeks	Prepared according to China Pharmacopoeia	LC-MS	Wang et al. (2018)
Jieyu Qutan Huazhuo Prescription (JYQTHZ)	Decoction	Hyperlipidemia rats induced by HFD (12 weeks)	JYQTHZ (0.4/0.8/1.2 g/kg/day) by gavage (*n* = 10/10/10)	The same volume of distilled water by gavage (*n* = 10)	Atorvastatin (2 mg/kg/day) by gavage (*n* = 10)	8 weeks	Prepared according to China Pharmacopoeia	-	Li et al. (2021)
Jieyu Qutan Huazhuo Prescription (JYQTHZ)	Decoction	Hyperlipidemia rats induced by HFD (12 weeks)	JYQTHZ (0.4/0.8/1.2 g/kg/day) by gavage (*n* = 10/10/10)	The same volume of distilled water by gavage (*n* = 10)	Atorvastatin (2 mg/kg/day) by gavage (*n* = 10)	8 weeks	Prepared according to China Pharmacopoeia	-	Li et al. (2022)
Quyu Huatan Tongmai Prescription (QYHTTM)	Decoction	Hyperlipidemia hamsters induced by HFD (4 weeks)	QYHTTM (1.33 g/kg/day) by gavage (*n* = 8)	The same volume of distilled water by gavage (*n* = 8)	-	6 weeks	Prepared according to China Pharmacopoeia	-	Miao et al. (2022)
Shenerjiangzhi Formulation (SEJZ)	Decoction	Hyperlipidemia rats induced by HFD (4 weeks)	SEJZ (30 g/kg/day) by gavage (*n* = 8)	The same volume of saline by gavage (*n* = 8)	-	4 weeks	Prepared according to China Pharmacopoeia	HPLC	Zhang et al. (2022)
Shenling baizhu Powder (SLBZ)	Powder	NAFLD rats induced by HFD (16 weeks)	SLBZ (30 g/kg/day) by gavage (*n* = 11)	The same volume of distilled water by gavage (*n* = 12)	Probiotics (0.6 g/kg/day) by gavage (*n* = 12)	16 weeks	Prepared according to China Pharmacopoeia	HPLC-MS	Zhang et al. (2018)
Shenlingbaizhu Powder (SLBZ)	Powder	NAFLD rats induced by HFD (8 weeks)	SLBZ (2.34/4.68/9.36 g/kg/day) by gavage (*n* = 8/8/8)	The same volume of saline by gavage (*n* = 8)	Probiotics (8.4 g/kg/day) by gavage (*n* = 8)	4 weeks	Prepared according to China Pharmacopoeia	HPLC-MS	Hong (2021)
Si Miao Formula (SMF)	Decoction	NAFLD rats induced by high fat/high sucrose diet (16 weeks)	SMF (10/20 g/kg/day) by gavage (*n* = 8/8)	The same volume of purified water by gavage (*n* = 8)	-	16 weeks	Prepared according to China Pharmacopoeia	LC-MS	Han et al. (2021)
Si Ni San (SNS)	Decoction	NAFLD rats induced by HFD (12 weeks)	SNS (5 g/kg/day) by gavage (*n* = 8)	The same volume of distilled water by gavage (*n* = 8)	-	12weeks	Prepared according to China Pharmacopoeia	HPLC	Zhu et al. (2019)
Tanyutongzhi Formula (TYTZ)	Powder	AS mice induced by HFD (8 weeks)	TYTZ (637 mg/kg/day) by gavage (*n* = 6)	The same volume of distilled water by gavage (*n* = 6)	Atorvastatin (1 mg/kg/day) by gavage (*n* = 6)	8 weeks	Prepared according to China Pharmacopoeia	-	Wang et al. (2018)
Tian Huang Formula (THF)	Decoction	Hyperlipidemia rats induced by HFD (14 weeks)	THF (100 mg/kg/day) by gavage (*n* = 10)	The same volume of distilled water by gavage (*n* = 10)	-	10 weeks	Prepared according to China Pharmacopoeia	UPLC-TOF/MS	Yang et al. (2022)
Tian Huang Formula (THF)	Decoction	NAFLD rats induced by HFD (12 weeks)	THF (60/120 mg/kg/day) by gavage (*n* = 10/10)	The same volume of ultrapure water by gavage (*n* = 10)	Atorvastatin (1.5 mg/kg/day) by gavage (*n* = 10)	10 weeks	Prepared according to China Pharmacopoeia	-	Pang (2021)
Tongmai Zhuyu Decoction (TMZY)	Decoction	AS rats induced by HFD (8 weeks)	TMZY (1.224 g/kg/day) by gavage (*n* = 8)	The same volume of saline by gavage (*n* = 8)	Atorvastatin (2 mg/kg/day) by gavage (*n* = 8)	8 weeks	Prepared according to China Pharmacopoeia	HPLC	Ji et al. (2020)
Xiangsha Liujunzi Decoction (XSLJZ)	Decoction	Hyperlipidemia rats induced by HFD (14 weeks)	XSLJZ (11.34 g/kg/d) by gavage (*n* = 8)	The same volume of saline by gavage (n = 8)	-	4 weeks	Prepared according to China Pharmacopoeia	-	Wang et al. (2022)
Xiaoyao San (XYS)	Granules	NAFLD rats induced by HFD (8 weeks)	XYS (0.6 g/kg/d) by gavage (*n* = 6)	The same volume of saline by gavage (*n* = 9)	Atorvastatin (0.9 mg/kg/day) by gavage (*n* = 6)	2 weeks	Prepared according to China Pharmacopoeia	-	Zhou et al. (2021)
Xiexin Decoction (XXD)	Decoction	AS mice induced by HFD (12 weeks)	XXD (1.3 g/kg/d) by gavage (*n* = 8)	The same volume of deionized water by gavage (*n* = 8)	-	12 weeks	Prepared according to China Pharmacopoeia	-	Chen et al. (2020)
Xiexin Decoction (XXD)	Decoction	AS mice induced by HFD (3 months)	XXD (1.3 g/kg/d) by gavage (*n* = 5)	The same volume of ultrapure water by gavage (*n* = 5)	-	3 months	Prepared according to China Pharmacopoeia	-	Lei (2020)
Yinchenhao Decoction	Decoction	NAFLD rats induced by HFD (14 weeks)	Yinchenhao Decoction (3.6 g/kg/d) by gavage (*n* = 10)	The same volume of saline by gavage (*n* = 10)	-	2 weeks	Prepared according to China Pharmacopoeia	LC-MS	Li et al. (2019)
Zexie Tang (ZXT)	Decoction	Hyperlipidemic rats induced by HFD (4 weeks)	ZXT (2.2 g/kg/day) by gavage (*n* = 10)	The same volume of saline by gavage (*n* = 10)	Simvastatin (2.1 mg/kg/day) by gavage (*n* = 10)	4 weeks	Prepared according to China Pharmacopoeia	-	Xu et al. (2017)
Compound Danshen Dripping Pills	Pills	NAFLD mice induced by HFD (12 weeks)	Compound Danshen Dripping Pills (13.5/27/40.5 mg/kg/d) by gavage (*n* = 8/8/8)	The same volume of distilled water by gavage (*n* = 8)	-	12 weeks	Prepared according to China Pharmacopoeia	-	Zhang et al. (2020)
Danlou Tablet (DLT)	Pills	AS mice induced by HFD (24 weeks)	DLT (0.55 g/kg/d) by gavage (*n* = 15)	The same volume of saline by gavage (*n* = 15)	-	8 weeks	Purchased from Jilin Conair Pharmaceutical Co., Ltd.	-	Sun et al. (2020)
Dengzhan Shengmai Capsules (DZSM)	Capsules	NAFLD rats induced by HFD (6 weeks)	DZSM (720 mg/kg/d) by gavage (*n* = 10)	The same volume of 0.25% CMC-Na by gavage (*n* = 10)	-	6 weeks	Purchased from Yunnan Biogu Pharmaceutical Co., Ltd.	-	Guo et al. (2022)
Guanxinning Tablet (GXNT)	Pills	AS minipigs induced by HFD (28 weeks)	GXNT (162 mg/kg/d) by gavage (*n* = 12)	The same volume of saline by gavage (*n* = 12)	-	12 weeks	Purchased from Chiatai Qinchunbao Pharmaceutical co., Ltd.	-	Yang et al. (2022)
Hugan Qingzhi Tablet (HGQZ)	Pills	NAFLD rats induced by HFD (12 weeks)	HGQZ (1.08 g/kg/d) by gavage (*n* = 8)	The same volume of purified water by gavage (*n* = 8)	-	12 weeks	Prepared according to China Pharmacopoeia	UHPLC-QqQ-MS	Tang et al. (2018)
Hugan Qingzhi Tablet (HGQZ)	Pills	NAFLD rats induced by HFD (12 weeks)	HGQZ (1.08 g/kg/d) by gavage (*n* = 8)	The same volume of purified water by gavage (*n* = 8)	-	12 weeks	Prepared according to China Pharmacopoeia	UHPLC-QqQ-MS	Tang et al. (2018)
Naoxintong Capsule (NXT)	Capsules	Hyperlipidemia rats induced by HFD (6 weeks)	NXT (400/800 mg/kg/d) by gavage (*n* = 6/6)	The same volume of saline by gavage (*n* = 6)	Simvastatin (10 mg/kg/day) by gavage (*n* = 6)	6 weeks	Purchased from Shaanxi Buchang Pharmaceutical Co., Ltd.	HPLC-MS	Lu et al. (2022)
Shanmei Capsule (SM)	Capsules	Hyperlipidemia mice induced by HFD (11 weeks)	SM (0.0375 g/kg/day) by gavage (*n* = 15)	The same volume of distilled water by gavage (*n* = 15)	Atorvastatin (10 mg/kg/day) by gavage (*n* = 15)	11 weeks	Purchased from Shanxi Taihang Pharmaceutical Co., Ltd.	UPLC-Q-TOF/MS	Du et al. (2022)
Tongxinluo Capsules (TXL)	Capsules	AS rabbits induced by 1% choline (8 weeks)	TXL (0.6 g/kg/day) by gavage (*n* = 10)	The same volume of saline by gavage (*n* = 10)	Atorvastatin (3 mg/kg/day) by gavage (*n* = 10)	4/8/12 weeks	Purchased from Shijiazhuang Yiling Pharmaceutical Co., Ltd.	-	Qi et al. (2022)

**TABLE 4:** Summary of natural medicine extracts used in animal experiments.

Intervention	Model	Treatment method and sample size			Treatment	Chemical analysis	References
		Treatment group	Negative control group	Positive control group	Duration		
Baicalein	NAFLD mice induced by HFD (5 weeks)	Baicalein (100/200 mg/kg/day) by gavage (*n* = 10/10)	The same volume of saline by gavage (*n* = 10)	Silymarin (200 mg/kg/day) by gavage (*n* = 10)	5 weeks	-	Li et al. (2022)
Berberine (BBR)	Hyperlipidemia rats induced by HFD (8 weeks)	BBR (260 mg/kg/day) by gavage (*n* = 8)	The same volume of purified water by gavage (*n* = 8)	-	8 weeks	-	Xu et al. (2017)
	NAFLD rats induced by HFD (8 weeks)	BBR (150 mg/kg/day) by gavage (*n* = 8)	The same volume of saline by gavage (*n* = 8)	-	4 weeks	-	Li et al. (2019)
	AS mice induced by HFD (14 weeks)	BBR (0.5 g/kg/day) by gavage (*n* = 6)	The same volume of purified water by gavage (*n* = 6)	-	14 weeks	-	Zhu et al. (2018)
	AS mice induced by HFD (13 weeks)	BBR (50/100 mg/kg/day) by gavage (*n* = 12/12)	The same volume of saline by gavage (*n* = 12)	-	13 weeks	-	Wu et al. (2020)
	AS hamsters induced by HFD (10 months)	BBR (100/200 mg/kg/day) by gavage (*n* = 7/7)	The same volume of saline by gavage (*n* = 7)	-	3 months	-	Ma et al. (2022)
	AS mice induced by choline diet (16 weeks)	BBR (100/200 mg/kg/day) by gavage (*n* = 10/10)	The same volume of purified water by gavage (*n* = 10)	-	16 weeks	-	Li et al. (2020)
Berberrubine (BRB)	NAFLD mice induced by HFD (10 weeks)	BRB (10/20/40 mg/kg/day) by gavage (*n* = 10/10/10)	The same volume of 0.5% CMC-Na by gavage (*n* = 10)	Metformin (40 mg/kg/day) by gavage (*n* = 10)	4 weeks	-	Yang et al. (2022)
*Rhizoma Coptidis* (RC) alkaloids	Hyperlipidemia mice induced by HFD (10 weeks)	RC alkaloids (140 mg/kg/day) by gavage (*n* = 8)	The same volume of saline by gavage (*n* = 8)	Palmatine (140 mg/kg/day) by gavage (*n* = 8)	5 weeks	HPLC	He et al. (2016)
*Alisma orientale* extract	Hyperlipidemia rats induced by high fat/high sucrose diet (4 weeks)	*Alisma orientale* extract (1.05 g/kg/day) by gavage (*n* = 8)	The same volume of saline by gavage (*n* = 8)	Metformin (250 mg/kg/day) by gavage (*n* = 8)	4 weeks	-	Li et al. (2019)
Blackberry leaf and fruit extracts (BLF)	NAFLD rats induced by HFD (12 weeks)	BLF (150 mg/kg/day) by gavage (*n* = 10)	The same volume of saline by gavage (*n* = 10)	Milk thistle extracts (150 mg/kg/day) by gavage (*n* = 10)	12 weeks	MS	Park et al. (2019)
*Eucommia ulmoides* extract	AS mice induced by HFD (12 weeks)	*Eucommia ulmoides* extract (0.046 g/kg/day) by gavage (*n* = 10)	The same volume of saline by gavage (*n* = 10)	Rosuvastatin (0.45 mg/kg/day, once/day) by gavage (*n* = 10)	8 weeks	LC-MS	Sun et al. (2020)
Ginkgolide B (GB)	AS mice induced by HFD (6 weeks)	GB (20/30 mg/kg/day) by gavage (*n* = 10/10)	The same volume of saline by gavage (*n* = 10)	Atorvastatin (1.3 mg/kg/day) by gavage (*n* = 10)	6 weeks		Lv et al. (2021)
Green Brick Tea	NAFLD mice induced by HFD (14 weeks)	Green Brick Tea (75/300 mg/kg/day) by gavage (*n* = 12/12)	The same volume of purified water by gavage (*n* = 12)	Xuezhikang Capsule (90 mg/kg/day) by gavage (*n* = 12)	14 weeks	-	Zhou et al. (2021)
*Gynostemma pentaphyllum (Thunb.) Makino* [Cucurbitaceae; *Gynostemmatis herba*] (GP)	Hyperlipidemia rats induced by HFD (8 weeks)	GP (6/9 g/kg/day) by gavage (*n* = 10/10)	The same volume of distilled water by gavage (*n* = 10)	-	8 weeks	HPLC	Huang et al. (2018)
	NAFLD rats induced by HFD (4 weeks)	GP (1.5/3/6 g/kg/day) by gavage (*n* = 10/10/10)	The same volume of distilled water by gavage (*n* = 10)	dilinoleoyl phosphatidylcholine (DLPC) (22.8 mg/kg/day) by gavage (*n* = 10)	4 weeks	HPLC-MS	Shen et al. (2020)
*Luffa cylindrica* (*L.*) *Roem* [Cucurbitaceae; *Luffa aegyptiaca Miller*] (LC)	Obesity mice induced by HFD (14 weeks)	LC (2 g/kg/day) by gavage (*n* = 12–15)	The same volume of saline by gavage (*n* = 12–15)	-	14 weeks	-	Lu et al. (2016)
Macroalgae *Laminaria japonica* (MLJ)	Hyperlipidemia rats induced by HFD (8 weeks)	MLJ (2.5 g/kg/day) by gavage (*n* = 8)	The same volume of saline by gavage (*n* = 8)	-	8 weeks	-	Zhang et al. (2020)
*Momordica charantia* L. [Cucurbitaceae; *Fructus momordicae*] (MC)	Obesity rats induced by HFD (8 weeks)	MC (400 mg/kg/day) by gavage (*n* = 10)	The same volume of purified water by gavage (*n* = 10)	-	8 weeks	GC	Bai (2019)
Paeonol (Pae)	NAFLD rats induced by HFD (9 weeks)	Pae (100/200/300 mg/kg/day) by gavage (*n* = 7/7/7)	The same volume of 0.5% CMC-Na by gavage (*n* = 7)	Silibinin (25 mg/kg/day) by gavage (*n* = 7)	4 weeks	-	Jiang et al. (2021)
	AS mice induced by HFD (14 weeks)	Pae (10/20 mg/kg/day) by gavage (*n* = 10/10)	The same volume of 0.5% CMC-Na by gavage (*n* = 10)	-	14 weeks	-	Liu et al. (2021)
	AS mice induced by HFD (20 weeks)	Pae (20 mg/kg/day) by gavage (*n* = 6)	The same volume of 0.5% CMC-Na by gavage (*n* = 6)	-	20 weeks	-	He (2021)
*Radix scutellariae* water extract	Hyperlipidemia rats induced by HFD (4 weeks)	*Radix scutellariae* water extract (2.5 g/kg/d) by gavage (*n* = 9)	The same volume of distilled water by gavage (*n* = 9)	Metformin (200 mg/kg/day) by gavage (*n* = 9)	4 weeks	UPLC/Q-TOF-MS	Zhao et al. (2021)
*Senna tora* (*L.*) *Roxb.* [Fabaceae; *Cassiae semen*] (ST)	NAFLD mice induced by HFD (17 weeks)	ST (10 g/kg/d) by gavage (*n* = 10)	The same volume of saline by gavage (*n* = 10)	-	3 weeks	HPLC	Luo et al. (2021)
*Usnea diffracta Vain.* [Usneaceae; *Usnea diffracta*] (UD)	AS rats induced by HFD + intraperitoneal VD3 (4 weeks)	UD (0.7/1.4/2.8 g/kg/d) by gavage (*n* = 8/8/8)	The same volume of distilled water by gavage (*n* = 8)	Simvastatin (4 mg/kg/d) by gavage (*n* = 8)	4 weeks	-	Zhang et al. (2021)
*Zanthoxylum bungeanum Maxim.* [Rutaceae; *Zanthoxyli pericarpium*] (ZB)	Hyperlipidemia rats induced by HFD (6 weeks)	ZB (3/6/9 g/kg/d) by gavage (*n* = 8/8/8)	The same volume of soybean oil by gavage (*n* = 8)	-	6 weeks	-	You (2016)
Kaempferol	Obesity mice induced by HFD (8 weeks)	Kaempferol (200 mg/kg/d) by gavage (*n* = 10)	The same volume of purified water by gavage (*n* = 10)	-	8 weeks	-	Wang et al. (2020)
Baicalin	Hyperlipidemia mice induced by HFD (8 weeks)	Baicalin (25/50 mg/kg/day) by gavage (*n* = 8/8)	The same volume of saline by gavage (*n* = 8)	-	4 weeks	-	Liu et al. (2021)
Bilberry anthocyanins	NAFLD mice induced by HFD (13 weeks)	2% Bilberry anthocyanins by gavage (*n* = 5)	The same volume of distilled water by gavage (*n* = 5)	-	13 weeks	-	Nakano et al. (2020)
Ethyl Acetate Extract of *Eleutherococcus senticosus (Rupr. & Maxim.) Maxim.* [Araliaceae, *Acanthopanacis senticosi radix et rhizoma seu caulis*]	AS mice induced by HFD (8 weeks)	Ethyl Acetate Extract of *Eleutherococcus senticosus (Rupr. & Maxim.) Maxim.* [Araliaceae, *Acanthopanacis senticosi radix et rhizoma seu caulis*] (25/75 mg/kg/day, once/day) by gavage (*n* = 8/8)	The same volume of 0.5% CMC-Na by gavage (*n* = 8)	Rosuvastatin (10 mg/kg/day, once/day) by gavage (*n* = 8)	8 weeks	-	Jia et al. (2022)
Luteolin	NAFLD rats by HFD (8 weeks)	Luteolin (50/100 mg/kg/day) by gavage (*n* = 8)	The same volume of saline by gavage (*n* = 8)	Polyene Phosphatidylcholine Capsules (200 mg/kg/day) by gavage (*n* = 8)	4 weeks	-	Liu et al. (2021)
Myricetin	NAFLD rats induced by HFD (12 weeks)	HFD supplemented with 0.5% (w/w) Myricetin (*n* = 8)	A normal chow diet (*n* = 8)	-	12 weeks	-	Sun et al. (2021)
Naringin	AS mice induced by HFD (16 weeks)	Naringin (100 mg/kg/day) by gavage (*n* = 8)	The same volume of 0.5% CMC-Na by gavage (*n* = 8)	Atorvastatin (10 mg/kg/day) by gavage (*n* = 8)	16 weeks	-	Wang et al. (2020)
Quercetin	NAFLD mice by HFD (16 weeks)	HFD supplemented with 0.05% (w/w) Quercetin (*n* = 10)	A normal chow diet (*n* = 10)	-	16 weeks	-	Porras et al. (2017)
	AS mice induced by high cholesterol diet (12 weeks)	Quercetin (100 mg/kg/day) by gavage (*n* = 6)	The same volume of saline by gavage (*n* = 6)	-	12 weeks	-	Wu et al. (2019)
	AS mice induced by HFD (12 weeks)	Quercetin (100 ug/kg/day) by gavage (*n* = 12)	The same volume of purified water by gavage (*n* = 12)	-	12 weeks	-	Nie et al. (2019)
Gastrodin	AS mice induced by HFD (12 weeks)	Gastrodin (50/100/200 mg/kg/day) by gavage (*n* = 8/8/8)	The same volume of saline by gavage (*n* = 8)	-	8 weeks	-	Liu et al. (2021)
Chitooligosaccharide (COSM)	NAFLD mice induced by high fat/high sucrose diet (8 months)	COSM (425/850/1700 mg/kg/day) by gavage (*n* = 6/6/6)	The same volume of ultrapure water by gavage (*n* = 6)	Metformin (50 mg/kg/day) by gavage (*n* = 6)	12 weeks	-	Feng et al. (2022)
Porphyran-derived oligosaccharides (PYOs)	NAFLD mice induced by HFD (6 months)	PYOs (100/300 mg/kg/day) by gavage	The same volume of saline by gavage	-	6 weeks	UPLC-MS/MS	Wang et al. (2021)
Caffeic acid	NAFLD rats induced by HFD (8 weeks)	0.08%/0.16% (w/w) Caffeic acid (*n* = 10/10)	A normal chow diet (*n* = 10)	-	8 weeks	-	Mu et al. (2021)
Black tea polyphenols (BTP)	Obesity mice induced by high fat/high sucrose diet (4 weeks)	High fat/high sucrose diet supplemented with BTP (320 mg/kg/day) (*n* = 12)	A low-fat diet (*n* = 12)	-	4 weeks	HPLC and LC-MS/MS	Henning et al. (2018)
Burdock inulin	NAFLD mice induced by HFD (16 weeks)	Naringin (100 mg/kg/day) by gavage (*n* = 8)	The same volume of saline by gavage (*n* = 10)	Simvastatin (20 mg/kg/d) by gavage (*n* = 10)	4 weeks	Phenol-sulfuric acid	Wang et al. (2020)
Citrus Peel Powder extract (CPP)	NAFLD rats induced by HFD (12 weeks)	CPP (1 g/kg/day) by gavage (*n* = 8)	The same volume of phosphate-buffered saline (PBS) by gavage (*n* = 8)	-	12 weeks	UPLC-MS/MS	Hu et al. (2021)
Epigallocatechin gallate (EGCG)	NAFLD mice induced by HFD (14 weeks)	HFD supplemented with 0.4% (w/w) EGCG (*n* = 12)	A low-fat diet (*n* = 12)	-	14 weeks	-	Li et al. (2020)
	NAFLD rats induced by HFD (8 weeks)	EGCG (100/200 mg/kg/day) by gavage (*n* = 8/8)	The same volume of distilled water by gavage (*n* = 8)	-	8 weeks	-	Zuo (2020)
Fucoidan	Hyperlipidemia mice induced by HFD (5 weeks)	Fucoidan (50/250 mg/kg/day) by gavage (*n* = 10/10)	The same volume of saline by gavage (*n* = 10)	-	5 weeks	-	Huang et al. (2019)
Honokiol	Obesity mice induced by HFD (8 weeks)	Honokiol (200/400/800 mg/kg/day) by gavage (*n* = 24)	The same volume of purified water by gavage (*n* = 24)	-	8 weeks	-	Ding et al. (2019)
Inulin	NAFLD mice induced by HFD (14 weeks)	Inulin (5 g/kg/day) by gavage (*n* = 15)	The same volume of purified water by gavage (*n* = 15)	-	14 weeks	-	Bao (2021)
	NAFLD mice induced by HFD (8 weeks)	HFD supplemented with 10% (w/w) Inulin (*n* = 10)	A normal chow diet (*n* = 10)	-	8 weeks	-	Perez-Monter et al. (2022)
	AS mice induced by HFD (12 weeks)	Inulin (10 g/kg/day) by gavage (*n* = 15)	The same volume of purified water by gavage (*n* = 15)	-	12 weeks	-	Wang et al. (2021)
L. caerulea L. berry polyphenols (LCBP)	NAFLD mice by HFD (45 days)	0.5%/1% (w/w) LCBP by gavage (*n* = 4/4)	The same volume of purified water by gavage (*n* = 4)	-	45 days	HPLC	Wu et al. (2018)
Noni fruit polysaccharide (NFP)	NAFLD mice by HFD (4 weeks)	NFP (100 mg/kg/d) by gavage (*n* = 9)	The same volume of saline by gavage (*n* = 9)	-	5 weeks	-	Yang et al. (2020)
polyphenol-rich loquat fruit extract (LFP)	NAFLD mice induced by 30% high-fructose water (8 weeks)	LFP (25/50 mg/kg/d) by gavage (*n* = 10/10)	The same volume of distilled water by gavage (*n* = 10)	-	8 weeks	UHPLC-QqQ-MS/MS	Li et al. (2019)
Proanthocyanidin	Hyperlipidemia rats induced by HFD (8 weeks)	Proanthocyanidin (25/100/150 mg/kg/d) by gavage (*n* = 10/10/10)	The same volume of distilled water by gavage (*n* = 10)	Fenofibrate (80 mg/kg/d) by gavage (*n* = 10)	8 weeks	-	Fu et al. (2013)
Resveratrol (RSV)	NAFLD mice induced by HFD (6 weeks)	RSV (50/100 mg/kg/d) by gavage (*n* = 10/10)	The same volume of 0.5% CMC-Na by gavage (*n* = 10)	Simvastatin (10 mg/kg/d) by gavage (*n* = 10)	4 weeks	-	Chen et al. (2019)
	NAFLD mice induced by HFD (24 weeks)	RSV (45 mg/kg/d) by gavage (*n* = 12)	The same volume of 0.5% CMC-Na by gavage (*n* = 12)	-	6 weeks	-	Yao (2017)
	NAFLD mice induced by HFD (6weeks)	RSV (100 mg/kg/d) by gavage (*n* = 10)	The same volume of saline by gavage (*n* = 10)	-	6 weeks	-	Chen et al. (2020)
	AS mice induced by 1% choline	HFD supplemented with 0.4% (w/w) RSV (*n* = 10)	A normal chow diet (*n* = 10)	-	4 months	-	Chen et al. (2016)
Theabrownins	Hyperlipidemia mice induced by HFD (26 weeks)	Theabrownins (450 mg/kg/d) by gavage (*n* = 8)	The same volume of distilled water by gavage (*n* = 8)	-	26 weeks	-	Huang et al. (2019)
*Ganoderma lucidum* polysaccharide (GLP)	Obesity mice induced by HFD (12 weeks)	GLP (100/300 mg/kg/d) by gavage (*n* = 6/6)	The same volume of distilled water by gavage (*n* = 6)	-	12 weeks	GC	Sang et al. (2021)
*Ganoderma lucidum* polysaccharide and chitosan (PC)	Hyperlipidemia hamsters induced by HFD (8 weeks)	PC (150 mg/kg/d) by gavage (*n* = 6)	The same volume of saline by gavage (*n* = 6)	Silymarin (150 mg/kg/d) by gavage (*n* = 6)	8 weeks	-	Tong et al. (2020)
*Laminaria japonica* polysaccharide (LJP)	NAFLD mice induced by HFD (8 weeks)	HFD supplemented with 2.5%/5% (w/w) LJP (*n* = 10/10)	A normal chow diet (*n* = 10)	-	8 weeks	HPLC	Zhang et al. (2021)
*Lycium barbarum* polysaccharide (LBPs)	NAFLD rats induced by HFD (8 weeks)	LBPs (50 mg/kg/d) by gavage (*n* = 10)	The same volume of saline by gavage (*n* = 10)	-	8 weeks	HPLC	Gao et al. (2021)
Procyanidin B2 (PB2)	NAFLD rabbits induced by HFD (12 weeks)	PB2 (150 mg/kg/d) by gavage (*n* = 8)	The same volume of saline by gavage (*n* = 8)	-	12 weeks	-	Xing et al. (2019)
Rosa Laevigata Michx. Fruits Polysaccharides (RLPs)	NAFLD mice induced by HFD (12 weeks)	RLPs (200/800 mg/kg/d) by gavage (*n* = 10/10)	The same volume of 5% gum Arabic solution by gavage (*n* = 10)	-	12 weeks	HPLC	Zhang et al. (2020)
Rosa roxburghii Tratt polysaccharide (RTFP)	NAFLD mice induced by HFD (9 weeks)	RTFP (200/400 mg/kg/d) by gavage (*n* = 10/10)	The same volume of saline by gavage (*n* = 10)	-	7 weeks	HPLC	Zhang et al. (2022)
water insoluble polysaccharide (WIP) from the sclerotium of *Poria cocos (Schw.) Wolf* [Polyporaceae; *Poria*]	Hyperlipidemia mice induced by HFD (4 weeks)	WIP (0.5/1 g/kg/d) by gavage (*n* = 10/10)	The same volume of purified water by gavage (*n* = 10)	Inulin (5 g/kg/day) by gavage (*n* = 15)	4 weeks	HPLC	Sun et al. (2019)
Gynostemma pentaphyllum saponins (GPS)	NAFLD rats induced by HFD (8 weeks)	GPS (50/100/150 mg/kg/d) by gavage (*n* = 8/8/8)	The same volume of purified water by gavage (*n* = 8)	-	8 weeks	HPLC	Zhong et al. (2022)
*Ilex pubescens* triterpenoid saponins (IPTS)	AS rats induced by HFD (8 weeks) + intraperitoneal VD3 (3rd day)	IPTS (60 mg/kg/d) by gavage (*n* = 10)	The same volume of 0.5% CMC-Na by gavage (*n* = 10)	-	8 weeks	Colorimetric method	Bai (2019)
	AS rats induced by HFD (8 weeks) + intraperitoneal VD3 (3rd day)	IPTS (30/60/120 mg/kg/d) by gavage (*n* = 10/10/10)	The same volume of 0.5% CMC-Na by gavage (*n* = 10)	Simvastatin (5 mg/kg/d) by gavage (*n* = 10)	8 weeks	Colorimetric method	Bai (2019)
Tea seed saponins	Hyperlipidemia rats induced by HFD (4 weeks)	Tea seed saponins (50/100/150 mg/kg/d) by gavage (*n* = 10/10/10)	The same volume of 0.5% CMC-Na by gavage (*n* = 10)	Simvastatin (10 mg/kg/d) by gavage (*n* = 10)	4 weeks	-	Lin et al. (2020)

In summary, intestinal microecology plays a significant part in the development of LMD, and the regulation of intestinal flora and its metabolites is a potential new therapeutic target for LMD. TCM has obtained some achievements in improving lipid metabolism disorder diseases, probably by regulating intestinal flora and its metabolites, but in order to clarify the precise mechanism of action, more extensive research will still be required in the future.
