# Supplementary material for: Intestinal flora: A new target for traditional Chinese medicine to improve lipid metabolism disorders
Source: Front Pharmacol. 2023 Mar 1;14:1134430. doi: 10.3389/fphar.2023.1134430 (PMC10014879; doi:10.3389/fphar.2023.1134430)
Supplement: Supplementary file 1 [file Table1.docx]

Supplementary table 1. Therapeutic effects of natural medicine extracts on LMD.

| Type | Intervention | Model | Outcome | Changes in intestinal flora | Potential Mechanism | References |
| --- | --- | --- | --- | --- | --- | --- |
| Alkaloids | Baicalein | NAFLD  C57BL/6N mice | 1. BW, Liver weight, epididymal fat weight↓;  2. Serum TC, TG, LDL-C, ALT, AST↓, HDL-C↑. | *Anaerotruncus, Lachnoclostridium, Atopostipes, Mucispirillum↓.* | 1. Signaling pathway: altered alphaLinolenic acid, 2-Oxocarboxylic acid, Pantothenate and CoA biosynthesis, bile secretion. | Li et al. (2022) |
|  | Berberine (BBR) | Hyperlipidemia  SD rats | 1. BW↓;  2. Serum TC, LDL-C, ALT, FFA↓;  3. Hepatic steatosis↓. | *Coprococcus, Lachnospiraceae, Roseburia, Ruminococcus, Faecalibacterium, Desulfovibrio↓.* | 1. Inflammatory: IL-1β, NADPHox, CD68, TLR4, LBP↓;  2. Intestinal barrier: ZO-1, claudin-1*↑*. | Xu et al. (2013) |
|  |  | NAFLD  SD rats | 1. BW↓;  2. Serum TG, ALT, AST↓;  3. Hepatic steatosis↓. | *Bacteroides↑.* | 1. Inflammatory: LPS, I-FABP↓. | Li et al. (2015) |
|  |  | AS  ApoE^-/-^ mice | 1. Serum TC, TG↓;  2. Atherosclerotic plaque area↓. | *Akkermansia↑.* | 1. Inflammatory: Ileum TNF-α, IL-1β, Colon TNF-α, IL-6, Carotid artery MMP-2, VCAM-1↓;  2. Intestinal barrier: Ileum ZO-1, Colon Occludin*↑*. | Zhu et al. (2018) |
|  |  | AS  ApoE^-/-^ mice | 1. Serum TC, TG, LDL-C, APOB100, VLDL-C, LP↓. | *Turicibacter, Alistipes, Roseburia, Allobaculum, Blautia↑.* | 1. Inflammatory: TNF-α, IL-1β, IL-6↓, IL-10, ADPN*↑*. | Wu et al. (2020) |
|  |  | AS  hamsters | - | *Eubacterium_coprostanoligenes, Treponema_2, Ruminococcaceae_UCG-002, Prevotellaceae_UDG-001, Flavobacterium, Candidatus_Saccharimonas, Empedobacter, Corynebacterium_1, Jeotgalicoccus, Myroides, Kurthia, Acinetobacter and Ruminoccoccus_2↓; Allobaculum, Akkermansia, Lachnospiraceae_NK4A136↑.* | 1. Serum and fecal TMA, TMAO↓. | Ma et al. (2022) |
|  |  | AS  C57BL/6Jand ApoE^-/-^ mice | 1. Urea↓. | *Bacteroides, Prevotella, Parabacteroides, Alloprevotella, Lachnospiraceae NK4A136 group, Bacteroidales S24-7 group (unclassified), Eubacterium↑.* | 1. BAs: BSH*↑*, T-βMCA/β-MCA↓;  2. Serum TMA, TMAO, liver FMO3, cutC, cntA↓. | Li et al. (2021) |
|  | Berberrubine (BRB) | NAFLD  C57BL/6J mice | 1. BW↓;  2. Serum TG↓, liver TC, TG↓, HDL-C↑. | *F/B* ratio*, Lactobacillus, Romboutsia↓; Ileibacterium, Mucispirillum↑.* | 1. Lipid metabolism: ATGL, GK, PPAR-α and CPT-1↑, ACC1, FAS and CD36↓. | Yang et al. (2022) |
|  | *Rhizoma Coptidis* (RC) alkaloids | Hyperlipidemia  B6 mice | 1. BW↓;  2. Serum TC, TG, LDL-C↓. | *Parabacteroides, Desulfovibrio C21_c20, Escherichia coli↓; Akkermansia, Prevotella, Helicobacter, Bacteroides, Alcaligenes faecalis↑.* | 1. Inflammatory: LPS, JNK, TLR4↓, SREBP2↑;  2. BAs: TBA↓;  3. BAs-related receptor expression: ASBT↓, FXR, TGR5, CYP7A1↑. | He et al. (2016) |
| Extracts | *Alisma orientale* extract | Hyperlipidemia  SD rats | 1. Serum TC, LDL-C↓;  2. liver weight↓. | *Firmicutes, Lactobacillus, Roseburia, Blautia↓; Bacteroidetes, Proteobacteria, Tenericutes, Verrucomicrobia, Phascolarctobacterium, Bacteroides, Akkermansia↑.* | - | Li et al. (2019) |
|  | Blackberry leaf and fruit extracts (BLF) | NAFLD  SD rats | 1. BW, liver weight↓;  2. Hepatic TC, TG↓, Serum TC, TG, LDL-C, ALT, AST↓. | *Lactobacillus, Akkermansia↑.* | 1. Oxidative stress: Hepatic SOD, GSH, GSH-Px↑, MDA↓;  2. Inflammatory: Hepatic TNF-α↓, serum TNF-α, IL-β1↓. | Park et al. (2019) |
|  | *Eucommia ulmoides* extract | AS  ApoE^−/−^ mice | - | *Helicobacter, Staphylococcus↓; Lactobacillus, faecalis, Bacteroides, odoribacter↑.* | 1. Inflammatory: CAM-1, IL-6↓, TGF-β1↑;  2. TMAO↓. | Sun et al. (2020) |
|  | Ginkgo biloba extract (GbE) | AS  Ldlr (^−/−^) mice | - | *F/B* ratio*↓; Akkermansia Alloprevotella, Alistipes, Parabacteroides↑.* | 1. SCFAs↑;  2. BAs: secondary BAs↑. | Wang et al. (2022) |
|  | Ginkgolide B (GB) | AS  C57/BL6 ApoE^−/−^ mice | 1. Serum TC, TG, LDL-C, Liver TC, TG↓, HDL-C↑;  2. Atherosclerotic plaque area↓. | *Firmicutes, Deferribacteres, Helicobacter, Roseburia↓; Bacteroidetes, Prevotella, Bacteroides↑.* | 1. Inflammatory: hs-CRP↓;  2. FMO3, Serum TMA, TMAO, kidney TMAO↓. | Lv et al. (2021) |
|  | Green Brick Tea | NAFLD  C57BL/6 mice | 1. BW, liver weight, liver index↓;  2. Serum TC, LDL-C/HDL-C, ALT↓;  3. Hepatocyte fat vacuoles, lipid droplet deposition↓. | *Bacteroides↓; Lactobacillus, Alloprevotella, Saccharibacteria_genera_incertae_sedis↑.* | 1. Inflammatory: IL-18, IL-1β↓. | Zhou et al. (2021) |
|  | *Gynostemma pentaphyllum (Thunb.) Makino* [Cucurbitaceae; *Gynostemmatis herba*] (GP) | Hyperlipidemia  SD rats | 1. Serum TC, LDL-C↓;  2. Liver TC, TG↓;  3. Serum MDA↓, SOD↑. | *Firmicutes, F/B* ratio, *Clostridium_XI↓; Bacteroidetes, Clostridium_XIVa, Prevotella↑.* | - | Huang et al. (2018) |
|  |  | NAFLD  SD rats | 1. Serum TC, TG, LDL-C, AST, ALT↓, HDL-C↑;  2. Hepatic steatosis↓. | *F/B* ratio, *Ruminococcus↓; Elusimicrobia, Cyanobacteria, Lactococcus↑.* | 1. Inflammatory: Serum and hepatic TLR-4, IL-6, LPS, TNF-α↓. | Shen et al. (2020) |
|  | *Luffa cylindrica (L.) Roem* [Cucurbitaceae; *Luffa aegyptiaca Miller*] (LC) | Obesity  C57BL/6J mice | 1. BW↓;  2. serum TC, TG, FFA↓; fecal TG↑. | Norank_*f_Bacteroidates_S24-7 group*↓*;* *Desulfovibrio*, *Ruminiclostridium_9*, *Ruminiclostridium*, *Blautia*, norank*_f_Lachnospiraceae*, *Blautia*↑. | 1. Intestinal Barrier: JAM-A, occluding, mucin2↑;  2. Inflammation: TNF-a, MCP-1 in eWAT and liver↓, PPAR-α， PGC1-α in eWAT↑;  3. Lipid Metabolism: FABP4, CD36, FAS in eWAT↓; FABP4, FATP1, CD36, FAS, SREBP1c, PPAR-γ in liver↓; SCD1 in liver↑. | Lu et al. (2019) |
|  | Macroalgae Laminaria japonica (MLJ) | Hyperlipidemia  SD rats | 1. Serum TC, TG, BA↓, fecal TC, TG, BA↑. | *Enterococcus, Flavifiexus↓;* *Allobaculum, Phascolarctobacterium, Ruminiclostridiu↑.* | 1. SCFAs: Fecal acetate, propionate, butyrate, valerate*↑*. | Zhang et al. (2020) |
|  | *Momordica charantia L.* [Cucurbitaceae; *Fructus momordicae*] (MC) | Obesity  SD rats | 1. BW, Lee index↓;  2. Serum TC, TG↓, HDL-C↑. | *Enterobacteriaceae↓; Verrucomicrobia,* *Blautia, Allobaculum, Butyricicoccus, Akkermansia↑.* | 1. Inflammatory: IL-6, LPS, TNF-α, MCP-1, LBP↓;  2. Intestinal barrier: ZO-1, Occludin↑;  3. SCFAs: Valeric acid↓, Acetic acid, propionic acid, butyric acid↑;  4. Signaling pathway: NF-κB/JNK/MAPKs↓. | Bai et al. (2019) |
|  | Paeonol (Pae) | NAFLD  SD rats | 1. Serum FFA↓;  2. Hepatic steatosis↓. | *Lachnospira↓.* | 1. Inflammatory: TNF-α, IL-1β, IL-2, IL-6, IL-8, NF-κB↓. | Jiang et al. (2019) |
|  |  | AS  ApoE^−/−^ mice | 1. Atherosclerotic plaque area↓. | *F/B* ratio*,* *Acetatifactor, Helicobacter↓;* *Bacteroides, Alistipes↑.* | 1. Inflammatory: ASC, TXNIP, NLRP3, cleaved caspase-1, cleaved IL-1β, TNF-α, IL-1β, IL-18↓;  2. Oxidative stress: MDA, ROS↓;  3. Signaling pathway: TXNIP/NLRP3↓. | Liu et al. (2021) |
|  |  | AS  ApoE^−/−^ mice | 1. Atherosclerotic plaque area↓. | *Proteobacteria, Helicobacter↓; F/B* ratio*, Bacteroides, Lactobacillus↑.* | 1. BAs: Secondary BAs, Fecal BAs, TαMCA, T-βMCA↑, CDCA, LCA↓;  2. Signaling pathway: FXR-FGF15 axis↓. | He et al. (2021) |
|  | *Penthorum chinense Pursh.* (PCP) extract | NAFLD  C57BL/6J mice | 1. BW↓. | *BSH-producing bacteria↓.* | 1. BAs: Fecal T-βMCA, TUDCA, TCDCA, CDCA↑;  2. Signaling pathway: Intestine FXR/FGF15↓, CYP27A1, CYP7B1↑, Hepatic FXR↑. | Li et al. (2022) |
|  | *Radix scutellariae* water extract | Hyperlipidemia  SD rats | 1. BW↓;  2. Serum TG↓. | *Lactobacillus, feacalibaculum↓; Tenericutes, Patescibacteria,* *Blautia, Lachnoclostridium, Ruminiclostridium_5, Turicibacter↑.* | 1. BAs: Serum DCA, LCA, GDCA, GLCA, GUDCA, TLCA↓, fecal DCA, LCA, GDCA, GLCA, GUDCA, TLCA↑;  2. Signaling pathway: FXR axis↓, CYP7A1↑. | Zhao et al. (2021) |
|  | *Senna tora (L.) Roxb.* [Fabaceae; *Cassiae semen*] (ST) | NAFLD  C57BL/6 mice | 1. Liver index, liver weight↓;  2. Serum TC, TG, LDL-C, FFA, ALT, AST↓. | *Erwinia, Klebsiella, Morganella, Trabulsiella↓; Coprococcus, Lachnospiraceae, Blautia, Roseburia, Dehalobacterium, Oscillospira, Ruminococcus↑.* | 1. Inflammatory: LPS, TNF-α, IL-6↓, IL-10↑;  2. Intestinal barrier: Occludin, ZO-1 mRNA↑. | Luo et al. (2021) |
|  | *Usnea diffracta Vain.* [Usneaceae; *Usnea diffracta*] (UD) | AS  SD rats | 1. Atherosclerotic plaque area↓. | *Proteobacteria, F/B* ratio*, Pseudomonas, Ralstonia, Staphylococcus↓; Bacteroidetes, Parabacteroides, Escherichia-Shigella, Enterobacter, Akkermansia, Enterococcus↑.* | 1. Inflammatory: IL-6, LPS, TNF-α↓;  2. Intestinal barrier: ZO-1, Occludin*↑*. | Zhang et al. (2021) |
|  | *Zanthoxylum bungeanum Maxim.* [Rutaceae; *Zanthoxyli pericarpium*] (ZB) | Hyperlipidemia  SD rats | 1. Serum and liver TC, TG↓. | *Escherichia coli, Enterococcus↓;* *Bifidobacteria↑.* | 1. SCFAs↑;  2. BAs: Fecal BAs↑. | You et al. (2016) |
| Flavanol | Kaempferol | Obesity  C57BL/6J mice | 1. BW, liver weight, iWAT weight, eWAT weight, pWAT weight↓;  2. Serum TC, TG, LDL-C↓; | *Firmicutes↓; Bacteroidetes, Proteobacteria, Akkermansia, Bacteroides, Lactobacillus↑.* | - | Wang et al. (2020) |
| Flavonoids | Baicalin | Hyperlipidemia  C57BL/6J mice | - | *Desulfovibrio↓.* | 1. Inflammatory: LPS, IL-6, TNF-α↓. | Liu et al. (2016) |
|  | Bilberry anthocyanins | NAFLD  C57BL/6J mice | 1. Serum TC, LDL-C, AST, ALT↓, HDL-C↑. | *F/B ratio, Deferribactere, S24-7, Prevotella, Lactobacillales, Clostridiales↓; Verrucomicrobia, Bacteroides, Akkermansia, Parabacteroides↑.* | 1. Inflammatory: MCP-1↓;  2. SCFAs: Caecum Lactic acid↑, Butyric acid↓;  3. Signaling pathway: Nrf2↑. | Nakano et al. (2020) |
|  | Ethyl Acetate Extract of *Eleutherococcus senticosus (Rupr. & Maxim.) Maxim.* [Araliaceae, *Acanthopanacis senticosi radix et rhizoma seu caulis*] | AS  ApoE^-/-^ mice | 1. Serum TC, TG, LDL-C↓, HDL-C↑;  2. Atherosclerotic plaque area↓. | *Firmicutes, F/B* ratio*↓; Bacteroidetes, Lactobacillus, Alistipes, Odoribacter, Desulfovibrio↑.* | 1. Inflammatory: IL-6, IL-7, TNF-α↓;  2. Oxidative stress: iNOS↓, SOD↑. | Jia et al. (2022) |
|  | Luteolin | NAFLD  Wistar rats | 1. BW, liver index↓;  2. Serum TC, TG, LDL-C, AST, ALT↓, HDL-C↑, Liver TC, TG↓;  3. Liver lipid deposition↓. | *F/B* ratio, *Actinobacteria↓; Proteobacteria, Bifidobacterium, Lactobacillus↑.* | 1. Inflammatory: LPS, IL-6, TNF-α, TLR-4, NF-κB, IL-1β↓;  2. Intestinal barrier: ZO-1, claudin-1, occludin*↑*;  3. Signaling pathway: TLR4/ NF-κB↓. | Liu et al. (2022) |
|  | Myricetin | NAFLD  Wistar rats | 1. Serum TC, TG, LDL-C, ALT, AST↓, HDL-C↑. | *F/B* ratio*↓; Actinobacteria, Dermabacteriaceae, Coriobacteriaceae, Allobaculum, Brachybacterium↑.* | 1. Inflammatory: LPS, IL-6, TNF-α↓;  2. SCFAs: Butyric acid↑;  3. Intestinal barrier: ZO-1*↑*;  4. Signaling pathway: TLR4/NF-κB↓. | Sun et al. (2021) |
|  | Naringin | AS  ApoE^-/-^ mice | 1. Serum TC, LDL-C, ox-LDL↓；  2. Fecal total lipid, neutral sterol↓. | *Bacteroidetes, Verrucomicrobia, Bacteroides, Bifidobacterium, Lactococcus, Clostridium sensu_stricto_1↓; Firmicutes, Eubacterium_fissicatena, Eubacterium_coprostanoligenes, Eubacterium_brachyw↑.* | 1. BAs: Fecal TBA, CA, TUDCA, UDCA, CG, Tα/βMCA↓;  2. Amino Acid Metabolism: glycine, taurine↓;  3. Signaling pathway: FXR-FGF15 axis↓, CYP7A1↑. | Wang et al. (2020) |
|  | Quercetin | NAFLD  C57BL/6J mice | 1. BW, Liver weight↓;  2. Serum TG, ALT, FFA↓. | *Desulfovibrio↓; Lactobacillus, Flavobacterium, Allobaculum, Sutterella↑.* | 1. Inflammatory: LPS, IL-6↓;  2. SCFAs: Acetate, propionate, butyrate↑;  3. Intestinal barrier: Occludin, Claudin 1↑;  4. Signaling pathway: TLR4↓, NLRP3, caspase-1↓. | Porras et al. (2017) |
|  |  | AS  ApoE^-/-^ mice | 1. Serum TC, TG, LDL-C↓, HDL-C↑;  2. Vascular fibrosis level↓. | *Bamesiella, Lactobacillus, Phascolarctobacterium↓.* | 1. Inflammatory: TNF-α, IL-6↓;  2. Signaling pathway: primary bile acid biosynthesis. | Wu et al. (2019) |
|  |  | AS  Ldlr mice | 1. BW↓;  2. Cecal Cholesterol↓, Coprostanol↑;  3. Atherosclerotic lesions areas, plaques sizes, Lipid deposition↓. | *Firmicutes, Lactobacillus, Verrocomicrobia↓; Bacterioidetes, Actinobacteria, Akkermansia, Bacteroides, Cyanobacteria, Parabacteroides, Ruminococcus↑.* | 1. Inflammatory: IL-6↑;  2. Oxidative stress: MDA↓;  3. BAs: Cecal TBAs↓. | Nie et al. (2019) |
| Glucoside | Gastrodin | AS  C57BL/6J mice | 1. Serum TC, TG, LDL-C↓;  2. Lipid droplet deposition↓. | *-* | 1. Inflammatory: LPS, IL-1β, TNF-α, ICAM-1↓;  2. Intestinal barrier: OCLN, ZO-1, claudin-1↑. | Liu et al. (2021) |
| Oligosaccharides | Chitooligosaccharide (COSM) | NAFLD  C57BL/6 mice | 1. Serum TC, TG, LDL-C, FFA, AST, ALT↓, HDL-C↑, Hepatic TC, TG↓. | *Firmicutes, F/B ratio, Desulfovibrio, Lactococcus↓; Bacteroidetes, Erysipelatoclostridium, Akkermansia↑.* | 1. Oxidative stress: T-AOC↑;  2. Inflammatory: LPS, Serum and hepatic IL-6, TNF-α↓, IL-10↑;  3. SCFAs: Total SCFA, acetic acid, propionic acid↑;  4. Intestinal barrier: ZO-1↑;  5. Signaling pathway: LPS/TLR4/NF-κB↓. | Feng et al. (2022) |
|  | Porphyran-derived oligosaccharides (PYOs) | NAFLD  C57BL/6J mice | 1. Serum TC, TG, FFA, ALT, AST, ALP, γ-GT↓. | *Firmicutes, F/B ratio, Helicobacter, Mucispirillum↓; Parabacteroides, Deferribacteres, Bacteroidetes, Bacteroides, Akkermansia, Alloprevotella, Clostridium XIVa, Barnesiella↑.* | 1. Inflammatory: TNF-α, MCP-1, IL-6↓;  2. Oxidative stress: GSH, CAT, SOD↑, MDA↓;  3. BAs: Serum TBA↓;  4. Signaling pathway: IRS-1/AKT/GSK-3β↑, AMPK↑, TGF-β↓. | Wang et al. (2021) |
| Pentacyclic triterpenoid glycoside | *Diammonium glycyrrhizinate* (DG) | NAFLD | 1. BW↓;  2. Hepatic steatosis↓. | *F/B ratio,* *Desulfovibrio↓;* *Proteobacteria, Lactobacillus,* *Ruminococcaceae, Lachnospiraceae↑.* | 1. Inflammatory: serum LPS, IL-6, TNF-α, IL-1β↓;  2. Intestinal barrier: ZO-1, Occludin, Claudin-1*↑*;  3. SCFAs: Acetate, propionate, isobutyrate, butyrate, isovalerate, valerate↑. | Li et al. (2018) |
| Phenolic acids | Caffeic acid | NAFLD  C57BL/6J mice | 1. BW, liver weight↓;  2. Serum TC, TG, LDL-C, ALT↓;  3. Hepatic steatosis, hepatic lipid accumulation↓. | *Faecalibaculum, Fusobacterium, Ruminococcaceae_UCG-005, Campylobacter↓; Bacteroides, Parasutterella, Bradyrhizobium↑.* | 1. Inflammatory: TNF-α, IL-6↓;  2. Signaling pathway: LPS/TLR4↓. | Mu et al. (2021) |
| Polyphenols | Black tea polyphenols (BTP) | Obesity  C57BL/6J mice | 1. BW↓. | *Firmicutes, Actinobacter, Roseburia, Lactobacillus, Blautia, Anaerostipes, Shuttleworthia, Bryantella, Lactococcus, Acetitomaculum, Collinsella↓;* *Bacteroidetes, Parabacteroides, Bacteroides,* *Prevotella, Oscillibacter, Anaerotruncus, Pseudobutyrivibrio↑.* | 1. SCFAs: Fecal SCFA propionic acid, i-butyric acid↑. | Henning et al. (2018) |
|  | Burdock inulin | NAFLD  C57BL/6J mice | 1. BW, liver weight, liver index↓;  2. Serum TC, TG, LDL-C, AST, ALT↓;  3. Hepatic steatosis↓. | *Firmicutes, F/B* ratio*, Dubosiella, Lactobacillus↓;* *Faecalibaculum↑.* | - | Wang et al. (2020) |
|  | Citrus Peel Powder extract (CPP) | NAFLD  SD rats | 1. BW, liver weight↓;  2. Serum TC, TG, LDL-C, ALT↓, HDL-C↑. | *Campylobacterota, Helicobacter, Blautia, Bacteroidota, Bacteroides↓; Faecalibaculum, Lactobacillus, Dubosiella, Lachnospiraceae_NK4A136_group↑.* | 1. Inflammatory: IL-6, MCP-1, TNF-α, COX-2↓. | Hu et al. (2021) |
|  | Epigallocatechin gallate (EGCG) | NAFLD  C57BL/6J mice | 1. BW↓;  2. Liver TC, TG↓. | *-* | 1. Inflammatory: LPS, TNF-α, IL-6↓. | Li et al. (2020) |
|  |  | NAFLD  SD rats | 1. BW↓;  2. Serum TC, TG, AST, ALT↓. | *Bacteroides, Parasutterella, Treponema_2, Phascolarctobacterinum, Desulfovibrio, Escherichia↓; Lactobacillus, Rauminococcus_1,* *Bifidobacterium, Roseburia, Clostridium, Akkermansia↑.* | 1. Inflammatory: LPS, DAO, IL-1β, IL-6, MCP-1↓;  2. Oxidative stress: SOD, TAC↑, MDA↓;  3. Intestinal barrier: Occludin, ZO-1↑;  4. SCFAs: Total SCFAs, Acetic acid, propionic acid, butyrate acid↑;  5. Signaling pathway: TLR4/MYD88/NF-κB↓. | Zuo et al. (2020) |
|  | Fucoidan | Hyperlipidemia  ICR mice | 1. BW, BMI, Lee index, liver index↓. | *E. faecium R0026↑.* | 1. Inflammatory: LPS, TNF-α↓;  2. BAs: BSH, TBA*↑*. | Huang et al. (2021) |
|  | Honokiol | Obesity  C57BL/6 mice | 1. BW↓;  2. Serum TC, TG, LDL-C, FFA↓. | *Oscillospira, Muribaculaceae, Ruminococcus, Unclassified_Clostridiales↓; Akkermansia, Bacteroides↑.* | 1. Inflammatory: LPS, TNF-α, IL-1β↓. | Ding et al. (2019) |
|  | Inulin | NAFLD  C57BL/6J mice | 1. BW↓;  2. Serum TC, TG, AST, ALT↓;  3. Liver lipid deposition↓. | *Proteobacteria, Blautia↓; Bifidobacterium, Akkermansia, Ileibacterium↑.* | 1. Inflammatory: LPS, TNF-α, IL-1β, IL-6, IL-18，NLRP3, ASC, caspase-1, NF‐κB↓, IL-10↑;  2. SCFAs: Acetic acid, propionic acid, butyrate acid↑. | Bao et al. (2021) |
|  |  | NAFLD  C57/BL-6N mice | 1. BW↓;  2. Serum TC, TG, AST, ALT, Liver TG↓;  3. Hepatic steatosis, Liver lipid deposition↓. | *Firmicutes↓; Verrucomicrobia, Deferribacteres, Parabacteroides, Akkermansia muciniphila↑.* | 1. Intestinal barrier: Occludin, ZO-1↑;  2. Signaling pathway: TLR4↓. | Perez-Monter et al. (2022) |
|  |  | NAFLD  Patients | 1. Serum TC, TG, AST, ALT↓. | *Escherichia coli, Enterococcus↓; Lactobacillus, Bacteroides, Bifidobacterium↑.* | 1. Inflammatory: DAO, D-Lactic acid↓. | Cui et al. (2022) |
|  |  | AS  ApoE^-/-^ mice | 1. Serum TC, TG, LDL-C↓;  2. Vascular fibrosis level↓. | *F/B* ratio*, Alistipes, Intestinimonas, Bilophila, Oscillibacter, Negativibacillus↓; Faecalibaculum↑.* | 1. Inflammatory: LPS, TNF-α, IL-1β, IL-6, IL-17A↓;  2. SCFAs: Acetic acid, propionic acid, valeric acid↑. | Wang et al. (2021) |
|  | Jaboticaba peel and seed powder (JPSP) | NAFLD  C57BL/6J mice | 1. BW↓;  2. Serum TC, TG, LDL-C↓. | *Bifidobacteriaceae, Mogibacteriaceae, Christensenellaceae, Clostridiaceae, Dehalobacteriaceae, Peptococcaceae, Peptostreptococcaceae, Ruminococcaceae↓; Lachnospiraceae, Enterobacteriaceae, Parabacteroides, Sutterella, Allobaculum, Akkermansia↑.* | - | Soares et al. (2021) |
|  | *L. caerulea L. berry* polyphenols (LCBP) | NAFLD  C57BL/6N mice | - | *Firmicutes, F/B* ratio*↓; Bacteroidetes, Proteobacteria, Verrucomicrobia, Akkermansia, Bacteroides↑.* | 1. Inflammatory: LPS, IL-2, TNF-α↓. | Wu et al. (2018) |
|  | Noni fruit polysaccharide (NFP) | NAFLD  C57BL/6J mice | 1. BW↓;  2. Serum TC, TG, LDL-C, ALT↓, HDL-C↑, liver TC, TG↓. | *Prevotella_9, Collinsella, Bacteroides, Turicibacter↓;* *Lactobacillus, Ruminococcaceae_UCG_014, Parasutterella, [Eubacterium]_coprostanoligenes_group, Ruminococcus_1↑.* | 1. Inflammatory: LPS, TNF-α, IL-1β↓, IL-10↑;  2. Intestinal barrier: Occludin, ZO-1, CCL5 mRNA↑;  3. Oxidative stress: MDA↓, TEAC, SOD, GSH-Px, CAT↑;  4. SCFAs: Butyric acid↑, GPR43↓. | Yang et al. (2020) |
|  | polyphenol-rich loquat fruit extract  (LFP) | NAFLD  C57BL/6J mice | 1. BW, liver weight↓;  2. Serum TC, TG, LDL-C↓, HDL-C↑. | *F/B* ratio, *Ruminococcaceae UCG-014, Ruminiclostridium 9, Lachnospiraceae UCG-008, [Eubacterium] f issicatena group, Veillonella, Ruminococcaceae NK4A214 group↓; Bacteroidetes↑.* | 1. Inflammatory: Hepatic LPS, Serum MCP-1, TNF-α↓;  2. Oxidative stress: Liver T-SOD, GSH, GSH-Px↑, MDA↓;  3. Intestinal barrier: Occludin, ZO-l, mucin-2 (MUC-2), MUC-4↑;  4. Signaling pathway: AKT, NF-κB, JNK/Nrf2, TLR4/MyD88/TRIF↓. | Li et al. (2019) |
|  | Proanthocyanidin | Hyperlipidemia  SD rats | 1. Serum TC, TG, LDL-C↓;  2. Serum LCAT↑. | *-* | 1. BAs: Serum TBA↓, Fecal TBA↑. | Fu et al. (2013) |
|  | Resveratrol (RSV) | NAFLD  C57BL/6J mice | 1. BW, Fiver index↓;  2. Serum T-CHO, TG, AST, ALT↓;  3. Hepatic steatosis↓. | *Desulfovibrio↓; Akkermansia muciniphila, Ruminococcaceae, Lachnospiraceae↑.* | 1. Inflammatory: LPS, TNF-α, IL-1β, IL-6↓, IL-10↑;  2. Intestinal barrier: Occludin, ZO-l, Claudinl↑;  3. Signaling pathway: FAK/MyD88/IRAK4↓. | Chen et al. (2019) |
|  |  | NAFLD  C57BL/6 mice | 1. BW, liver index↓;  2. Serum TC, TG, AST, ALT↓;  3. Hepatic steatosis↓. | *Firmicutes, Verrucomicrobia, Proteobacteria↓; Bacteroidetes↑.* | 1. Inflammatory: LPS, TNF-α↓;  2. Intestinal barrier: Occludin mRNA↑. | Yao et al. (2017) |
|  |  | NAFLD  C57BL/6 mice | 1. BW, liver index↓;  2. Hepatic lipid deposition↓. | *Desulfovibrio↓; Akkermansia muciniphila, Ruminococcaceae, Lachnospiraceae↑.* | 1. Inflammatory: FAK, MyD88, IRAK4↓, CB1, CB2 mRNA↓;  2. Intestinal barrier: Occludin, ZO-1, Claudin1↑. | Chen et al. (2020) |
|  |  | AS  ApoE^- /-^ mice | 1. Serum TC↓. | *Firmicutes, Prevotella, Alistipes, Helicobacter↓; Bacteroidetes, Bacteroides, Lactobacillus, Bifidobacterium, Akkermansia↑.* | 1. TMA, TMAO↓;  2. BAs: BSH, TCA/T-βMCA ratio↑, TCA, T-βMCA, CA, CDCA, DCA, CYP7A1, OSTα， OSTβ, conjugated/unconjugated BA ratio↓;  3. Signaling pathway: FXR-FGF15 axis↓. | Chen et al. (2016) |
|  | Tea polyphenol (TP) | Obesity  human flora-associated C57BL/6J mice | 1. Serum TC, TG, LDL-C↓. | *F/B* ratio*↓.* | 1. SCFAs: Acetic acid and butyric acid↑. | Wang et al. (2018) |
|  | Theabrownins | Hyperlipidemia  C57BL/6J mice | 1. BW, iWAT weight, eWAT weight, pWAT weight↓;  2. Serum TC, TG↓. | *Lactobacillus, Bacillus, Streptococcu, Lactococcus↓.* | 1. BAs: BSH↓, Serum TCDCA, TUDCA, Fecal TBA↑.  2. Signaling pathway: FXR-FGF15 axis↓, CYP7B1↑. | Huang et al. (2019) |
| Polysaccharides | *Astragalus mongholicus* polysaccharides (mAPS) | NAFLD  SD rats | 1. BW↓;  2. Serum TC, TG, LDL-C, ALT, AST↓, HDL-C↑. | *F/B ratio↓; Proteobacteria, Epsilonbacteria↑.* | 1. Inflammatory: TNF-α↓;  2. Intestinal barrier: ZO-1, Occludin↑;  3. Signaling pathway: TLR4/NF-κB↓, AMPK, PPAR-α↑, SREBP-1↓, GPR41, GPR43↓. | Zhong et al. (2022) |
|  | Ganoderma lucidum polysaccharide (GLP) | Obesity  C57BL/6J mice | 1. BW↓;  2. Serum LDL-C and NEFA↓. | *F/B ratio, Lachnospiraceae_UCG-001, Lachnospiraceae_FCS020_group, Ruminiclostrdium, Riminococcaceae_ UCG-009, Firmicutes_bacterium_ASF500, Alistipes_sp._627, Clostridium_leptum↓; Allobaculum, Bifidobacterium, Christensenellaceae_R-7_group↑.* | 1. SCFAs: Acetate and butyrate↑, GPR43 expression↑;  2. Inflammatory: Serum LPS, TNF-α, IL-1β, MCP-1↓, eWAT IL-6, IL-1β, MCP-1, LBP, CD14, CD11b, CD11c, CD68↓;  3. Intestinal barrier: ZO-1, Occludin, Claudin-1, Lyz1, Reg3γ*↑*;  4. Signaling pathway: TLR4/Myd88/NF-κB pathway↓. | Sang et al. (2021) |
|  | Ganoderma lucidum polysaccharide and chitosan (PC) | Hyperlipidemia  Syrian golden hamsters | 1. Serum TC, TG, LDL-C↓, HDL-C/LDL-C↑;  2. Serum AST, AST/ALT↓; | *Intestinimonas, Pseudoflavonifractor, Schwartzia, Desulfovermiculus, Desulfovibrio, Clostridium IV, Clostridium XIVa↓; Ruminococcus, Oscillibacter, Bifidobacterium, Prevotella, Alloprevotella, Paraprevotella, Alistipes↑.* | - | Tong et al. (2020) |
|  | *Laminaria japonica* polysaccharide (LJP) | NAFLD  C57BL/6 mice | 1. BW↓;  2. Serum TC, TG, LDL-C, ALT, AST↓, Hepatic TG↓;  3. Hepatic steatosis↓. | *Firmicutes, F/B ratio, Verrucomicrobia, Epsllonbacteraeota, Intestinimonas, Blautia↓; Bacteroides, Akkermansia↑.* | 1. SCFAs: Total SCFAs, propionate↑. | Zhang et al. (2021) |
|  | *Lycium barbarum* polysaccharide (LBPs) | NAFLD  SD rats | 1. BW, Lee’s index↓;  2. Serum TC, TG, LDL-C, FFA, leptin, AST/ALT↓, HDL-C↑, Hepatic TC, TG↓;  3. Hepatic steatosis↓. | *F/B ratio, Verrucomicrobia, Enterococcaceae↓; Deferribacteres, Deferribacteracea↑.* | 1. Inflammatory: LPS, IL-6, IL-1β, TNF-α, MCP-1↓, IL-10↑;  2. SCFAs: Fecal acetic, butyric, valeric acid↑;  3. Intestinal barrier: ZO-1, Occludin↑;  4. Signaling pathway: LPS/TLR4/NF-κB↓. | Gao et al. (2021) |
|  | Porphyran | Hyperlipidemia  C57BL/6J mice | 1. BW, liver index↓;  2. Serum TC, TG, LDL-C, FFA↓, HDL-C↑. | *Firmicutes, Helicobacter↓; Bacteroidetes, Proteobacteria, Verrucomicrobia, Bacteroides, Alistipes↑.* | 1. Signaling pathway: AMPK-HSL/ACC in WAT↑, fat accumulation in WAT↓; UCP1/PGC1α in BAT↑, white-to-brown fat conversion↑. | Wang et al. (2022) |
|  | Procyanidin B2 (PB2) | NAFLD  New Zealand white rabbits | 1. BW, liver index↓;  2. Serum TC, TG, LDL-C↓, HDL-C↑, Hepatic TG↓;  3. Hepatic steatosis↓. | *F/B ratio↓; Bacteroidetes, Akkermansia↑.* | 1. Inflammatory: Serum LPS↓. | Xing et al. (2019) |
|  | Pueraria lobata starch (PLS) | NAFLD  C57BL/6J mice | 1. Serum TC, TG, LDL-C↓;  2. Hepatic steatosis↓. | *Desulfovibrio↓; Lactobacillus, Bifidobacterium, Turicibacter↑.* | - | Yang et al. (2022) |
|  | Purple yam (*Dioscorea alata L.*) resistant starch (PYRS) | Hyperlipidemia  Hamsters | 1. Serum TC, TG, LDL-C↓, HDL-C↑. | *Parabacteroides, Dorea↓; Bifidobacteria, Lactobacillus, Coprococcus, Allobaculum↑.* | - | Li et al. (2019) |
|  | Resistant starch (RS) | NAFLD  C57BL/6J mice | 1. BW, liver weight, liver index↓;  2. Serum TC, TG, LDL-C, ALT↓;  3. Hepatic steatosis↓. | *F/B ratio↓; Akkermansia↑.* | - | Shou et al. (2021) |
|  | *Rosa Laevigata Michx. Fruits* Polysaccharides (RLPs) | NAFLD  C57BL/6J mice | 1. BW↓;  2. Serum TC, TG, LDL-C, ALT, AST, γ-GGT, FFA↓, HDL-C↑. | *F/B ratio, Firmicutes, Blautia, Dorea, Roseburia↓; Bacteroidetes, Alistipes, Butyricimonas, Prevotella↑.* | 1. SCFAs: Propionic acid, butyric acid, isovaleric acid, valeric acid↑;  2. Oxidative stress: SOD, GSH-Px, CAT↑;  3. Inflammatory: LPS, TNF-α, IL-1β, IL-6, MCP-1↓. | Zhang et al. (2020) |
|  | *Rosa roxburghii Tratt* polysaccharide (RTFP) | NAFLD  C57BL/6J mice | 1. Liver TC, TG↓. | *F/B ratio, Lachnospiraceae, Helicobacteraceae, Coriobacteriaceae_UCG-002, Erysipelotrichaceae↓; Akkermansia↑.* | 1. Oxidative stress: T-AOC, T-SOD, GSH-PX↑, MDA↓;  2. Intestinal barrier: Claudin-1, ZO-1, Occludin mRNA↑;  3. Inflammatory: IL-6, IL-1β, TNF-α↓. | Zhang et al. (2022) |
|  | water insoluble polysaccharide (WIP) from the sclerotium of *Poria cocos (Schw.) Wolf* [Polyporaceae; *Poria*] | Hyperlipidemia  ob/ob mice | 1. Serum TC, TG, LDL-C, ALT, AST↓;  2. Adipocytes hypertrophy in WAT↓. | *Megamonas, Proteus↓; Bacteroidetes,* *Lachnospiracea,* *Clostridium, Alloprevotella, Parabacteroides,* *Ruminococcus↑.* | 1. SCFAs: butyrate↑;  2. Intestinal barrier: Muc-5, ZO-1, Occludin↑;  3. Inflammatory: LPS, TNF-α↓;  4. Oxidative stress: SOD↑;  5. Signaling pathway: PPAR-γ pathway↑. | Sun et al. (2019) |
| Triterpenoid saponins | *Gynostemma pentaphyllum* saponins (GPS) | NAFLD  SD rats | 1. BW, Liver weight↓;  2. Serum TC, TG, LDL-C, ALT, AST↓, HDL-C↑, Liver TC, TG↓;  3. Hepatocyte fat vacuoles, lipid droplet deposition↓. | *Firmicutes, Firmicutes/Bacteroidetes (F/B) ratio, Desulfovibrio, Escherichia-Shigella, Helicobacter↓; Bacteroidetes, Akkermansia, Bacteroides, Parabacteroides↑.* | 1. Oxidative stress: MDA↓, SOD, CAT↑;  2. Inflammatory: IL-6↓;  3. SCFAs: Acetic acids, propionic acids, butyric acids↑. | Zhong et al. (2022) |
|  | *Ilex pubescens* triterpenoid saponins (IPTS) | AS  SD rats | 1. Serum TC, TG, LDL-C↓, HDL-C↑. | *-* | 1. SCFAs: Fecal butyrate, valerate↑, formate, acetate↓;  2. TMAO in urine↓;  3. Amino acids metabolism: 4-Aminobutyrate, Indole-3-acetate, Kynurenine↑. | Bai et al. (2022) |
|  |  | AS  SD rats | 1. Serum TC, TG, LDL-C↓, HDL-C↑;  2. Atherosclerotic plaque area↓. | *Verrucomicrobia, Proteobacteria, Bacteroidetes, Desulfobacteraceae, Roseburia, Lachnospira, Desulfobulbus, Desulfovibrio, Akkermansia↓; Firmicutes, Sphingobacterium, Lactobacillus, Blautia↑.* | - | Bai et al. (2021) |
|  | Tea seed saponins | Hyperlipidemia  Wistar rats | - | *Desulfovibrionaceae, Porphyromonadaceae↓; Lachnospiraceae, Ruminococcaceae, Roseburia, Eubacterium_coprostanoligenes_group, Lactobacillus↑.* | - | Lin et al. (2020) |
