# Supplementary material for: Intestinal flora: A new target for traditional Chinese medicine to improve lipid metabolism disorders
Source: Front Pharmacol. 2023 Mar 1;14:1134430. doi: 10.3389/fphar.2023.1134430 (PMC10014879; doi:10.3389/fphar.2023.1134430)
Supplement: Supplementary file 2 [file Table2.docx]

Supplementary table 2 The composition of Chinese herbal formulas and proprietary Chinese medicines.

| Prescription | Common composition | Proportion | References |
| --- | --- | --- | --- |
| Chinese herbal formulas | | | |
| Alisma orientalis Beverage (AOB) | *Alisma plantago-aquatica subsp. orientale (Sam.) Sam.* [Alismataceae; *Alismatis rhizoma*], *Atractylodes macrocephala Koidz.* [Asteraceae; *Atractylodis macrocephalae rhizoma*], *Pyrola forrestiana Andres* [Ericaceae; *Pyrolae herba*]  Prepared by Zhu et al. (2021) according to China Pharmacopoeia | 20:20:10 | Zhu et al. (2021) |
| Biejia Jian Wan  (BJJW) | *Trionyx sinensis Wiegmann* [Trionychidae; *Trionycis carapax*], *Ejiao*, *Polistesolivaceous (DeGeer)* [Vespidae; *Vespaenidus*], *Shufuchong*, *Eupolyphaga sinensis Walker* [Corydidae; *Eupolyphaga steleophaga*], *Qianglang*, *Xiaoshi*，*Bupleurum chinense DC.* [Apiaceae; *Bupleuri radix*], *Scutellaria baicalensis Georgi* [Lamiaceae; *Scutellariae radix*], *Pinellia ternata (Thunb.) Makino* [Araceae; *Pinelliae rhizoma praeparatum*], *Codonopsis pilosula (Franch.) Nannf.* [Campanulaceae; *Codonopsis radix*], *Zingiber officinale Roscoe* [Zingiberaceae; *Zingiberis rhizoma praeparatum*], *Magnolia officinalis Rehder & E.H.Wilson* [Magnoliaceae; *Magnoliae officinalis cortex*], *Neolitsea cassia (L.) Kosterm.* [Lauraceae; *Cinnamomi ramulus*], *Paeonia lactiflora Pall.* [Paeoniaceae; *Paeoniae radix alba*], *Iris domestica (L.) Goldblatt & Mabb.* [Iridaceae; *Belamcandae rhizoma*], *Prunus persica (L.) Batsch* [Rosaceae; *Persicae semen*], *Paeonia × suffruticosa Andrews* [Paeoniaceae; *Moutan cortex*], *Rheum palmatum L.* [Polygonaceae; *Rhei radix et rhizoma*], *Campsis grandiflora (Thunb.) K.Schum.* [Bignoniaceae; *Campsis flos*], *Descurainia sophia (L.) Webb ex Prantl* [Brassicaceae; *Descurainiae semen, lepidii semen*], *Pyrrosia lingua (Thunb.) Farw.* [Polypodiaceae; *Pyrrosiae folium*], *Dianthus superbus L.* [Caryophyllaceae; *Dianthi herba*]  Prepared by Qiu et al. (2017) according to China Pharmacopoeia | 18:30:40:30:50:60:120:60:30:10:10:30:30:30:50:30:20:50:30:20:50:30:30:10:30:20 | Qiu et al. (2017) |
| Cangju Qinggan Formula (CJQG) | *Atractylodes lancea (Thunb.) DC.* [Asteraceae; *Atractylodis rhizoma*], *Atractylodes macrocephala Koidz.* [Asteraceae; *Atractylodis macrocephalae rhizoma*], *Typha angustifolia L.* [Typhaceae; *Typhae pollen*], *Chrysanthemum × morifolium (Ramat.) Hemsl.* [Asteraceae; *Chrysanthemi flos*], Senna tora (L.) Roxb. [Fabaceae; Cassiae semen], *Nelumbo nucifera Gaertn.* [Nelumbonaceae; *Nelumbinis folium*]  Prepared by Ling et al. (2022) according to China Pharmacopoeia | 6:12:6:9:9:9 | Ling et al. (2022) |
| Chaihu Shugan San (CHSG) | *Bupleurum chinense DC.* [Apiaceae; *Bupleuri radix*], Citrus × aurantium L. [Rutaceae; *Aurantii fructus immaturus*], *Conioselinum anthriscoides 'Chuanxiong'* [Apiaceae; *Chuanxiong rhizoma*], *Cyperus rotundus L.* [Cyperaceae; *Cyperi rhizoma*], *Citrus × aurantium L.* [Rutaceae; *Aurantii fructus*], *Paeonia lactiflora Pall.* [Paeoniaceae; *Paeoniae radix alba*], *Glycyrrhiza glabra L.* [Fabaceae; *Glycyrrhizae radix et rhizoma*]  Prepared by Xie et al. (2021) according to China Pharmacopoeia | 15:10:15:15:15:15:10 | Xie et al. (2021) |
| Danggui Shaoyao San (DGSY) | *Angelica sinensis (Oliv.) Diels* [Apiaceae; *Angelicae sinensis radix*], *Paeonia lactiflora Pall.* [Paeoniaceae; *Paeoniae radix alba*], *Paeonia lactiflora Pall.* [Paeoniaceae; *Paeoniae radix rubra*], *Conioselinum anthriscoides 'Chuanxiong'* [Apiaceae; *Chuanxiong rhizoma*], *Alisma plantago-aquatica subsp. orientale (Sam.) Sam.* [Alismataceae; *Alismatis rhizoma*], *Atractylodes macrocephala Koidz.* [Asteraceae; *Atractylodis macrocephalae rhizoma*], *Poria cocos (Schw.) Wolf* [Polyporaceae; *Poria*]  Prepared by Yu et al. (2021) according to China Pharmacopoeia | 10:24:24:24:24:12:12 | Yu et al. (2021) |
| Dingxin Recipe IV (DXR) | *Coptis chinensis Franch.* [Ranunculaceae; *Coptidis rhizoma*], *Salvia miltiorrhiza Bunge* [Lamiaceae; *Salviae miltiorrhizae radix et rhizoma*], *Ziziphus jujuba Mill.* [Rhamnaceae; *Jujubae fructus*], *Ganoderma lucidum (Leyss.ex Fr.) Karst.* [Polyporaceae; *Ganoderma*]  Prepared by Zhang et al. (2021) according to China Pharmacopoeia | 15:28:38:19 | Zhang et al. (2021) |
| Erchen Decoction (ECD) | Citrus × aurantium L. [Rutaceae; *Aurantii fructus immaturus*], *Pinellia ternata (Thunb.) Makino* [Araceae; *Pinelliae rhizoma praeparatum*], *Poria cocos (Schw.) Wolf* [Polyporaceae; *Poria*], *Glycyrrhiza glabra L.* [Fabaceae; *Glycyrrhizae radix et rhizoma*]  Prepared by Liu et al. (2021) according to China Pharmacopoeia | 15:15:9:4.5 | Liu et al. (2021) |
| Gegen Qinlian Decoction (GGQL) | *Pueraria montana var. lobata (Willd.) Maesen & S.M.Almeida ex Sanjappa & Predeep* [Fabaceae; *Puerariae lobatae radix*], *Scutellaria baicalensis Georgi* [Lamiaceae; *Scutellariae radix*], *Coptis chinensis Franch.* [Ranunculaceae; *Coptidis rhizoma*], *Glycyrrhiza glabra L.* [Fabaceae; *Glycyrrhizae radix et rhizoma*]  Prepared by Jiang et al. (2020) according to China Pharmacopoeia | 8:3:3:2 | Jiang et al. (2020) |
| Gegen Qinlian Decoction (GQD) | *Pueraria montana var. lobata (Willd.) Maesen & S.M.Almeida ex Sanjappa & Predeep* [Fabaceae; *Puerariae lobatae radix*], *Scutellaria baicalensis Georgi* [Lamiaceae; *Scutellariae radix*], *Coptis chinensis Franch.* [Ranunculaceae; *Coptidis rhizoma*], *Glycyrrhiza glabra L.* [Fabaceae; *Glycyrrhizae radix et rhizoma*]  Prepared by Liu et al. (2019) according to China Pharmacopoeia | 8:3:3:2 | Liu et al. (2019) |
| Guizhi Tang (GZT) | *Neolitsea cassia (L.) Kosterm.* [Lauraceae; *Cinnamomi ramulus*], *Paeonia lactiflora Pall.* [Paeoniaceae; *Paeoniae radix alba*], *Glycyrrhiza glabra L.* [Fabaceae; *Glycyrrhizae radix et rhizoma*], *Zingiber officinale Roscoe* [Zingiberaceae; *Zingiberis rhizoma recens*], *Ziziphus jujuba Mill.* [Rhamnaceae; *Jujubae fructus*]  Prepared by Yuan et al. (2021) according to China Pharmacopoeia | 10:10:7:10:10 | Yuan et al. (2021) |
| Huanglian Jiedu Decoction (HLJD) | *Coptis chinensis Franch.* [Ranunculaceae; *Coptidis rhizoma*], *Scutellaria baicalensis Georgi* [Lamiaceae; *Scutellariae radix*], *Phellodendron chinense C.K.Schneid.* [Rutaceae; *Phellodendri chinensis cortex*], *Gardenia jasminoides J.Ellis* [Rubiaceae; *Gardeniae fructus*]  Prepared by Jiang et al. (2021) according to China Pharmacopoeia | 9:6:6:9 | Jiang et al. (2021) |
| Huangqin Decoction (HQD) | *Scutellaria baicalensis Georgi* [Lamiaceae; *Scutellariae radix*], *Paeonia lactiflora Pall.* [Paeoniaceae; *Paeoniae radix alba*], *Glycyrrhiza glabra L.* [Fabaceae; *Glycyrrhizae radix et rhizoma*], *Ziziphus jujuba Mill.* [Rhamnaceae; *Jujubae fructus*]  Prepared by Yan et al. (2022) according to China Pharmacopoeia | 3:2:2:2 | Yan et al. (2022) |
| Huayu Qutan Formula (HYQT) | *Gynostemma pentaphyllum (Thunb.) Makino* [Cucurbitaceae; *Gynostemmatis herba*], *Codonopsis pilosula (Franch.) Nannf.* [Campanulaceae; *Codonopsis radix*], *Poria cocos (Schw.) Wolf* [Polyporaceae; *Poria*], *Astragalus mongholicus Bunge* [Fabaceae; *Astragali radix*], *Salvia miltiorrhiza Bunge* [Lamiaceae; *Salviae miltiorrhizae radix et rhizoma*], *Pinellia ternata (Thunb.) Makino* [Araceae; *Pinelliae rhizoma praeparatum*], *Conioselinum anthriscoides 'Chuanxiong'* [Apiaceae; *Chuanxiong rhizoma*], *Curcuma aromatica Salisb.* [Zingiberaceae; *Curcumae rhizoma*], *Acorus gramineus Aiton* [Acoraceae; *Acori gramineri rhizoma*]  Prepared by Sui et al. (2021a) according to China Pharmacopoeia | 30:30:15:30:15:15:20:15:20 | Sui et al. (2021a) |
| Huayu Qutan Formula (HYQT) | *Gynostemma pentaphyllum (Thunb.) Makino* [Cucurbitaceae; *Gynostemmatis herba*], *Codonopsis pilosula (Franch.) Nannf.* [Campanulaceae; *Codonopsis radix*], *Poria cocos (Schw.) Wolf* [Polyporaceae; *Poria*], *Astragalus mongholicus Bunge* [Fabaceae; *Astragali radix*], *Salvia miltiorrhiza Bunge* [Lamiaceae; *Salviae miltiorrhizae radix et rhizoma*], *Pinellia ternata (Thunb.) Makino* [Araceae; *Pinelliae rhizoma praeparatum*], *Conioselinum anthriscoides 'Chuanxiong'* [Apiaceae; *Chuanxiong rhizoma*], *Curcuma aromatica Salisb.* [Zingiberaceae; *Curcumae rhizoma*], *Acorus gramineus Aiton* [Acoraceae; *Acori gramineri rhizoma*]  Prepared by Sui et al. (2021b) according to China Pharmacopoeia | 30:30:15:30:15:15:20:15:20 | Sui et al. (2021b) |
| Huayu Qutan Formula (HYQT) | *Gynostemma pentaphyllum (Thunb.) Makino* [Cucurbitaceae; *Gynostemmatis herba*], *Codonopsis pilosula (Franch.) Nannf.* [Campanulaceae; *Codonopsis radix*], *Poria cocos (Schw.) Wolf* [Polyporaceae; *Poria*], *Astragalus mongholicus Bunge* [Fabaceae; *Astragali radix*], *Salvia miltiorrhiza Bunge* [Lamiaceae; *Salviae miltiorrhizae radix et rhizoma*], *Pinellia ternata (Thunb.) Makino* [Araceae; *Pinelliae rhizoma praeparatum*], *Conioselinum anthriscoides 'Chuanxiong'* [Apiaceae; *Chuanxiong rhizoma*], *Curcuma aromatica Salisb.* [Zingiberaceae; *Curcumae rhizoma*], *Acorus gramineus Aiton* [Acoraceae; *Acori gramineri rhizoma*]  Prepared by Zheng et al. (2022) according to China Pharmacopoeia | 30:30:15:30:15:15:20:15:20 | Zheng et al. (2022) |
| Huazhi-Rougan Formula (HZRG) | *Artemisia capillaris Thunb.* [Asteraceae; *Artemisiae scopariae herba*], *Senna tora (L.) Roxb.* [Fabaceae; *Cassiae semen*], *Rheum palmatum L.* [Polygonaceae; *Rhei radix et rhizoma*], *Alisma plantago-aquatica subsp. orientale (Sam.) Sam.* [Alismataceae; *Alismatis rhizoma*], *Polyporus umbellatus (Pers.) Fries* [Polyporaceae; *Polyporus*], *Crataegus pinnatifida Bunge* [Rosaceae; *Crataegi fructus*], *Atractylodes lancea (Thunb.) DC.* [Asteraceae; *Atractylodis rhizoma*], *Citrus × aurantium L.* [Rutaceae; *Aurantii fructus immaturus*], *Trichosanthes kirilowii Maxim.* [Cucurbitaceae; *Trichosanthis fructus*], *Ligustrum lucidum W.T.Aiton* [Oleaceae; *Ligustri lucidi fructus*], *Eclipta prostrata (L.) L.* [Asteraceae; *Ecliptae herba*], *Lycium barbarum L.* [Solanaceae; *Lycii fructus*], *Cirsium arvense var. arvense* [Asteraceae; *Cirsii herba*], *Bupleurum chinense DC.* [Apiaceae; *Bupleuri radix*], *Glycyrrhiza glabra L.* [Fabaceae; *Glycyrrhizae radix et rhizoma*]  purchased from Shandong New Time Pharmaceutical CO., Ltd. | NA | Li et al. (2022) |
| Jian Pi Tiao Gan Yin (JPTGY) | *Astragalus mongholicus Bunge* [Fabaceae; *Astragali radix*], *Bupleurum chinense DC.* [Apiaceae; *Bupleuri radix*], *Poria cocos (Schw.) Wolf* [Polyporaceae; *Poria*], *Salvia miltiorrhiza Bunge* [Lamiaceae; *Salviae miltiorrhizae radix et rhizoma*], *Coix lacryma-jobi var. ma-yuen (Rom.Caill.) Stapf* [Poaceae; *Coicis semen*], *Paeonia lactiflora Pall.* [Paeoniaceae; *Paeoniae radix alba*], Senna tora (L.) Roxb. [Fabaceae; Cassiae semen], *Eupatorium fortunei Turcz.* [Asteraceae; *Eupatorii herba*], *Alisma plantago-aquatica subsp. orientale (Sam.) Sam.* [Alismataceae; *Alismatis rhizoma*], *Rheum palmatum L.* [Polygonaceae; *Rhei radix et rhizoma*], *Crataegus pinnatifida Bunge* [Rosaceae; *Crataegi fructus*]  Prepared by Dong et al. (2022) according to China Pharmacopoeia | NA | Dong et al. (2022) |
| Jiangzhi Granules (JZG) | *Gynostemma pentaphyllum (Thunb.) Makino* [Cucurbitaceae; *Gynostemmatis herba*], *Reynoutria japonica Houtt.* [Polygonaceae; *Polygoni cuspidati rhizoma et radix*], *Nelumbo nucifera Gaertn.* [Nelumbonaceae; *Nelumbinis folium*], *Salvia miltiorrhiza Bunge* [Lamiaceae; *Salviae miltiorrhizae radix et rhizoma*]  Prepared by Wang et al. (2021) according to China Pharmacopoeia | 15:15:6:9:9 | Wang et al. (2021) |
| Jiangzhi Ligan Decoction (JZLG) | *Alisma plantago-aquatica subsp. orientale (Sam.) Sam.* [Alismataceae; *Alismatis rhizoma*], Senna tora (L.) Roxb. [Fabaceae; Cassiae semen], *Salvia miltiorrhiza Bunge* [Lamiaceae; *Salviae miltiorrhizae radix et rhizoma*], *Curcuma aromatica Salisb.* [Zingiberaceae; *Curcumae rhizoma*], *Sargassum pallidum (Turn.) C.Ag.* [Sargassaceae; *Sargassum*], *Nelumbo nucifera Gaertn.* [Nelumbonaceae; *Nelumbinis folium*]  Prepared by Tang et al. (2016) according to China Pharmacopoeia | 10:30:10:10:10:30:10 | Tang et al. (2016) |
| Jianpi Huazhuo Tiaozhi Granule (JPHZTZ) | *Codonopsis pilosula (Franch.) Nannf.* [Campanulaceae; *Codonopsis radix*], *Nelumbo nucifera Gaertn.* [Nelumbonaceae; *Nelumbinis folium*], *Poria cocos (Schw.) Wolf* [Polyporaceae; *Poria*], *Atractylodes macrocephala Koidz.* [Asteraceae; *Atractylodis macrocephalae rhizoma*], Citrus × aurantium L. [Rutaceae; *Aurantii fructus immaturus*], *Coix lacryma-jobi var. ma-yuen (Rom.Caill.) Stapf* [Poaceae; *Coicis semen*], *Alisma plantago-aquatica subsp. orientale (Sam.) Sam.* [Alismataceae; *Alismatis rhizoma*], *Cucumis melo L.* [Cucurbitaceae; *Luffae fructus retinervus*]  Prepared by Huang et al. (2021) according to China Pharmacopoeia | 15:10:15:10:10:20:10:10 | Huang et al. (2021) |
| Liqi Huatan Quyu Decoction (LQHTQY) | *Prunus persica (L.) Batsch* [Rosaceae; *Persicae semen*], *Crataegus pinnatifida Bunge* [Rosaceae; *Crataegi fructus*], *Curcuma aromatica Salisb.* [Zingiberaceae; *Curcumae rhizoma*], *Alisma plantago-aquatica subsp. orientale (Sam.) Sam.* [Alismataceae; *Alismatis rhizoma*], *Sargassum pallidum (Turn.) C.Ag.* [Sargassaceae; *Sargassum*], *Fritillaria thunbergii Miq.* [Liliaceae; *Fritillariae thunbergii bulbus*], Citrus medica L. [Rutaceae; *Citri fructus*], *Raphanus raphanistrum subsp. sativus (L.) Domin* [Brassicaceae; *Raphani semen*], *Poria cocos (Schw.) Wolf* [Polyporaceae; *Poria*]  Prepared by Ding et al. (2019) according to China Pharmacopoeia | NA | Ding et al. (2019) |
| Modfied Yinchen Wuling San (MYCWL) | *Artemisia capillaris Thunb.* [Asteraceae; *Artemisiae scopariae herba*], *Poria cocos (Schw.) Wolf* [Polyporaceae; *Poria*], *Alisma plantago-aquatica subsp. orientale (Sam.) Sam.* [Alismataceae; *Alismatis rhizoma*], *Polyporus umbellatus (Pers.) Fries* [Polyporaceae; *Polyporus*], *Neolitsea cassia (L.) Kosterm.* [Lauraceae; *Cinnamomi ramulus*], *Atractylodes macrocephala Koidz.* [Asteraceae; *Atractylodis macrocephalae rhizoma*], *Coix lacryma-jobi var. ma-yuen (Rom.Caill.) Stapf* [Poaceae; *Coicis semen*], Senna tora (L.) Roxb. [Fabaceae; Cassiae semen], *Bupleurum chinense DC.* [Apiaceae; *Bupleuri radix*], *Paeonia lactiflora Pall.* [Paeoniaceae; *Paeoniae radix alba*], *Crataegus pinnatifida Bunge* [Rosaceae; *Crataegi fructus*], *Nelumbo nucifera Gaertn.* [Nelumbonaceae; *Nelumbinis folium*], *Curcuma aromatica Salisb.* [Zingiberaceae; *Curcumae rhizoma*], *Citrus × aurantium L.* [Rutaceae; *Aurantii fructus immaturus*]  Prepared by Xu et al. (2019) according to China Pharmacopoeia | 20:20:15:10:10:15:30:20:15:15:15:20:10:12 | Xu et al. (2019) |
| Modified Xiongdan yinchen Granules (MXYG) | *Xiongdanfen*, *Artemisia capillaris Thunb.* [Asteraceae; *Artemisiae scopariae herba*], *Crataegus pinnatifida Bunge* [Rosaceae; *Crataegi fructus*], *Alisma plantago-aquatica subsp. orientale (Sam.) Sam.* [Alismataceae; *Alismatis rhizoma*], *Atractylodes macrocephala Koidz.* [Asteraceae; *Atractylodis macrocephalae rhizoma*], *Bupleurum chinense DC.* [Apiaceae; *Bupleuri radix*], *Glycyrrhiza glabra L.* [Fabaceae; *Glycyrrhizae radix et rhizoma*]  Prepared by the Department of Pharmacy, the Second People’s Hospital of Fujian University of Traditional Chinese Medicine | NA | Wu et al. (2021) |
| Qiang Gan Formula extract (QGE) | *Artemisia capillaris Thunb.* [Asteraceae; *Artemisiae scopariae herba*], *Strobilanthes cusia (Nees) Kuntze* [Acanthaceae; *Baphicacanthis cusiae rhizoma et radix*], *Angelica sinensis (Oliv.) Diels* [Apiaceae; *Angelicae sinensis radix*], *Paeonia lactiflora Pall.* [Paeoniaceae; *Paeoniae radix alba*], *Salvia miltiorrhiza Bunge* [Lamiaceae; *Salviae miltiorrhizae radix et rhizoma*], *Curcuma aromatica Salisb.* [Zingiberaceae; *Curcumae rhizoma*], *Astragalus mongholicus Bunge* [Fabaceae; *Astragali radix*], *Codonopsis pilosula (Franch.) Nannf.* [Campanulaceae; *Codonopsis radix*], *Alisma plantago-aquatica subsp. orientale (Sam.) Sam.* [Alismataceae; *Alismatis rhizoma*], *Polygonatum cyrtonema Hua* [Asparagaceae; *Polygonati rhizoma*], *Rehmannia glutinosa (Gaertn.) DC.* [Orobanchaceae; *Rehmanniae radix*], *Dioscorea oppositifolia L.* [Dioscoreaceae; *Dioscoreae rhizoma*], *Crataegus pinnatifida Bunge* [Rosaceae; *Crataegi fructus*], *Shenqu*, *Gentiana macrophylla Pall.* [Gentianaceae; *Gentianae macrophyllae radix*], *Glycyrrhiza glabra L.* [Fabaceae; *Glycyrrhizae radix et rhizoma*]  Prepared by Li et al. (2020) according to China Pharmacopoeia | 10:5:5:5:10:5:10:5:5:5:5:5:4:4:4:4 | Li et al. (2020) |
| Qinggan Qushi Huoxie Prescription (QGQSHX) | *Prunella vulgaris L.* [Lamiaceae; *Prunellae spica*], *Buddleja officinalis Maxim.* [Scrophulariaceae; *Buddlejae flos*], *Celosia argentea L.* [Amaranthaceae; *Celosiae semen*], *Artemisia capillaris Thunb.* [Asteraceae; *Artemisiae scopariae herba*], *Sedum sarmentosum Bunge* [Crassulaceae; *Sedi herba*], *Salvia miltiorrhiza Bunge* [Lamiaceae; *Salviae miltiorrhizae radix et rhizoma*], *Curcuma aromatica Salisb.* [Zingiberaceae; *Curcumae rhizoma*]  Prepared by Zhang et al. (2022) according to China Pharmacopoeia | 10:10:10:10:10:15:10 | Zhang et al. (2022) |
| Qinghua Fang (QHF) | *Sedum sarmentosum Bunge* [Crassulaceae; *Sedi herba*], *Salvia miltiorrhiza Bunge* [Lamiaceae; *Salviae miltiorrhizae radix et rhizoma*], *Atractylodes macrocephala Koidz.* [Asteraceae; *Atractylodis macrocephalae rhizoma*], Citrus × aurantium L. [Rutaceae; *Aurantii fructus immaturus*], *Nelumbo nucifera Gaertn.* [Nelumbonaceae; *Nelumbinis folium*], *Gynostemma pentaphyllum (Thunb.) Makino* [Cucurbitaceae; *Gynostemmatis herba*], *Poria cocos (Schw.) Wolf* [Polyporaceae; *Poria*], *Eupatorium fortunei Turcz.* [Asteraceae; *Eupatorii herba*], *Alisma plantago-aquatica subsp. orientale (Sam.) Sam.* [Alismataceae; *Alismatis rhizoma*], *Yumixu*  Prepared by Wang et al. (2021) according to China Pharmacopoeia | 15:9:12:9:15:9:9:15:15:9 | Wang et al. (2021) |
| Qingxin Jieyu Granule (QXJY) | *Astragalus mongholicus Bunge* [Fabaceae; *Astragali radix*], *Salvia miltiorrhiza Bunge* [Lamiaceae; *Salviae miltiorrhizae radix et rhizoma*], *Conioselinum anthriscoides 'Chuanxiong'* [Apiaceae; *Chuanxiong rhizoma*], *Pogostemon cablin (Blanco) Benth.* [Lamiaceae; *Pogostemonis herba*], *Coptis chinensis Franch.* [Ranunculaceae; *Coptidis rhizoma*]  Purchased from Shenzhen China Resources Sanjiu Pharmaceutical Trading Co., Ltd | 15:15:10:10:5 | Wang et al. (2019) |
| Qutan Huazhuo Prescription (QTHZ) | *Cyperus rotundus L.* [Cyperaceae; *Cyperi rhizoma*], Citrus × aurantium L. [Rutaceae; *Aurantii fructus immaturus*], *Pinellia ternata (Thunb.) Makino* [Araceae; *Pinelliae rhizoma praeparatum*], *Poria cocos (Schw.) Wolf* [Polyporaceae; *Poria*], *Atractylodes lancea (Thunb.) DC.* [Asteraceae; *Atractylodis rhizoma*], *Wurfbainia villosa (Lour.) Skornick. & A.D.Poulsen* [Zingiberaceae; *Amomi fructus*], *Shenqu*, *Crataegus pinnatifida Bunge* [Rosaceae; *Crataegi fructus*], *Gardenia jasminoides J.Ellis* [Rubiaceae; *Gardeniae fructus*], *Conioselinum anthriscoides 'Chuanxiong'* [Apiaceae; *Chuanxiong rhizoma*]  Prepared by Li et al. (2021) according to China Pharmacopoeia | 10:8:9:10:10:5:15:15:10:9 | Li et al. (2021) |
| Qutan Huazhuo Prescription (QTHZ) | *Cyperus rotundus L.* [Cyperaceae; *Cyperi rhizoma*], Citrus × aurantium L. [Rutaceae; *Aurantii fructus immaturus*], *Pinellia ternata (Thunb.) Makino* [Araceae; *Pinelliae rhizoma praeparatum*], *Poria cocos (Schw.) Wolf* [Polyporaceae; *Poria*], *Atractylodes lancea (Thunb.) DC.* [Asteraceae; *Atractylodis rhizoma*], *Wurfbainia villosa (Lour.) Skornick. & A.D.Poulsen* [Zingiberaceae; *Amomi fructus*], *Shenqu*, *Crataegus pinnatifida Bunge* [Rosaceae; *Crataegi fructus*], *Gardenia jasminoides J.Ellis* [Rubiaceae; *Gardeniae fructus*], *Conioselinum anthriscoides 'Chuanxiong'* [Apiaceae; *Chuanxiong rhizoma*]  Prepared by Li et al. (2022) according to China Pharmacopoeia | 10:8:9:10:10:5:15:15:10:9 | Li et al. (2022) |
| Quyu Huatan Tongmai Prescription (QYHTTM) | *Salvia miltiorrhiza Bunge* [Lamiaceae; *Salviae miltiorrhizae radix et rhizoma*], *Conioselinum anthriscoides 'Chuanxiong'* [Apiaceae; *Chuanxiong rhizoma*], *Panax ginseng C.A.Mey.* [Araliaceae; *Ginseng radix et rhizoma*], *Crataegus pinnatifida Bunge* [Rosaceae; *Crataegi fructus*], *Coptis chinensis Franch.* [Ranunculaceae; *Coptidis rhizoma*], *Alisma plantago-aquatica subsp. orientale (Sam.) Sam.* [Alismataceae; *Alismatis rhizoma*], *Hongqu*  Purchased from Beijing Kangren tang Pharmaceutical Co., Ltd | NA | Miao et al. (2022) |
| Shenerjiangzhi Formulation (SEJZ) | *Eleutherococcus senticosus (Rupr. & Maxim.) Maxim.* [Araliaceae; *Acanthopanacis senticosi radix et rhizoma seu caulis*], *Crataegus pinnatifida Bunge* [Rosaceae; *Crataegi fructus*], *muer*, *Lonicera japonica Thunb.* [Caprifoliaceae; *Lonicerae japonicae flos*]  Prepared by Zhang et al. (2022) according to China Pharmacopoeia | 6:4:4:3 | Zhang et al. (2022) |
| Shenling baizhu Powder (SLBZ) | *Panax ginseng C.A.Mey.* [Araliaceae; *Ginseng radix et rhizoma*], *Poria cocos (Schw.) Wolf* [Polyporaceae; *Poria*], *Atractylodes macrocephala Koidz.* [Asteraceae; *Atractylodis macrocephalae rhizoma*], *Dioscorea oppositifolia L.* [Dioscoreaceae; *Dioscoreae rhizoma*], *Lablab purpureus subsp. purpureus* [Fabaceae; *Lablab semen album*], *Nelumbo nucifera Gaertn.* [Nelumbonaceae; *Nelumbinis semen*], *Glycyrrhiza glabra L.* [Fabaceae; *Glycyrrhizae radix et rhizoma*], *Coix lacryma-jobi var. ma-yuen (Rom.Caill.) Stapf* [Poaceae; *Coicis semen*], *Platycodon grandiflorus (Jacq.) A.DC.* [Campanulaceae; *Platycodonis radix*], *Wurfbainia villosa (Lour.) Skornick. & A.D.Poulsen* [Zingiberaceae; *Amomi fructus*]  Prepared by Zhang et al. (2018) according to China Pharmacopoeia | 5:5:5:5:4:3:3:3:2:2 | Zhang et al. (2018) |
| Shenlingbaizhu Powder (SLBZ) | *Panax ginseng C.A.Mey.* [Araliaceae; *Ginseng radix et rhizoma*], *Dioscorea oppositifolia L.* [Dioscoreaceae; *Dioscoreae rhizoma*], *Coix lacryma-jobi var. ma-yuen (Rom.Caill.) Stapf* [Poaceae; *Coicis semen*], *Poria cocos (Schw.) Wolf* [Polyporaceae; *Poria*], *Wurfbainia villosa (Lour.) Skornick. & A.D.Poulsen* [Zingiberaceae; *Amomi fructus*], *Lablab purpureus subsp. purpureus* [Fabaceae; *Lablab semen album*], *Glycyrrhiza glabra L.* [Fabaceae; *Glycyrrhizae radix et rhizoma*], Citrus × aurantium L. [Rutaceae; *Aurantii fructus immaturus*], *Atractylodes macrocephala Koidz.* [Asteraceae; *Atractylodis macrocephalae rhizoma*], *Nelumbo nucifera Gaertn.* [Nelumbonaceae; *Nelumbinis semen*]  Purchased from Beijing Tongrentang Pharmaceutical Factory | NA | Hong et al. (2021) |
| Si Miao Formula (SMF) | *Atractylodes lancea (Thunb.) DC.* [Asteraceae; *Atractylodis rhizoma*], *Phellodendron chinense C.K.Schneid.* [Rutaceae; *Phellodendri chinensis cortex*], *Achyranthes bidentata Blume* [Amaranthaceae; *Achyranthis bidentatae radix*], *Coix lacryma-jobi var. ma-yuen (Rom.Caill.) Stapf* [Poaceae; *Coicis semen*]  Prepared by Han et al. (2021) according to China Pharmacopoeia | 2:1:2:1 | Han et al. (2021) |
| Si Ni San (SNS) | *Bupleurum chinense DC.* [Apiaceae; *Bupleuri radix*], *Paeonia lactiflora Pall.* [Paeoniaceae; *Paeoniae radix alba*], *Citrus × aurantium L.* [Rutaceae; *Aurantii fructus immaturus*], *Glycyrrhiza glabra L.* [Fabaceae; *Glycyrrhizae radix et rhizoma*]  Prepared by Zhu et al. (2019) according to China Pharmacopoeia | 1:1:1:1 | Zhu et al. (2019) |
| Tanyutongzhi Formula (TYTZ) | *T**richosanthes kirilowii Maxim.* [Cucurbitaceae; *Trichosanthis fructus*], *Allium chinense G.Don* [Amaryllidaceae; *Allii macrostemonis bulbus*]，*Baijiu*  Prepared by Wang et al. (2016) according to China Pharmacopoeia | NA | Wang et al. (2016) |
| Tian Huang Formula (THF) | *Panax notoginseng (Burkill) F.H.Chen* [Araliaceae; *Notoginseng radix et rhizoma*], *Coptis chinensis Franch.* [Ranunculaceae; *Coptidis rhizoma*]  Prepared by Yang et al. (2022) according to China Pharmacopoeia | 6:5 | Yang et al. (2022) |
| Tian Huang Formula (THF) | *Panax notoginseng (Burkill) F.H.Chen* [Araliaceae; *Notoginseng radix et rhizoma*], *Coptis chinensis Franch.* [Ranunculaceae; *Coptidis rhizoma*]  Prepared by Pang et al. (2021) according to China Pharmacopoeia | 6:5 | Pang et al. (2021) |
| Tongmai Zhuyu Decoction (TMZY) | *Rehmannia glutinosa (Gaertn.) DC.* [Orobanchaceae; *Rehmanniae radix*], *Prunus persica (L.) Batsch* [Rosaceae; *Persicae semen*], *Carthamus tinctorius L.* [Asteraceae; *Carthami flos*], *Paeonia lactiflora Pall.* [Paeoniaceae; *Paeoniae radix rubra*], *Conioselinum anthriscoides 'Chuanxiong'* [Apiaceae; *Chuanxiong rhizoma*], *Angelica sinensis (Oliv.) Diels* [Apiaceae; *Angelicae sinensis radix*], *Achyranthes bidentata Blume* [Amaranthaceae; *Achyranthis bidentatae radix*], *Crataegus pinnatifida Bunge* [Rosaceae; *Crataegi fructus*], Citrus × aurantium L. [Rutaceae; *Aurantii fructus immaturus*], *Alisma plantago-aquatica subsp. orientale (Sam.) Sam.* [Alismataceae; *Alismatis rhizoma*], *Reynoutria multiflora (Thunb.) Moldenke* [Polygonaceae; *Polygoni multiflori radix*]  Prepared by Ji et al. (2020) according to China Pharmacopoeia | 9:12:9:6:4.5:9:9:6:6:9:9 | Ji et al. (2020) |
| Xiangsha Liujunzi Decoction (XSLJZ) | *Dolomiaea costus (Falc.) Kasana & A.K.Pandey* [Asteraceae; *Aucklandiae radix*], *Wurfbainia villosa (Lour.) Skornick. & A.D.Poulsen* [Zingiberaceae; *Amomi fructus*], *Codonopsis pilosula (Franch.) Nannf.* [Campanulaceae; *Codonopsis radix*], *Poria cocos (Schw.) Wolf* [Polyporaceae; *Poria*], *Atractylodes macrocephala Koidz.* [Asteraceae; *Atractylodis macrocephalae rhizoma*], *Pinellia ternata (Thunb.) Makino* [Araceae; *Pinelliae rhizoma praeparatum*], Citrus × aurantium L. [Rutaceae; *Aurantii fructus immaturus*], *Glycyrrhiza glabra L.* [Fabaceae; *Glycyrrhizae radix et rhizoma*]  Prepared by Wang et al. (2022) according to China Pharmacopoeia | 6:3:12:12:12:6:6:6 | Wang et al. (2022) |
| Xiaoyao San (XYS) | *Bupleurum chinense DC.* [Apiaceae; *Bupleuri radix*], *Angelica sinensis (Oliv.) Diels* [Apiaceae; *Angelicae sinensis radix*], *Atractylodes macrocephala Koidz.* [Asteraceae; *Atractylodis macrocephalae rhizoma*], *Poria cocos (Schw.) Wolf* [Polyporaceae; *Poria*], *Glycyrrhiza glabra L.* [Fabaceae; *Glycyrrhizae radix et rhizoma*], *Zingiber officinale Roscoe* [Zingiberaceae; *Zingiberis rhizoma recens*], *Mentha canadensis L.* [Lamiaceae; *Menthae haplocalycis herba*], *Paeonia lactiflora Pall.* [Paeoniaceae; *Paeoniae radix alba*]  Prepared by Zhou et al. (2020) according to China Pharmacopoeia | 9:9:9:9:4.5:3:3:9 | Zhou et al. (2020) |
| Xiexin Decoction (XXD) | *Rheum palmatum L.* [Polygonaceae; *Rhei radix et rhizoma*], *Coptis chinensis Franch.* [Ranunculaceae; *Coptidis rhizoma*], *Scutellaria baicalensis Georgi* [Lamiaceae; *Scutellariae radix*]  Prepared by Chen et al. (2021) according to China Pharmacopoeia | 10:5:5 | Chen et al. (2021) |
| Xiexin Decoction (XXD) | *Rheum palmatum L.* [Polygonaceae; *Rhei radix et rhizoma*], *Coptis chinensis Franch.* [Ranunculaceae; *Coptidis rhizoma*], *Scutellaria baicalensis Georgi* [Lamiaceae; *Scutellariae radix*]  Prepared by Lei et al. (2020) according to China Pharmacopoeia | 10:5:5 | Lei et al. (2020) |
| Yinchenhao Decoction | *Artemisia capillaris Thunb.* [Asteraceae; *Artemisiae scopariae herba*], ，*Gardenia jasminoides J.Ellis* [Rubiaceae; *Gardeniae fructus*], *Rheum palmatum L.* [Polygonaceae; *Rhei radix et rhizoma*]  Prepared by Li et al. (2019) according to China Pharmacopoeia | 18:9:6 | Li et al. (2019) |
| Yunpi Huazhuo Granules (YQHZ) | *Coptis chinensis Franch.* [Ranunculaceae; *Coptidis rhizoma*], *Scutellaria baicalensis Georgi* [Lamiaceae; *Scutellariae radix*], *Curcuma aromatica Salisb.* [Zingiberaceae; *Curcumae rhizoma*], *Pinellia ternata (Thunb.) Makino* [Araceae; *Pinelliae rhizoma praeparatum*], *Zingiber officinale Roscoe* [Zingiberaceae; *Zingiberis rhizoma praeparatum*], *Codonopsis pilosula (Franch.) Nannf.* [Campanulaceae; *Codonopsis radix*], *Allium chinense G.Don* [Amaryllidaceae; *Allii macrostemonis bulbus*], *Salvia miltiorrhiza Bunge* [Lamiaceae; *Salviae miltiorrhizae radix et rhizoma*], *Paeonia lactiflora Pall.* [Paeoniaceae; *Paeoniae radix alba*], *Crataegus pinnatifida Bunge* [Rosaceae; *Crataegi fructus*], Senna tora (L.) Roxb. [Fabaceae; Cassiae semen], *Coix lacryma-jobi var. ma-yuen (Rom.Caill.) Stapf* [Poaceae; *Coicis semen*], *Conioselinum anthriscoides 'Chuanxiong'* [Apiaceae; *Chuanxiong rhizoma*], *Glycyrrhiza glabra L.* [Fabaceae; *Glycyrrhizae radix et rhizoma*]  Prepared by Kou et al. (2022) according to China Pharmacopoeia | NA | Kou et al. (2022) |
| Zexie Tang (ZXT) | *Alisma plantago-aquatica subsp. orientale (Sam.) Sam.* [Alismataceae; *Alismatis rhizoma*], *Atractylodes macrocephala Koidz.* [Asteraceae; *Atractylodis macrocephalae rhizoma*]  Prepared by Xu et al. (2017) according to China Pharmacopoeia | 5:2 | Xu et al. (2017) |
| proprietary Chinese medicines | | | |
| Compound Danshen Dripping Pills | *Salvia miltiorrhiza Bunge* [Lamiaceae; *Salviae miltiorrhizae radix et rhizoma*], *Panax notoginseng (Burkill) F.H.Chen* [Araliaceae; *Notoginseng radix et rhizoma*], *Bingpian*  Purchased from Tianjin Tianshili Pharmaceutical Group Co., Ltd | NA | Zhang et al. (2020) |
| Danlou Tablet (DLT) | *Trichosanthes kirilowii Maxim.* [Cucurbitaceae; *Trichosanthis pericarpium*]; *Allium chinense G.Don* [Amaryllidaceae; *Allii macrostemonis bulbus*]; *Conioselinum anthriscoides 'Chuanxiong'* [Apiaceae; *Chuanxiong rhizoma*]; *Salvia miltiorrhiza Bunge* [Lamiaceae; *Salviae miltiorrhizae radix et rhizoma*]; *Paeonia lactiflora Pall.* [Paeoniaceae; *Paeoniae radix rubra*]  Purchased from Jilin Conair Pharmaceutical Co., Ltd | NA | Sun et al. (2020) |
| Dengzhan Shengmai Capsules (DZSM) | *Erigeron breviscapus (Vant.) Hand. - Mazz.* [Asteraceae; *Erigerontis herba*], *Panax ginseng C.A.Mey.* [Araliaceae; *Ginseng radix et rhizoma*], *Schisandra chinensis (Turcz.) Baill.* [Schisandraceae; *Schisandrae chinensis fructus*], *Ophiopogon japonicus (Thunb.) Ker Gawl.* [Asparagaceae; *Ophiopogonis radix*]  Purchased from Yunnan Biogu Pharmaceutical Co., Ltd | NA | Guo et al. (2022) |
| Guanxinning Tablet (GXNT) | *Salvia miltiorrhiza Bunge* [Lamiaceae; *Salviae miltiorrhizae radix et rhizoma*], *Conioselinum anthriscoides 'Chuanxiong'* [Apiaceae; *Chuanxiong rhizoma*]  Purchased from Chiatai Qinchunbao Pharmaceutical co., Ltd. | 1:1 | Yang et al. (2022) |
| Hugan Qingzhi Tablet (HGQZ) | *Alisma plantago-aquatica subsp. orientale (Sam.) Sam.* [Alismataceae; *Alismatis rhizoma*], *Crataegus pinnatifida Bunge* [Rosaceae; *Crataegi fructus*], *Nelumbo nucifera Gaertn.* [Nelumbonaceae; *Nelumbinis folium*], *Typha angustifolia L.* [Typhaceae; *Typhae pollen*]  Prepared by Tang et al. (2018) according to China Pharmacopoeia | 30:30:20:15 | Tang et al. (2018) |
| Hugan Qingzhi Tablet (HGQZ) | *Alisma plantago-aquatica subsp. orientale (Sam.) Sam.* [Alismataceae; *Alismatis rhizoma*], *Crataegus pinnatifida Bunge* [Rosaceae; *Crataegi fructus*], *Nelumbo nucifera Gaertn.* [Nelumbonaceae; *Nelumbinis folium*], *Typha angustifolia L.* [Typhaceae; *Typhae pollen*]  Prepared by Tang et al. (2019) according to China Pharmacopoeia | 30:30:20:15 | Tang et al. (2019) |
| Jiangan Jiangzhi Pill (JGJZ) | *Reynoutria multiflora (Thunb.) Moldenke* [Polygonaceae; *Polygoni multiflori radix*], *Poria cocos (Schw.) Wolf* [Polyporaceae; *Poria*], *Salvia miltiorrhiza Bunge* [Lamiaceae; *Salviae miltiorrhizae radix et rhizoma*], Senna tora (L.) Roxb. [Fabaceae; Cassiae semen], *Citrus × aurantium L.* [Rutaceae; *Aurantii fructus immaturus*], *Curcuma aromatica Salisb.* [Zingiberaceae; *Curcumae rhizoma*], *Crataegus pinnatifida Bunge* [Rosaceae; *Crataegi fructus*], *Alisma plantago-aquatica subsp. orientale (Sam.) Sam.* [Alismataceae; *Alismatis rhizoma*], *Nelumbo nucifera Gaertn.* [Nelumbonaceae; *Nelumbinis folium*]  Provided by the Department of Pharmacy, Tianjin Second People’s Hospital | NA | Zhao et al. (2022) |
| Naoxintong Capsule (NXT) | *Astragalus mongholicus Bunge* [Fabaceae; *Astragali radix*], *Paeonia lactiflora Pall.* [Paeoniaceae; *Paeoniae radix rubra*], *Salvia miltiorrhiza Bunge* [Lamiaceae; *Salviae miltiorrhizae radix et rhizoma*], *Angelica sinensis (Oliv.) Diels* [Apiaceae; *Angelicae sinensis radix*], *Conioselinum anthriscoides 'Chuanxiong'* [Apiaceae; *Chuanxiong rhizoma*], *Prunus persica (L.) Batsch* [Rosaceae; *Persicae semen*], *Carthamus tinctorius L.* [Asteraceae; *Carthami flos*], *Boswellia sacra Flück.* [Burseraceae; *Olibanum*], *Commiphora myrrha (T.Nees) Engl.* [Burseraceae; *Myrrha*], *Spatholobus suberectus Dunn* [Fabaceae; *Spatholobi caulis*], *Achyranthes bidentata Blume* [Amaranthaceae; *Achyranthis bidentatae radix*], *Neolitsea cassia (L.) Kosterm.* [Lauraceae; *Cinnamomi ramulus*], *Morus alba L.* [Moraceae; *Mori ramulus*], *Pheretima aspergillum (E.Perrier)* [Megascolecidae; *Pheretima*], *Buthus martensii Karsch* [Buthidae; *Scorpio*], *Hirudo nipponica Whitman* [Hirudinidae; *Hirudo*]  Purchased from Shaanxi Buchang Pharmaceutical Co., Ltd. | NA | Lu et al. (2022) |
| Shanmei Capsule (SM) | *Crataegus pinnatifida Bge.var.major N.E.Br.* [Rosaceae; *Crataegi folium*], *Rosa davurica Pall. var. davurica* [Rosaceae; *Dahurian rose fruit*]  Purchased from Shanxi Taihang Pharmaceutical Co., Ltd | NA | Du et al. (2022) |
| Tongxinluo Capsules (TXL) | *Panax ginseng C.A.Mey.* [Araliaceae; *Ginseng radix et rhizoma*], *Rehmannia glutinosa (Gaertn.) DC.* [Orobanchaceae; *Rehmanniae radix praeparata*], *Scolopendra subspinipes mutilans L. Koch* [Scolopendridae; *Scolopendra*], *Eupolyphaga sinensis Walker* [Corydidae; *Eupolyphaga steleophaga*], *Buthus martensii Karsch* [Buthidae; *Scorpio*], Cryptotympana pustulata Fabricius [Cicadidae; *Cicadae periostracum*], *Paeonia lactiflora Pall.* [Paeoniaceae; *Paeoniae radix rubra*], *Bing Pian*, *Santalum album L* [Santalaceae; *Santali albi lignum*], *Boswellia sacra Flück.* [Burseraceae; *Olibanum*], *Dalbergia odorifera T.C.Chen* [Fabaceae; *Dalbergiae odoriferae lignum*], *Ziziphus jujuba Mill.* [Rhamnaceae; *Jujubae fructus*]  Purchased from Shijiazhuang Yiling Pharmaceutical Co., Ltd. | NA | Qi et al. (2022) |
| Zhibitai Capsule (ZBT) | *Hongqi*, *Crataegus pinnatifida Bunge* [Rosaceae; *Crataegi fructus*], *Alisma plantago-aquatica subsp. orientale (Sam.) Sam.* [Alismataceae; *Alismatis rhizoma*], *Atractylodes macrocephala Koidz.* [Asteraceae; *Atractylodis macrocephalae rhizoma*]  Purchased from Chengdu Diao Jiuhong Pharmaceutical Factory | NA | Pan et al. (2020) |
