# Supplementary material for: Intestinal flora: A new target for traditional Chinese medicine to improve lipid metabolism disorders
Source: Front Pharmacol. 2023 Mar 1;14:1134430. doi: 10.3389/fphar.2023.1134430 (PMC10014879; doi:10.3389/fphar.2023.1134430)
Supplement: Supplementary file 3 [file DataSheet1.docx]

Abbreviations: ABCG5, ATP-binding cassette subfamily G member 5; AOB, Alisma orientalis Beverage; AOX, Acyl-CoA oxidase; AS, atherosclerosis; ASBT, apical sodium-dependent bile acid transporter; ASCVD, atherosclerotic cardiovascular disease; ATP-CL, ATP citrate lyase; BAs, bile acids; BAT, brown adipose tissue; BAT, brown adipose tissue; BBR, berberine; BCAAs, branched-chain amino acids; BJJW, Biejia Jian Wan; BLF, Blackberry leaf and fruit extracts; BMI, body mass index; BSEP, bile salt export pump; BSH, bile salt hydrolase; BTP, Black tea polyphenols; CA, cholic acid; CD36, cluster of differentiation 36; CDCA, chenodeoxycholic acid; CHSG, Chaihu Shugan san; CntA, carnitine oxygenase; COSM, Chitooligosaccharide; CPP, Citrus Peel Powder extract; CRP, C-reactive protein; CutC, choline-trimethylamine lyase; DCA, deoxycholic acid; DG, Diammonium glycyrrhizinate; DGSY, Danggui Shaoyao San; DXR IV, Dingxin Recipes IV; DZSM, Dengzhan Shengmai Capsules; ECD, Erchen decoction; EGCG, Epigallocatechin gallate; *F/B*, *Firmicutes/Bacteroidetes*; FABP, fatty acid synthesis protein; FAS, fatty acid synthase; FATP, fatty acid transport protein; FFA, free fatty acid; FFAR, free fatty acid receptors; FGF15, fibroblast growth factor 15; FMO, flavin-containing monooxygenase; FXR, farnesoid X receptor; GB, Ginkgolide B; GbE, Ginkgo biloba extract; GCA, glycocholic acid; GCDCA, glycochenodeoxycholic acid; GGQL, Gegen Qinlian Decoction; GLP, *Ganoderma lucidum* polysaccharide; GLP-1, glucagon-like peptide-1; GP, *Gynostemma pentaphyllum (Thunb.) Makino* [Cucurbitaceae; *Gynostemmatis herba*]; GPR, G protein-coupled receptors; GPS, Gynostemma pentaphyllum saponins; GXNT, Guanxinning Tablet; GXNT, Guanxinning Tablet; GZT, Guizhi Tang; HDL-C, high-density lipoprotein cholesterol; HFD, high-fat diet; HGQZ, Hugan Qingzhi Tablet; HLJD, Huanglian Jiedu Decoction; HYQT, Huayu Qutan Formula; HZRG, Huazhi-Rougan formula; IL, interleukin; IPTS, *Ilex pubescens* triterpenoid saponins; JGJZ, Jiangan Jiangzhi Pill; JPHZTZ, Jianpi Huazhuo Tiaozhi Granule; JPSP, Jaboticaba peel and seed powder; JPTGY, Jian Pi Tiao Gan Yin; JZG, Jiangzhi Granules; JZLG, Jiangzhi Ligan Decoction; LBPs, *Lycium barbarum* polysaccharide; LC, *Luffa cylindrica (L.) Roem* [Cucurbitaceae; *Luffa aegyptiaca Miller*]; LCA, lithocholic acid; LCBP, *L. caerulea L. berry* polyphenols; LDL-C, low-density lipoprotein cholesterol; LJP, *Laminaria japonica* polysaccharide; LMD, Lipid metabolism disorders; LPS, lipopolysaccharides; LXR-α, Liver X Receptor α; mAPS, *Astragalus mongholicus* polysaccharides; MCP-1, monocyte chemotactic protein 1; MLJ, Macroalgae Laminaria japonica; MXYG, Modified Xiongdan yinchen granules; MYCWL, Modfied Yinchen Wuling San; MyD88, Myeloid differentiation factor 88; NAFLD, non-alcoholic fatty liver disease, NFP, Noni fruit polysaccharide; NXT, Naoxintong Capsule; OST, organic solute transporter; Pae, Paeonol; PB2, Procyanidin B2; PC, Ganoderma lucidum polysaccharide and chitosan; PCP, *Penthorum chinense Pursh.* extract; PCSK9, Proprotein convertase subtilisin/kexin type 9; PLS, Pueraria lobata starch; PLS, Pueraria lobata starch; PPAR-γ, peroxisome proliferator-activated receptor γ; PPARγ, proliferator-activated receptor γ; PYOs, Porphyran-derived oligosaccharides; PYRS, Purple yam (*Dioscorea alata L.*) resistant starch; PYY, peptide tyrosine-tyrosine; QGE, Qiang Gan formula extract; QGQSHX, Qinggan Qushi Huoxie prescription; QHF, Qinghua Fang; QTHZ, Qutan Huazhuo Prescription; QXJY, Qingxin Jieyu Granule; QYHTTM, Quyu Huatan Tongmai Prescription; RC, Rhizoma Coptidis; RC, *Rhizoma Coptidis*; RCT, reverse cholesterol transport; RLPs, *Rosa Laevigata Michx. Fruits* Polysaccharides; RS, Resistant starch; RSV, Resveratrol; RTFP, Rosa roxburghii Tratt polysaccharide; SCFAs, short-chain fatty acids; SEJZ, Shenerjiangzhi formulation; SLBZ, Shenlingbaizhu Powder; SM, Shanmei Capsule; SMF, Si Miao Formula; SNS, Si Ni San; SR-A1, scavenger receptor A1; SREBP, sterol regulatory element-binding protein; ST, Senna tora (L.) Roxb. [Fabaceae; Cassiae semen]; TC, total cholesterol; TCA, taurocholic acid; TCA, taurocholic acid; TCDCA, taurochenodeoxycholic acid; TCM, traditional Chinese medicine; TG, triglyceride; TGR5, Takeda G protein-coupled receptor 5; THF, Tian Huang Formula; TLR4, Toll-like receptor 4; TMA, trimethylamine; TMAO, Trimethylamine oxide; TMAO, trimethylamine-N-oxide; TMZY, Tongmai Zhuyu decoction; TNF-α, tumor necrosis factor-α; TXL, Tongxinluo Capsule; TYTZ, Tanyutongzhi Formula; UCP2, uncoupling protein 2; UD， *Usnea diffracta Vain.* [Usneaceae; *Usnea diffracta*]; WIP, water insoluble polysaccharide from the sclerotium of *Poria cocos (Schw.) Wolf* [Polyporaceae; *Poria*]; XSLJZ, Xiangsha Liujunzi Decoction; XXD, Xiexin Decoction; XXD, Xiexin Decoction; XYS, Xiaoyao San; YQHZ, Yunpi Huazhuo granules; ZB, Zanthoxylum bungeanum Maxim. [Rutaceae; Zanthoxyli pericarpium]; ZBT, Zhibitai Capsule; ZXT, Zexie Tang.
